# Sustainable assessment in digital health interventions for primary care: A scoping review

**DOI:** 10.1177/22799036251407196

**Published:** 2026-01-23

**Authors:** Johanna Alvarez-Rodríguez, Theofanis Fotis, Bella Tomsett, Heather Baid

**Affiliations:** 1 School of Education, Sports and Health Sciences, University of Brighton, UK

**Keywords:** primary care, sustainability, primary care sustainability, digital health, mhealth, telemedicine, health technology assessment, policy-making, healthcare decision-making

## Abstract

**Background:**

Primary care is essential for improving healthcare access and global health, yet it faces challenges related to limited capacity and slow response times. Digital health interventions (DHI) (DHIs) are increasingly used to address these gaps by promoting healthy behaviours, patient empowerment, and health literacy. However, their implementation is challenged by insufficient regulations and infrastructure, and evaluations often overlook broader sustainability concerns. This scoping review examines how DHIs in primary care are assessed for sustainability across financial, social, and environmental domains.

**Methods:**

The scoping review methodology consisted of three stages: pearl-growing, keywords with operators, and reference list search. MEDLINE (PubMed and Ovid), CINAHL, IEEE Access, ScienceDirect, NICE, and TRIP databases were utilised, and the results were evaluated using qualitative content analysis.

**Results:**

The review highlights four aspects to consider when implementing digital health interventions: Enhancing health promotion and illness prevention through the user’s adherence to treatment, while addressing clinical risks. Examine social implications considering wellbeing, access, inclusion, participation, empowerment, and data protection. Consider financial impacts such as resource management, available funding, and appropriate infrastructure. And environmental implications that include product life cycle, resource use, and greenhouse emissions.

**Conclusions:**

It is recommended that guidelines for implementing DHIs in primary care prioritise improving health promotion and preventive care. Emphasising the value of building public trust by promoting well-being, ensuring human rights in data governance, addressing social determinants of health, and improving resource efficiency through interoperability and circular economy principles.

## Introduction

The future of global health care emphasises the urgent need for sustainable healthcare systems that can adapt to the persistent problems faced by global society and maintain optimal health levels worldwide.^1^ The development of primary care has positively impacted the global health care system; its service delivery model has been linked to decreased mortality rates.^2^ Primary care was proposed as an initiative to enhance access to healthcare by establishing regional health hubs.^3^ At the declaration of Alma-Ata (1978), primary care was recognised as a key aspect to achieve global health, enabling populations to have socially and productive lives; it was viewed as an opportunity to provide health care as close to individuals as possible, serving as the initial element for the continuity of the health care process.^4^ Primary care also has demonstrated efficacy in enhancing population health in many locations throughout time.^3^ But its most noted benefit has been the reduction of hospital admissions and emergency visits.^5,6^ The World Health Organisation has acknowledged that integrating a public health approach into modern primary care effectively prevents disease in local communities by enhancing prevention and promotion.^2^ Currently, primary care represents a comprehensive societal approach to health, emphasising achieving the state of optimal health and well-being by prioritising individual needs in promotion, prevention, rehabilitation, and palliative care, while also addressing health determinants and empowering individuals.^7^

Conversely, the current state of primary care faces challenges related to accessibility and response times, which indicate a constrained capacity in comparison to high demands.^1–3,8^ Efforts to address these challenges include the integration of innovative digital technologies that have impacted care delivery and transformed some healthcare treatments into digital health interventions.^1,8–10^ Digital health interventions (DHIs) are typically defined by the utilisation of information and communication technology, wireless technologies, and advanced technologies, including artificial intelligence, to achieve health and wellness.^11,12^ DHIs can be used by healthcare professionals, the public, health management workers, support staff, and data services to deliver effective health services^12^; indeed, the World Health Organisation views digital technologies as essential tools to enhance the quality of health systems, facilitating the promotion of healthy lives, and well-being for all individuals globally.^13^ Some countries have proposed increasing DHIs, as they have been shown to increase access to health care and its quality^1,10,13,14^ by improving patient communication tools, including online assessments and digital telephony, while simplifying bureaucratic processes.^13^

Research has shown that DHIs have the potential to have positive impacts on the population’s health and well-being by promoting healthy behaviours, empowering patients, and enhancing health literacy and accessibility,^12,14,15^ They also create opportunities to control symptoms and prevent the disease’s progression by minimising geographic barriers.^16^ DHIs have been used to ease communication between patients and professionals, enhance patients’ health information, and support decision-making by apps and teleconsultations.^17^ DHIs offer mental health care interventions, behavioural and medication management, post-test result reporting, post-discharge follow-ups, and standard consultations for acute concerns with either new or existing patients.^18–20^

Challenges in implementing DHIs arise from inadequate regulations and infrastructure.^9,17^ Various digital health evaluation frameworks aim to provide the necessary knowledge to support the development, implementation, and evaluation of DHIs.^21^ The most widely used and reliable evaluation criteria for integrating healthcare applications are accessibility, user experience, privacy, the truthfulness of information, clarity of purpose, technological functionality, interoperability, and developer credibility.^22^ Some argue that DHIs’ assessments have been oversimplified, focussing only on efficiency and effectiveness. Hence, it is essential to thoroughly assess digital technologies, considering social, economic, and political factors.^23^ Along with this, Alajlan and Baslyman^24^ suggest that the sustainability of DHIs entails effectively achieving health care demands by using digital technology cost-effectively; they argue that technical, organisational, physical, financial, and user engagement are essential for a realistic, sustainable evaluation. Lokmic-Tomkins et al.^25^ also stressed the importance of evaluating DHIs for environmental sustainability to ensure responsible design and enhance their impact. Therefore, the notion of sustainability is important to map out in the literature as it seems to improve the integration DHIs.

Elkington^26^ defined sustainability as thinking beyond financial forms of capital, including natural, human, and social capital. In this way, human activities in any sector of society can meet the needs of present and future generations.^27^ In other words, applying sustainability to a particular process means paying attention to the well-being of all the population involved, the society, the economy, and environmental conservation.^28^ Along with this, Mortimer et al. suggested the sustainability notion as a domain of quality in health care or sustainable quality improvement (SusQI), considering health care value broader than health outcomes relative cost and including ecological and social impacts as important resources to achieve health outcomes over time.^29^ Therefore, in this review, sustainability is defined as a concept that encompasses economic, social, and environmental impacts within the evaluation process of DHIs.

This scoping review aims to map the extent to which evaluation approaches are used to assess the sustainability notion on DHIs that are integrated into primary care, exploring the financial, social, and environmental domains. The review is organised according to:

1. Definitions of sustainability domains (financial, social, and environmental) in DHIs.
2. Measurements or evaluation items of sustainability domains (financial, social, and environmental) for integration of DHIs into primary care.

## Methods

This scoping review was developed between May 2023 and February 2024. The methodological framework for a scoping review suggested by Arksey and O’Malley^30^ was used, including the following steps:

### Identification of research question

The PCC (Population, Concept, Context) framework was used to structure the review and ensure clear congruency regarding the inclusion and exclusion criteria^31^: Population – DHIs innovators and stakeholders; Concept – sustainability (social, financial and environmental); and Context – DHIs implementation in primary care. The reviewers utilised the Sustainability in Quality Improvement (SusQI) measurement criteria as model to understand the concept of sustainability in health care.^32^ The term sustainability was intentionally excluded from the data charting process, as its meaning varies significantly across contexts and disciplines. To ensure conceptual clarity and alignment with the Sustainable Quality Improvement (SusQI) framework, this review sought to capture sustainability through its constituent dimensions. Accordingly, the search strategy employed separate terms – social, financial, and environmental – to reflect the multifaceted nature of sustainability as defined within the SusQI perspective.

### Identification of relevant studies

The pearl-growing strategy is an iterative approach to identifying relevant literature on a research topic, utilising referenced literature and indexed keywords from relevant articles to find additional literature on the same topic.^33^ Pearl-growing was used to identify search terms in relevant databases, including MEDLINE (PubMed and Ovid), Cumulative Index to Nursing and Allied Health Literature (CINAHL), IEEE Access, and ScienceDirect. Sources of unpublished studies and grey literature were searched in the Trip and NICE databases. Separate searches were conducted for the terms “environmental,” “financial,” and “social” to capture the keywords adjusted to the meaning of each term in the context of digital health technologies. The enlisted terms were used in searches by two authors (X1 and X3). In this stage, search operators such as (AND/OR) were used to expand and narrow the search. Finally, the reference list of all included sources of evidence was screened for additional studies (see Appendices I, II, and III).

Studies were included if the tool’s validation was published in the English language. To maintain feasibility and reflect only in the most current literature, only studies published from 2018 onwards were selected. On the other hand, this review extends the scope to include evaluation tool developments pre and post pandemic, as pandemic boost the adoption of technology, it was interested to know paradigm changes during this time. Along with this, 2018 seems to be relevant as the WHO resolution on global strategies for improving digital health integration was released.^34,35^ Following the search, all identified citations were assembled and uploaded into EndNote 20, and duplicates were removed. Subsequently, a pilot test, titles, and abstracts were screened by one reviewer for assessment against the inclusion criteria. Meetings were held with all authors to verify their understanding of the inclusion criteria at this stage. It was agreed that due to data scarcity, it was not possible to find a tool that evaluates all three terms of sustainability (social, environmental, and financial). Therefore, the authors agreed to include articles that integrated any two terms of sustainability in their evaluation tool.

### Study selection

Relevant sources were saved in full. The full text of the selected citation was assessed in detail against the inclusion and exclusion checklist shown in Appendix V. During the screening process, the PCC framework was applied as follows:

## Population

- Papers were included if they considered DHI innovators or stakeholders relevant to primary care.

## Concept

● Papers were included if they addressed at least two of the following aspects of sustainability taken from Mortimer et al. measurement criteria of sustainability in health care^32^:
○ Social indicators, factors concerning the impact of the integration of DHIs on the social circumstances of end users (access, wellbeing, quality of life, empowerment, safety, or trust).
○ Economic indicators, factors related to cost-effectiveness, financial impact, or procurement analysis.
○ Environmental indicators, factors related to the impact on natural resources planetary health, greenhouse gas emissions, or life cycle assessment.

## Context

● Papers were included if they referred to evaluation frameworks or guidelines to implement DHIs in primary care.

Papers were excluded based on several criteria: (1) lack of access to the authors, (2) labelling as study protocols, (3) evaluation or guidance limited to a single aspect of sustainability, (4) focus on clinical trials for developing DHIs, (5) absence of implementation guidance for DHIs, (6) recommendations restricted to technical frameworks, and (7) implementation guidance applicable only to settings other than primary care (see Appendix V and VI).

### Charting the data

**Figure 1.**
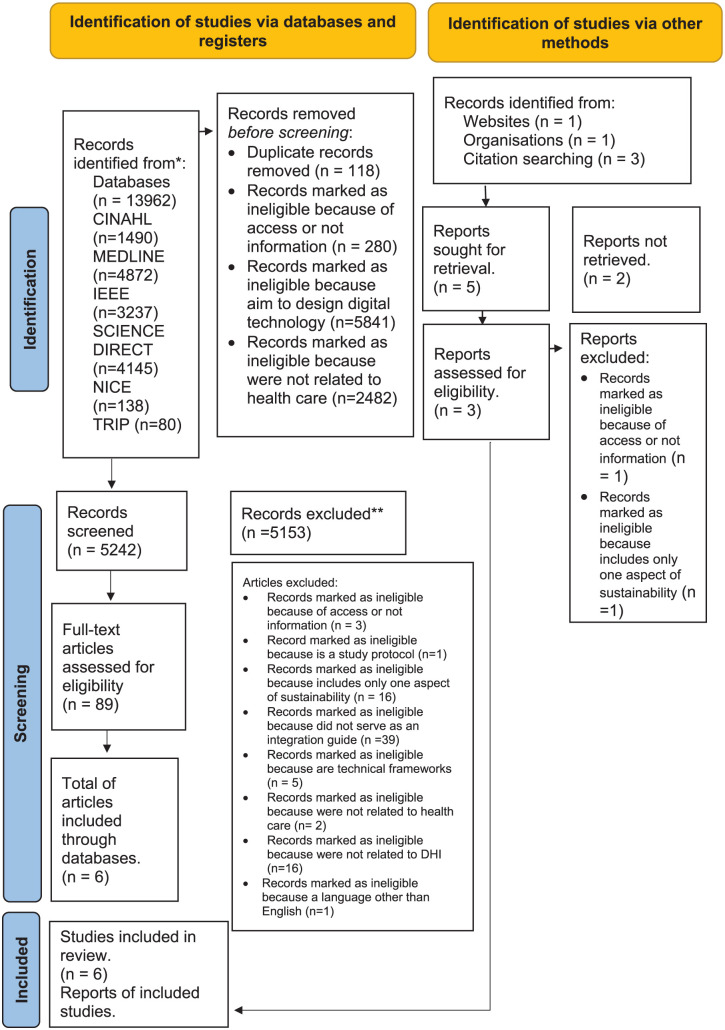
PRISMA Flow diagram for scoping review process.

The two authors conducting the literature search (X1 and X3) reviewed and screened the full text of the terms “environmental,” “financial,” and “social” in the ScienceDirect database, concluding with consensus and similar results in the screening criteria for all databases. The study inclusion process is presented in the PRISMA-ScR Flow diagram for the scoping review process in Figure 1.^36,37^ Additionally, the PRISMA-ScR flow diagram for scoping review process shows the decision process, study selection and results (see Figure 1)^38^

A pilot test was carried out prior to the full extraction to ensure that the extraction tool was appropriate for the scoping review’s goal. The extracted data contained specific information about the population, concept, context, study methods, and key findings pertinent to the review question. The draft data extraction tool underwent minor changes during the extraction process due to a scarcity of tools that comprehensively evaluated sustainable quality improvement (environmental, social, and financial); consequently, data was collected from each distinct concept within the extraction tool.

### Collating, summarising, and reporting the results

Using a qualitative content analysis approach, the scoping review mapped the guidelines or frameworks used to implement DHIs from a sustainable view (social-environmental-financial). Therefore, the results were analysed using the three phases of qualitative content analysis (preparation, organising, and reporting) and classified into the three pre-defined terms of sustainability measurement criteria in health care (social-environmental-financial).^39^ The PRISMA extension for scoping reviews (PRISMA-ScR) was used to plan and evaluate (see Appendix IV).

## Results

The electronic searches of the databases yielded 13,962 records. After removing duplicates and ineligible records, 5242 titles and abstracts were screened, resulting in 89 full-text reports being assessed for eligibility. The studies and the reason for exclusion can be found in Appendix V. Nine studies were chosen for data extraction; six were found from database searching,^40–45^ and three were found through other sources.^46–48^ The study selection process is outlined in Figure 1. All studies were published from 2020 onwards. Two publications were based in the United Kingdom^43,44^ and one in the United States.^41^ Two guidance articles incorporated inputs and comparisons from multiple countries, including the United States, the United Kingdom, Germany,^42^ and most African countries.^40^ Three frameworks were developed in Geneva and intended for use worldwide.^45,46,48^ The participants involved in the frameworks were experts in the integration of digital health^40,42,44,47^; clinicians^41,42,44^; patients or end users^42,45,46^; and decision-makers.^42,44,47^ Only two of the guidance are based on literature reviews.^43,48^ All papers provide guidance and measurements on integrating DHIs into preventive, general healthcare services and public health. Six articles were based on frameworks that evaluate the implementation of DHIs,^40–44,47^ and three studies guided the design of DHIs.^45,46,48^ Four publications were governmental guidance,^44–46,48^ and five studies were scientific articles which gathered primary and secondary data in their methodology.^40–43,47^ Four frameworks were validated,^40,42,44,47^ and one was highly used by digital health developers^44^ (To see the studies’ measurements domains, Table 1).

**Table 1.** Measurement items and domains used in the guidelines.

Concept	Measurement items	Domains
Social	Digital health empower population to seek^46^	Inclusion
	Stakeholder engagement^40^	
	Participation and empowerment^46^	
	Accessible for children youth and other groups^46^	
	Ability to patients’ users to input data^42^	
	Describe strategies for communication consent and training process to allow DHT to be understood^44^	
	Users support^42^	
	consider health and care inequalities and bias mitigation^44^	
	promoting equality, eliminating unlawful discrimination and fostering good relations between people with protected characteristics^44^	
	NHS Digital’s guide on digital inclusion for health and social care provides information for companies and providers to understand digital inclusion and steps that can be taken to evaluate and support digital inclusion^44^	
	Equitable digitally enable^44,46^	
	Individual experience^41^	
	Equity^41^	
	relevance of the solution to the targeted user group^42^	
	Ease of adoption and use with minimal training^42^	
	Multi stakeholder design, development, and implementation^42^	
	Ensure the assessed context has internet access up taken by different communities^43^	
	Non-retrogression equality and non-discrimination. To ensure compliance with legal context Laws, norms, and standards Rationale for inclusion in the assessment^43^	
	User friendly^42^	
	Government and civil society should monitor whether equity and rights are embedded with in each of the digital health building blocks^46^	
	Public involvement in the design or incorporation of the intended user group acceptability in the design^44^	
	To ensure that standard operating procedures have been established for patient consent, data protection and storage, and verifying provider licencing and credentials^43^	Data protection and rights
	Credibility with UK health care professionals^44^	
	Safety and data protection^42,43,45^	
	Health system strengthening laws, norms, and standards privacy^43^	
	To ensure that patients are aware of who owns their data and whether the data may be used by a third party, as well as to ensure their consent to such ownership and use^43^	
	Rights to information and privacy^43^	
	Privacy and informed consent future availability and access^43^	
	Information governance standards, process of data protection violation^42^	
	Privacy and confidentiality limitations^45^	
	Store secure^43^	
	Consent data protection^45^	
	Embed good data practices^42–44^	
	Are there privacy and confidentiality limitations in their current patterns of technology use (e.g. accessing the internet at internet cafes, sharing phones)?^45^	
	Data ownership^43^	
	Measuring timely access^41^	Access
	Affordability to the patient^42^	
	Contribution to challenge health inequalities^42,44,46^	
	Helps to reduce socioeconomic health inequities^42^	
	Connexion to peer support where appropriate^42^	
	Loss of work or school because of appointment^41^	Wellbeing
	Patient centredness^41^	
	Individual experience^41^	
	Monitor strategies related to promote equity and human rights empowering and building trust in digital transformation^46^	
	User satisfaction^42,44,48^	
	User feedback into the digital health in case of issues or harm^46^	
	User issues^40^	
	Evidence of stakeholder engagement appraisal process^40,44^	Clinical Safety
	Clinical and social health professionals see the tool as relevant and useful^44^	
Financial	sustainable funding^43^	Available funding
	Available funding^43^	
	Investing in equitable, digitally enabled health care governments, donors, and private investors should target and prioritise their investments in digitally enabled health systems and health care so that they contribute towards the realisation of UHC^46^	
	System affordability or support^40,42,46,47^	
	Technological systems functionality^42,44,45,47,48^	Appropriate Infrastructure
	support big data^42,46,48^	
	interoperability^42^	
	internet coverage and connectivity^40,42,46,48^	
	sustainable data architecture^42^	
	management systems^40,43^	
	Ensure the technology is deploy in systems that can support it^43^	
	technological system stability^41–43,45,46,48^	Resource management
	resources optimisation^42^	
	change management address^40^	
	Technical support^41–43,46,48^	
	current cost and later updates^42,43^	
	Human resources or human skills, processes, tools or technology resources^41,43,45–47^	
	Cost effectiveness related to outcomes (number of users using the services, user fees affect accessibility^42,44,46–48^	Economic analysis
	Sustainable business case^40^	
	Cost consequences^40^	
	Cost uncertainty^40^	
	Long-term cost effectiveness to the system User preferences, effectiveness of care, costs, safety^41^	
	Patient cost from miles saved from not travelling to the provider office^41^	
	Health delivery (quality and cost)^41^	
	Estimates of resource use should include: length of hospital or care home stay, number of hospitalisations, outpatient or primary care consultations, changes in infrastructure, use, and maintenance^44^	
	To consider who pays for training, ongoing support, and the cost of data: Is this sustainable?^43^	
Environmental	Doing no harm to the planet^46^	To consider ecological impacts
	Industry and governments should harness digital technology and data to protect the health of our planet. Proactive measures should also be taken at local, national, and global levels to mitigate any negative environmental impacts of digital transformation through, for example, use of renewable energy sources for data storage, responsible management of e-waste, and sustainable production of digital devices^46^	
	Consider environmental sustainability^44^	
Outcomes (quality)	Challenge in access^41,43–45^	Local an individual health outcome
	Contribution to inequalities mitigation^41,42,44^	
	Clinical benefits^42,44^	
	Health priorities^45^	
	Health strategies are aligned with ehealth^40,43^	
	Digital tracking of patient health status^43^	
	Contribution to Bias mitigation^44^	
	Health outcomes (measuring the individual or population level related to physiology, mental health, and quality of life; these measures may come from diagnostic tests and encounter records or from patient-reported outcomes), Mortality BMI and percentile^41^	
	Safety of telehealth visits^41^	
	All health workers, including community health workers, should have the tools, skills, and support needed to use digital technologies to assist their work in a manner complementary to the aims of quality care^46^	
	Have health care workers been consulted on whether the project aligns with the national health workforce strategy^43^	
	Could the project affect the number of health workers available to meet primary health care obligations or core obligations^43^	
	To ensure that the new technology-driven project will not draw health workers away from other essential services^43^	
	To ensure that state plans and strategies, health care management, and health care workers been consulted about the technology^43^	
**System**	Addressing the identify system challenge^48^	Systemic health outcomes

### Defining sustainability on digital health interventions

#### Clinical outcomes of digital health interventions (DHIs)

All the studies in this review illustrate the health outcome metrics and guidelines that the DHIs must achieve for successful implementation.^40–48^ Regarding primary care, which is the scope of this review, the health outcomes were related to individual health improvement and illness prevention^41–44,47^ as well as factors connected to the attainment of local and national health objectives.^40–48^ Addressing the challenges within the health system was another necessary implication for successful implementation.^40,43,45,48^ Two studies indicated that innovators require caution to ensure alignment without replacing essential components of health systems, such as health care workers.^43,46^ This aligns with the findings of study,^42^ which suggested that the integration of DHIs should provide a relevant solution to the target group while decreasing bias on the health intervention. Examples of health outcomes illustrated in the studies include lowering inequalities within the current health system^41,42,44^; enhancing quality of life for the end users^44,46^; increasing the end users’ adherence to healthcare treatment; encouraging engagement and behaviours that promote health^42,44–46^; and decreasing mortality and morbidity in the general population.^42,44^ Along with this, real-time epidemiological surveillance or real-world evidence was considered the most effective method to reduce mortality and morbidity in the integration of DHIs.^42^ Another important aspect of clinical and health outcomes is clinical safety; most studies acknowledge that the health outcomes of the DHIs should be aligned with health risk control.^41–45,48^ Six pieces of guidance on this review provide recommendations to prevent clinical risks from the DHIs to the end users.^41–45,48^ For instance, study^41^ indicates that aligning digital technology with health care system plans is a strategy to mitigate clinical risk. The study^42^ laid out the implementation of a clinical risk management plan to identify activities that could represent risks to end users. Study^44^ suggested that the DHIs integration requires accreditation from the healthcare workforce; thus, healthcare professionals should participate in the design, development, and testing processes. Lastly, there are three guidelines that suggest using an evidence-based approach to design and make clinical or health-related decisions on DHIs.^44,45,48^

### Sustainability in DHIs (social, financial, environmental impacts)

Most studies only include social and financial implications to guide DHIs implementation.^40–43,45,47,48^ Only two studies consider environmental domain as an important for integrating DHIs.^44,46^ Indeed, the ESF^44^ guidance included environmental sustainability in its recent update. Generally, the items or measurements within the social and economic domains were diverse.^40–43,45,47,48^ And the studies that include environmental domain did not include measurements.^44,46^

## Social sustainability

**Figure 2.**
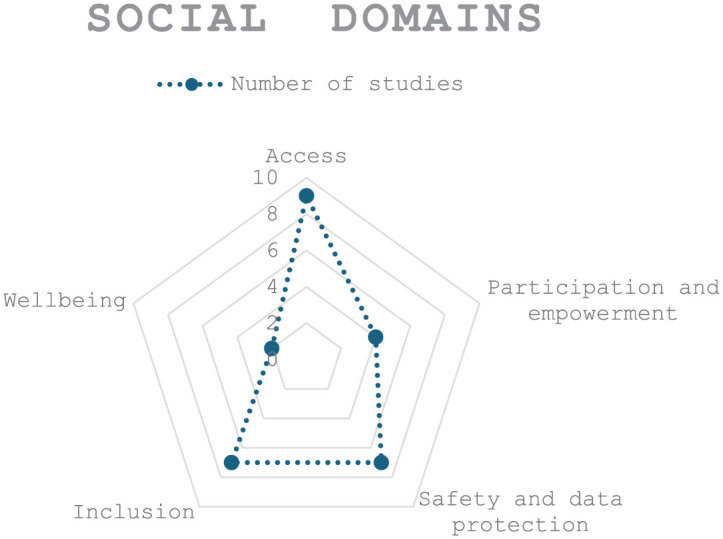
Social domains in digital health implementation.

The definition of social sustainability is complex, given the wide variety of social issues this embraces. Studies considered that the social impacts of DHIs encompass various aspects beyond health, including the impact on end users’ broader contexts, such as family and work life.^40,41^ They also include considerations of ethical and safe practices in handling data^40–43^ and increasing end users’ satisfaction.^41^ This review categorises the prevalent social issues associated with the DHIs based on an analysis of existing studies. The majority of studies in this review indicate that digital health technologies enhance access to and usability of health services,^40–47^ arguing that they foster inclusion,^40,41,46,47^ and community engagement^42–44,46,47^; they enhance overall well-being^40,46^; and they have the potential to strengthen data security and privacy in the health care system^40,42,43,45,46,48^ (see Figure 2).

### Access

According to four studies, the most common benefit noted in DHIs is the reduction of health disparities, arguing that they have improved universal health coverage within the current health care model.^40,42,43,46^ Access to DHIs in primary care may require universal health coverage.^43,45,46^ To achieve this, the development and integration of DHIs must be well-coordinated and interoperable with the healthcare system, where every element and context works harmoniously.^46^

Three studies suggested that access to DHIs should be equally available to all individuals.^43,45,46^ Indeed, four studies emphasised that access to health care is a human right that should be evaluated before implementing digital health technologies; two studies consider this important to avoid discrimination^43,45^; one study considered it an ethical issue^40^; and three studies considered that evaluation access decreases unequal delivery to vulnerable populations.^43,45,46^

### Inclusion

Five out of 10 articles showed the relevance of making technologies more inclusive.^40,43,45,46^ A study^46^ suggests that addressing issues of inclusion, equity, and human rights, along with prioritising populations with less power in the evaluation process, is essential for the successful implementation of DHIs. Along with this, study^40^ argues that inclusive technology is more than allowing the population to be connected; it is about enabling an inclusive infrastructure with adequate supplies and resources, such as electricity, software, hardware, and interoperability, to support the sharing of appropriate data.^40^ Along with this, inclusive technologies are also considered as the availability of resources in the workforce and access to health facilities for all, considering equality and non-discrimination as well as the use of appropriate terminology when describing users, depending on the specific context, considering legal, cultural, and health-related connotations.^45^ Therefore, it is suggested that a strong analysis of equity and rights during the design of digital health technologies will help decrease discrimination and inequalities when implemented.^48^

Some of the actions suggested in the studies to increase inclusion and access were related to paradigm views on the system as seen in the implementation through systemic lenses.^43,45,47,48^ For instance, three studies consider that socio-cultural factors are essential in recognising the differences throughout communities when implementing DHIs.^45,47,48^ Two studies consider that cultural and legal resources are important to integrate DHIs to avoid compromising the population’s human or civil rights.^43,48^ Other practical recommendations for ensuring accessibility when implementing DHIs included basic infrastructure such as internet or electricity,^48^ as well as meaningful community participation in all stages of digital technology development,^43,44,46–48^ including engagement with all stakeholders.^48^ In addition, a human-centred design was suggested as an essential approach to inclusion.^45^

### Participation and empowerment

Most studies recommended that when implementing digital health technologies, it is vital to place the user as the centre of the design process, focussing not only on concern but also on the user’s engagement.^40,41,43–47^ The engagement process ensures that any expectations regarding digital health technology are considered^47^ and that users actively participate in the co-creation process in which power is shared respectively and acknowledged.^45^ Engaging the population means that the relevant stakeholders, such as community leaders, international partners, non-governmental organisations, and faith-based groups, are included.^47^ It is essential to recognise end users’ participation, as it is necessary for success during implementation.^43^ Empowering individuals entails improving digital literacy,^46^ including skills and knowledge.^45–47^ By fostering digital literacy, individuals are better equipped to make conscious decisions regarding digital technologies.^43,47^ Participation is also an important feature of empowerment,^45^ positioning users at the centre of the design process.^40,41,43–47^ This approach ensures that power dynamics are shared between beneficiaries and developers throughout the development of DHIs, allowing for the valuable contributions of users to be recognised and integrated into the final product.^43,45^

### Safety and data protection

Most publications discussed DHIs’ safety in terms of users’ data management and rights.^40,42–46^ Technical safety, cybersecurity, and privacy were the most common topics related to metrics for good practices in keeping digital data and processes safe.^42–45^ The studies recommended two ways to maintain the data protected by providing a strong technical and physical infrastructure^40–43^ and adherence to strong data protection, privacy, and confidentiality standards.^40–46,48^ Regarding technical and physical structures, studies recommended aspects related to implementing adequate information and communication technology infrastructures.^40,42,43^ Information and communication technology infrastructures in data protection are related to deploying digital technologies in settings where data privacy and sensitive content sharing are considered with caution.^43^ In other words, assure end user’s privacy and data protection through technical help.^40–43,45^ For instance, study^42^ suggested cloud storage is safer and easier to back up. Along with this, two studies mentioned implementing end users’ technical control on data shared and data owned.^40,42^ Alternatively, implement technical features that prevent unauthorised access to health information.^42,44,48^ Most studies strongly recommended strong adherence to policies and regulations related to privacy and data protection.^40,42,43,45,46,48^ To ensure that, studies mentioned basing the DHIs’ implementation on a human rights approach.^40,42,43,45,48^ This approach includes respecting the end user’s right of privacy and information access^48^ and involves end user rights of data ownership.^42,43,46^ Therefore, it is recommended to have ethical, moral, and legal obligations related to data protection while adopting and enforcing policies to safeguard data.^45^ Study^42^ recommended a high degree of integrity in data handling,^42^ while others highlight the relevance of strictly sticking with data handling protocols.^43,44^ Study^42^ recommended ensuring regulations on end users’ data commercialisation when implementing DHIs. Building on this, three studies highlighted that end users need to have the capacity to make decisions regarding DHIs; therefore, data handling is required to be transparent, and rights on the use of DHIs and the information given need to be informed and explained. Then end users have the capacity or understanding of complex regulations to make decisions freely in regard to sharing data.^42,45,46^

### Wellbeing and quality of life

Most studies suggested that when implementing DHIs, it is relevant to consider their role in improving standards of living in the population.^40,41,47^ It is recommended that the implementation of DHIs consider individual impacts that DHIs have on end users while using the health care system^41,42,44,45,47,48^ and the way that DHIs impact social determinants or end users’ lifestyles.^40–42,46^ The study^46^ emphasised that one essential item to assess when integrating DHIs is the presence of policies that embed digital wellbeing and ensure the protection and promotion of human rights on and offline.

Regarding the individual impacts, most studies focus on end users’ satisfaction, preferences, engagement with the healthcare system^41,44,45,47,48^ and improving motivation towards users’ own health.^42^ Study^47^ recommends checking users’ perception in terms of ease, practicability, and quality of DHIs, which are also being compared with face-to-face models.^47^ On the other hand, studies,^40–42^ provide recommendations based on contextual factors such as the work or lifestyle of end users. Established systemic measurements that evaluate population wellbeing based on social determinants enable conscious decisions when implementing DHIs.^40^ On the other hand, study^41^ measures social determinants to assess satisfaction with the health system and adherence to it by considering work or study disruptions that occur when attending a healthcare appointment. On the other hand, study^42^ includes items related to how DHIs support lifestyle changes leading to improved quality of life. A key aspect considered in the guidelines for evaluating the integration of DHIs is feedback on end-user satisfaction, which suggests updating DHIs in response to social issues, particularly for marginalised populations,^46^ and determining the needs of these populations.^45^ And apply contextual questions to the evaluation when implementing DHIs.^44^

## Financial sustainability

**Figure 3.**
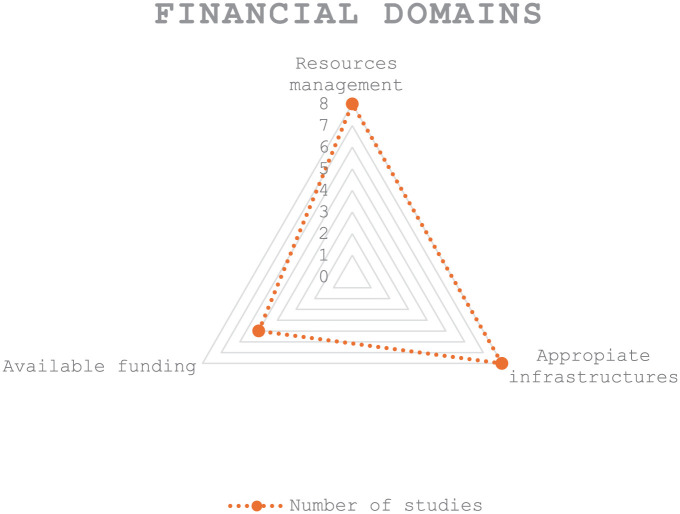
Financial domains in digital health implementation.

Financial sustainability is generally characterised by whether the investment in a particular DHI is an economically appropriate use of resources compared to other options.^40,41,44,47^ Investment analysis considers various procurement resources, such as budget availability, human resources, e-health resources, and information and communication technology infrastructure.^45,47^ Assessing economic value also requires considering end-user affordability relative to existing care models^40,41^ and evaluating the health risk-benefit ratio.^40,44^ A step forward is to recognise that the evidence for DHIs also depends on the anticipated financial risk to the system.^44^ Most studies have shown that to increase the financial sustainability of DHIs, it is important to look at resource management^41–43,45,47,48^; the reimbursement process^42,46,47^; appropriate infrastructure^42,46–48^; available funding^40,43,45,47,48^; and interoperability^42,45,47,48^ (see Figure 3).

### Resources management

A crucial aspect that the system must adapt to over time is the efficient management of resources or tools to measure the performance of new health investments.^41^ Different institutions have different resources, such as staff training^41,43,47,48^ or ongoing support.^43^ Another important aspect of resource management to be aware of is the system’s and end user’s affordability, as well as the expenses incurred by the health care system on behalf of the end user.^42^ To measure this domain, usually, an economic analysis is performed^42,44,46–48^ (see Table 1).

### Appropriate infrastructure

Countries must invest in foundational infrastructures that enable populations to use DHIs.^46,48^ For instance, improving technology infrastructures,^42,46–48^ such as internet usage, mobile network coverage, and mobile broadband connections,^46^ cybersecurity infrastructure,^42^ or pursuing interoperability.^42,45,47,48^ Interoperability is defined as the ability of systems and devices to exchange and interpret data efficiently, and it is crucial for decreasing the long-term cost of digital health implementation.^42,45,47,48^ This implies cooperation in policies, processes, and decision-making support systems that enable the rapid and effective adoption of digital technologies.^46,48^ One example of a problem related to not including interoperability when implementing digital technologies is the following: “We currently have so many systems in place, we need to find out if they are able to speak to each other and if there is a backup system.”^47^

### Available funding

Most studies considered available funding as an important domain to include when implementing digital health,^40,43,45,47,48^ which is facilitated by mapping the development and incorporating the team’s capacity, time, and budget to build the intervention.^45^ Studies also considered how transparency is required when defining who will be responsible for the cost of resources needed to implement digital technologies and who the investment allies are, such as donors, the government, or the private sector.^43,48^

## Environmental sustainability

The environmental sustainability of DHIs lies in their ability to be integrated in a way which preserves the natural world.^46^ Apart from monitoring carbon footprint, evaluating the life cycle of DHIs is an essential approach to understanding the impact on the ecosystem^46^ while also identifying social and financial benefits and challenges.^40^ To implement a DHI in primary care in a way that promotes planetary health stewardship, it is necessary to consider and integrate relevant local, national, and international policies that prioritise environmental conservation and ecological sustainability principles.^46^

**Figure 4.**
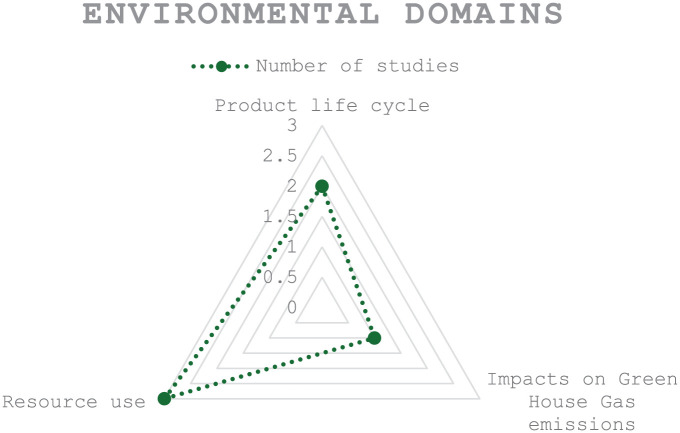
Environmental domains in digital health implementation.

Few studies mentioned environmental domains in their guidance.^44,46^ When mentioned, it was advocated that any intervention developed in healthcare must consider future generations; therefore, DHI development should not deplete natural resources but rather utilise renewable energy for data storage, manage e-waste responsibly, and employ sustainable production methods for technological devices.^46^ Other studies propose the use of reusable digital technologies for various services and purposes within the health care system, which also provide financial benefits to the system.^40,48^ Refer to Figure 4 for a diagram illustrating the key concepts related to the environmental value of DHI use in primary care.

### Measurements or evaluation of sustainability domains

The items or recommendations from the studies reviewed were compiled in Table 1 and classified according to each sustainability domain (social, environmental, and financial). In general, most studies contain items related to social domains, while there are a few items addressing the environmental domain, and the explanations for these items are often ambiguous. Only one study included another item related to health outcomes that incorporated a broader perspective when integrating DHIs, indicating the possible challenges it could pose to the health system.^48^

The concept column illustrates each dimension of sustainability. The measurement items column displays the items extracted from the evaluation tools, while the domains column classifies each sustainability dimension based on the conceptual structure established in the initial section – *Defining sustainability on digital health interventions* – (See Table 1).

## Discussion

This scoping review aims to map the existing evaluation frameworks related to sustainability in the integration of digital health interventions (DHIs) into primary care, while considering the main concepts and any overlaps or differences among these measurements. Nine guidelines and evaluation tools were included. The review extracted and synthesised the general meaning of the environmental, social, and financial aspects, which were based on the sustainable quality improvement measurements.^32^ According to Marmot,^49^ access to health must be ensured for everyone, regardless of cultural, social, economic, or physical differences, since the social determinants of health significantly affect health outcomes in the general population. Most studies in this review agreed that implementing DHIs is beneficial, as it improves access and engagement in healthcare.^40,43,46,47^ Highlighting the goal of achieving equitable health care for all kinds of people when implementing DHIs.^43,45,46^ Additionally, the common health outcomes of DHIs suggested in the evaluation tool found in this scoping review are aligned with the primary care intentions established in the Declaration of Alma-Ata^4^ and the Primary Care Recovery Plan, which aim to encourage self-care behaviours and empower individuals while reducing the burden on clinicians by improving patient communication tools and digital technology.^2^ Indeed, these are all positive health outcomes that digital health has already improved in healthcare.^9,12,14–18^ Alongside the improvement of effective and faster responses to health problems.^50^ This adheres to the recommendations of most evaluation frameworks in this review, which indicate that the integration of DHIs should focus on alleviating the burden on the healthcare system.^40,43^ However, digital health still faces some issues in equal access to health treatments and health services, as studies suggested that DHIs bring potential bias and unequal treatment for people that already experience health inequalities because of a lack of representative data from some populations.^51–53^ Additionally, the digital divide, caused by technological literacy and access disparities, remains a barrier to adopting digital technology in specific populations.^34,35^ In summary, this highlights the relevance of broadening the approach to implementing DHIs by considering social determinants and social impacts, thereby increasing the likelihood of successful implementation.

This review emphasises that preserving trust in end users is a fundamental feature of achieving social sustainability in DHIs’ implementation. Trust has been described as improving clinical safety^41–45,48^; adherence to data protection, privacy and confidentiality standards^40,42,43,45,46^; wellbeing^40–42,46^; and a human rights approach.^40,42,43,45,48^ However, other social implications could be incorporated to foster sustainable actions. For example, Mortimer et al. argues that there is no precise formula for measuring social impact due to the variety of ways healthcare delivery can influence society.^32^ Other studies argue that the core notion of social sustainability is that society maintain trust, fidelity and interconnectivity.^54^ Indeed, recognising ecological impacts during the implementation of innovative technologies relates to social sustainability, as humans are codependent on natural resources, and their preservation contributes to social justice and wellbeing.^26,29^

Mortimer et al.^29^ suggests that social and environmental issues are interdependent with health care issues, as human activities occur within these contexts. This scoping review is showing that predominant indicators on the integration of DHIs have been those related to social and economic domains.^40–43,45,47,48^ Most social indicators have emphasised end-user engagement and the adoption of digital technology as crucial for the value of digital health integration into the health system, arguing that these indicators establish its cost-effectiveness.^22,23,40,41,44,47^ This reflects the societal perception of value in actions often defined as sustainable, which is frequently measured by the capital an organisation possesses to justify the financial benefits accumulated from investments over time that required a shift.^29,32,54^

Building on this, only two evaluation frameworks recognised environmental indicators when implementing DHIs; however, the items related to environmental issues lack measurements in this dimension.^44,46^ Jeurissen and Elkington^54^ endorses the idea that industry must embed natural capital into its strategies to improve its actions towards more sustainable activities. Digital health technology should be integrated into the health system in harmony with preserving the ecosystem by aligning it with relevant local, national, and international environmental sustainability guidance.^46^ Therefore, all renewable and non-renewable resources that benefit humans, such as water, soil or energy, need to be considered,^55^ including environmentally responsible production and transport of supplies and equipment, energy usage for data storage, and e-waste management.^46^ An in-depth methodology for evaluating the environmental impact of a product or process includes a life cycle assessment of various aspects of digital health technologies, such as software, hardware, imaging, writing, text, printing reports, data storage, and virtual health services.^25^ A life cycle assessment calculates the ecosystem damage of a product or process by evaluating greenhouse gas emissions and other environmental impacts through a “cradle to grave” analysis, beginning with taking the raw materials from the Earth, through the production and transport stages, to use and waste management.^56^ However, conducting a life cycle assessment requires expertise and time and is financially costly, which may not be feasible for primary care stakeholders. Therefore, a more simplified carbon foot printing, circular economy analysis, or waste audit approach may be necessary to incorporate environmental aspects when evaluating DHIs.^40,48,57^

Research indicates that sustainability is achieved through the adoption of a systems-thinking approach that recognises the interconnectedness of social, environmental, and financial issues in human activities.^7,29,54^ However, this review identifies a gap in evaluation tools that included sustainability as the core metric for assessing DHIs in primary care, along with limited attention to the ecological measurements. Consequently, it is essential to develop more evaluation tools that acknowledge the interconnections among social, environmental, and financial impacts and establish equal importance to each domain in the implementation of DHIs.

### Strengths and limitations

The review has both strengths and limitations. This scoping review examined specific sustainable social, environmental, and financial categories for DHIs use in primary care, rather than relying solely on the ambiguous term “sustainability.” This approach acknowledges that broad perspectives on sustainability may lead to oversimplification and potentially overlook complex interconnections among various domains, as the conceptual challenge of sustainability arises from its diverse interpretations and definitions, complicating the effort to integrate these three attributes cohesively. This review analysed the interdependence of various domains to understand their diversity. Additionally, it considered government databases and extracted information from white papers, allowing a broader scope into how sustainability is seen in non-academic or governmental papers. Finally, the rigorous systematic approach enhanced transparency and replicability.

On the other hand, one of the limitations of the review is that the research selected for the review may have an inclusion bias, given that the search method predominantly relied on the English language. This may limit the incorporation of regionally unique perspectives, particularly from low- and middle-income nations where sustainability issues may differ. On the other hand, this review offers limited recommendations concerning social issues. As DHIs continue to evolve, they introduce new social dynamics and emerging challenges. Consequently, the review may not fully capture the complexity of these evolving social dimensions associated with technological advancement. Future research should build upon the foundational insights provided by this scoping review by undertaking more targeted investigations into under-explored areas and evaluating the proposed conceptual frameworks in various real-world contexts.

## Conclusion

This scoping review included nine guidelines and evaluation frameworks that identify key domains on the conceptualisation of sustainability when implementing DHIs in primary care. The expected outcomes of DHIs in primary care encompass improved healthcare outcomes and preventative interventions in the population while alleviating health care system burden and increasing the access rates. The review synthesised environmental, social and economic impacts that stakeholders should consider when implementing DHIs to achieve sustainability. The implementation of sustainable DHIs upholds trust and resource management. Maintaining trust implies emphasising welfare and human rights, particularly in data management, considering social determinants, cultural, and legal factors and the wide range of communities included in the design and implementation items that are aligned to social sustainability. While resource management integrates interoperability and the circular economy, which are essential elements for reducing waste and minimising the consumption of natural resources and healthcare costs, aspects that are in line with environmental and economic sustainability. The consequence of including these aspects in the implementation of DHIs is that it simultaneously enhances societal benefits equitably for future generations.

## Supplemental Material

sj-docx-1-phj-10.1177_22799036251407196 – Supplemental material for Sustainable assessment in digital health interventions for primary care: A scoping review Supplemental material, sj-docx-1-phj-10.1177_22799036251407196 for Sustainable assessment in digital health interventions for primary care: A scoping review by Johanna Alvarez-Rodríguez, Theofanis Fotis, Bella Tomsett and Heather Baid in Journal of Public Health Research

sj-docx-2-phj-10.1177_22799036251407196 – Supplemental material for Sustainable assessment in digital health interventions for primary care: A scoping review Supplemental material, sj-docx-2-phj-10.1177_22799036251407196 for Sustainable assessment in digital health interventions for primary care: A scoping review by Johanna Alvarez-Rodríguez, Theofanis Fotis, Bella Tomsett and Heather Baid in Journal of Public Health Research

sj-docx-3-phj-10.1177_22799036251407196 – Supplemental material for Sustainable assessment in digital health interventions for primary care: A scoping review Supplemental material, sj-docx-3-phj-10.1177_22799036251407196 for Sustainable assessment in digital health interventions for primary care: A scoping review by Johanna Alvarez-Rodríguez, Theofanis Fotis, Bella Tomsett and Heather Baid in Journal of Public Health Research

sj-pdf-4-phj-10.1177_22799036251407196 – Supplemental material for Sustainable assessment in digital health interventions for primary care: A scoping review Supplemental material, sj-pdf-4-phj-10.1177_22799036251407196 for Sustainable assessment in digital health interventions for primary care: A scoping review by Johanna Alvarez-Rodríguez, Theofanis Fotis, Bella Tomsett and Heather Baid in Journal of Public Health Research

sj-pdf-5-phj-10.1177_22799036251407196 – Supplemental material for Sustainable assessment in digital health interventions for primary care: A scoping review Supplemental material, sj-pdf-5-phj-10.1177_22799036251407196 for Sustainable assessment in digital health interventions for primary care: A scoping review by Johanna Alvarez-Rodríguez, Theofanis Fotis, Bella Tomsett and Heather Baid in Journal of Public Health Research
