# Supplementary material for: Sustainable assessment in digital health interventions for primary care: A scoping review
Source: J Public Health Res. 2026 Jan 23;15(1):22799036251407196. doi: 10.1177/22799036251407196 (PMC12833121; doi:10.1177/22799036251407196)
Supplement: sj-docx-1-phj-10.1177_22799036251407196 – Supplemental material for Sustainable assessment in digital health interventions for primary care: A scoping review [file sj-docx-1-phj-10.1177_22799036251407196.docx]

**Appendix -Scoping review**

| **Appendix I.** pearl-growing method using MEDLINE | | | | | | |
| --- | --- | --- | --- | --- | --- | --- |
| Data bases | Discipline Coverage | Main Concept | Alternative concept (mesh heading) | | Subjects’ headings | |
| Medline (Ovid) | Health | Sustainability | Conservation of natural resources                  Ecosystem | | capacities, carrying. capacity, carrying. Carrying capacities, carrying capacity, conservation, natural resources conservation of natural resources deforestation desertification, environmental protection, natural, resources conservation, protection, environmental NATURAL RESOURCES, ECOLOGY          biome, biomes, ecologic system, ecologic systems, ecological niche, ecological system, ecological systems, ecosystem, ecosystems, habitat, habitats, niche, ecological, system, ecologic, system, ecological, systems, ecologic systems, ecological, ECOLOGY. | |
|  |  | Sustainable | Sustainable development              Environmental monitoring | | development goal, sustainable, development goals, sustainable, development, sustainable, developments, sustainable, smart growth, sustainable development, sustainable development goal, sustainable development goals, **YEAR of ENTRY:** 2019; 1994-2018 see Conservation of Natural Resources      ECOLOGICAL PARAMETER MONITORING, environmental monitoring, environmental surveillance, monitoring, environmental, surveillance, environmental | |
|  |  | Digital health interventions | Telemedicine            Internet                  Mobile applications | | Telecommunications (1976-1992)-related TELEMETRY  health, mobile, mobile health, telehealth, telemedicine, ehealth, mhealth.      Computer Communication Networks (1996-1998), Related CLOUD COMPUTING, cyber space, cyberspace, internet, web, world wide, wide web, world, world wide web      SMARTPHONE, app, mobile, app, portable electronic, app, portable software App, smartphone, application, mobile application, portable electronic application, portable software applications, mobile apps, mobile apps, smartphone electronic app, portable electronic application, portable mobile app mobile application mobile applications mobile apps portable electronic app portable electronic application portable electronic applications portable electronic apps portable software app portable software application portable software applications portable software apps smartphone app smartphone apps software app, portable software application, portable | |
| **Appendix II**. Search strategy in Scopus  (First phase) | | | | | |  |
| Key concepts | | | | Search strategy | |  |
| Sustainable value (1245) | | | | “Sustainable development” (321) OR sustainability (301) OR “sustainable value” (222) OR “value creation” (67) OR “triple bottom line” (19) | |  |
| Sustainab* (874162) | | | | “Sustainable development” (229879), OR sustainability (119569), OR “climate change” (33827), OR “environmental impact” (26704). | |  |
| Digital health interventions (DHI) | | | | “digital health” (229) OR telemedicine (180), OR “digital health intervention” (136), OR mhealth (133) OR telehealth (119) OR “mobile applications” (98), OR ehealth (86) OR “digital technology’ (78), OR “Mobile phone” (73),  OR internet (64), OR mobile applications (62), OR “Mobile health” ( 56) , OR “digital health interventions” (50) | |  |

| **Appendix III.** Search strategy in Scopus (Second phase) | | | |
| --- | --- | --- | --- |
| PCC | Term | MeSH | Key words |
| Context | General terms for primary care  General practice, community pharmacy, dental, and optometry, community health, population health, public health. the aim is to provide an easily accessible route to care, whatever the patient’s problem. Primary health care is based on caring for people rather than specific diseases. This means that professionals working in general practice are generalists, dealing with a broad range of physical, psychological, and social problems, rather than specialists in a particular disease area. | primary care  exp Primary Health Care | ("primary care" or "primary health care" or physicians or "family practice" or "family medicine" or "general medicine") |
|  | Primary care specialty – family practice | exp Pharmacists/  exp Optometry/   exp General Practice, Dental/  exp Community Health Nursing/ | pharmacist* or optometry or dent* or "community health nursing" |
| Context | Digital health defined by NICE/WHO/NHS | exp Telemedicine/ or exp Internet/ or exp Mobile Applications/ or exp Information Services/ exp Cell Phone/ or exp Text Messaging/ exp Medical Informatics/ or exp Health Information Exchange/ or exp Medical Records Systems, Computerized/ exp Wearable Electronic Devices/ or exp Monitoring, Ambulatory/ or exp Monitoring, Physiologic/ | "digital health" or telemedicine or telehealth or ehealth or mhealth or videconferenc* or telecommunication* or "remote consultation" or "digital health intervention" or smartphone or phone or "digital health technology" or "text message" or wearable* or "patient monitor" or "patient web portal"  "healthcare 4.0" or "health 4.0" or "medical industry 4.0" or "health care industry 4.0" |
|  | Digital health not defined by FDA/WHO | exp Artificial Intelligence/ or exp Machine Learning/ or exp Neural Networks, Computer/exp Natural Language Processing/ | "artificial intelligence" or "machine learning" or "machine intelligence" or "computational intelligence" or "natural language processing" or "big data" |
| Concept | Sustainable development AND sustainability    The term of sustainability closer to the triple bottom line is the sustainable development | exp Sustainable Development/ | or "sustainab* or "sustainab* develop*" or "triple bottom line" or "sustainab* value" or "value creation" or "smart growth" |
|  | The concept of Sustainability will be search on the lenses of triple bottom line which is defined as the following:    **Social sustainability**  An organization that is socially sustainable seeks to address social factors that may impact on the healthy development of society.   social factors that affect health, that is, the social determinants of health which are work, social network, suitable accommodation, in health care are those factors that are beyond the symptoms, to consider social factors that bear on long term resilience, health and wellbeing  health inequalities related to social status, stress, early life disadvantage, social exclusion, poor work conditions, unemployment, lack of social support, addiction, food insecurity, and poor access to transportation  The main concepts are :  **Poverty**  **Inequalities (digital divide)**  **Safety (computer security)**  **Health care disparities**  **Health status disparities** | exp Humans/  exp Health Status Disparities/ or exp Aged/ or exp Healthcare Disparities/  Social Media/ or Social Group/ or Social Networking/   exp Digital Divide/  exp Computer Security/  exp Socioeconomic Factors/ or exp Social Class/  Sustainable Development/ | "social sustainab*" inequalit* or disparit* or "digital divide"or socioeconom* or "digital divide" or cibersecurity or "computer security"    "social sustainab*" or  inequalit* or disparit* |
|  | **Environmental sustainability**    Decrease or absence of environmental impacts preserve the ecological system for future generations, this means enhance efficiencies, reduce resource consumption and waste and measure, and monitor carbon emissions across the entire supply chain.  Some of the behaviors for this are reuse and recycle practices  Protect depletion of natural resources | exp Climate Change/ or exp "Conservation of Natural Resources"/ or exp Ecosystem/ or exp Environmental Monitoring/ or exp Environment/  exp Carbon Footprint/ or exp Greenhouse Effect/ or exp Waste Disposal, Fluid/ | Carbon foot print , greenhouse gas, climate change, global warming, environment, eco-efficiency, - energy efficiency, material efficiency resource efficiency, ecological footprint, environmental footprint, environmental managing,    ("environment* sustainab*" or "carbon footprint" or greenhouse or "green house gas*" or green or "climate change" or "global warming" or ecoefficien* or "energy efficien*" or "material efficien*" or "resource efficien*" or footprint or "waste manage*" or "life cycle assess*" or "life cycle sustainab* assess*") sustainab* or "sustainab* develop*" or "triple bottom line" or "sustainab* value" or "value creation") |
|  | **Financial sustainability**    The economic bottom line refers to the capital an organization has. In the healthcare system it implies the cost of care against the funding or the capital. | exp Economics, Dental/ or exp Economics, Medical/ or exp Economics/ or exp Economics, Pharmaceutical/ or exp Economics, Nursing/  exp Cost-Benefit Analysis/  exp "Costs and Cost Analysis"/ | ("econom* sustainab*" or econom* or financ*or cost-effectivness or "cost effectivness" or "cost analisys" or afford*) |

| Appendix IV. PRISMA -ScR check list. | | | |
| --- | --- | --- | --- |
| **SECTION** | **ITEM** | **PRISMA-ScR CHECKLIST ITEM** | **REPORTED ON PAGE #** |
| **TITLE** | | | |
| Title | 1 | Identify the report as a scoping review. | ​​Page # 1 |
| **ABSTRACT** | | | |
| Structured summary | 2 | Provide a structured summary that includes (as applicable): background, objectives, eligibility criteria, sources of evidence, charting methods, results, and conclusions that relate to the review questions and objectives. | Page # 1 |
| **INTRODUCTION** | | | |
| Rationale | 3 | Describe the rationale for the review in the context of what is already known. Explain why the review questions/objectives lend themselves to a scoping review approach. | ​​Page # 4 |
| Objectives | 4 | Provide an explicit statement of the questions and objectives being addressed with reference to their key elements (e.g., population or participants, concepts, and context) or other relevant key elements used to conceptualize the review questions and/or objectives. | ​​Page # 4, 5, 6 |
| **METHODS** | | | |
| Protocol and registration | 5 | Indicate whether a review protocol exists; state if and where it can be accessed (e.g., a Web address); and if available, provide registration information, including the registration number. | ​​Details are provided under the declarations section. |
| Eligibility criteria | 6 | Specify characteristics of the sources of evidence used as eligibility criteria (e.g., years considered, language, and publication status), and provide a rationale. | ​​Page # 6, 7, 8​ |
| Information sources* | 7 | Describe all information sources in the search (e.g., databases with dates of coverage and contact with authors to identify additional sources), as well as the date the most recent search was executed. | ​​Page # 5 and appendices I, II, and III |
| Search | 8 | Present the full electronic search strategy for at least 1 database, including any limits used, such that it could be repeated. | ​​ Appendices I, II, and III |
| Selection of sources of evidence† | 9 | State the process for selecting sources of evidence (i.e., screening and eligibility) included in the scoping review. | ​​​ Page # 5, 6, 7 |
| Data charting process‡ | 10 | Describe the methods of charting data from the included sources of evidence (e.g., calibrated forms or forms that have been tested by the team before their use, and whether data charting was done independently or in duplicate) and any processes for obtaining and confirming data from investigators. | ​​Appendix VIII. |
| Data items | 11 | List and define all variables for which data were sought and any assumptions and simplifications made. | ​​Page # 5, 6 |
| Critical appraisal of individual sources of evidence§ | 12 | If done, provide a rationale for conducting a critical appraisal of included sources of evidence; describe the methods used and how this information was used in any data synthesis (if appropriate). | ​​Critical appraisal wasn’t conducted since the authors wanted to capture the concept and use different types of sources such as white papers.​ |
| Synthesis of results | 13 | Describe the methods of handling and summarizing the data that were charted. | ​​Page # 8 |
| **RESULTS** | | | |
| Selection of sources of evidence | 14 | Give numbers of sources of evidence screened, assessed for eligibility, and included in the review, with reasons for exclusions at each stage, ideally using a flow diagram. | ​​Page # 8 |
| Characteristics of sources of evidence | 15 | For each source of evidence, present characteristics for which data were charted and provide the citations. | ​​Appendix VI |
| Critical appraisal within sources of evidence | 16 | If done, present data on critical appraisal of included sources of evidence (see item 12). | ​​ Critical appraisal wasn’t conducted since the authors wanted to capture the concept and use different types of sources such as white papers |
| Results of individual sources of evidence | 17 | For each included source of evidence, present the relevant data that were charted that relate to the review questions and objectives. | ​​Appendix VI |
| Synthesis of results | 18 | Summarize and/or present the charting results as they relate to the review questions and objectives. | ​​Page # 10, 18 and Table 1 |
| **DISCUSSION** | | | |
| Summary of evidence | 19 | Summarize the main results (including an overview of concepts, themes, and types of evidence available), link to the review questions and objectives, and consider the relevance to key groups. | ​​Page # 19, 20, |
| Limitations | 20 | Discuss the limitations of the scoping review process. | ​​Page # 22 |
| Conclusions | 21 | Provide a general interpretation of the results with respect to the review questions and objectives, as well as potential implications and/or next steps. | ​​Page # 22​ |
| **FUNDING** | | | |
| Funding | 22 | Describe sources of funding for the included sources of evidence, as well as sources of funding for the scoping review. Describe the role of the funders of the scoping review. | ​​ Details are provided under the declarations section. |

JBI = Joanna Briggs Institute; PRISMA-ScR = Preferred Reporting Items for Systematic reviews and Meta-Analyses extension for Scoping Reviews.

* Where *sources of evidence* (see second footnote) are compiled from, such as bibliographic databases, social media platforms, and Web sites.

† A more inclusive/heterogeneous term used to account for the different types of evidence or data sources (e.g., quantitative and/or qualitative research, expert opinion, and policy documents) that may be eligible in a scoping review as opposed to only studies. This is not to be confused with *information sources* (see first footnote).

‡ The frameworks by Arksey and O’Malley (6) and Levac and colleagues (7) and the JBI guidance (4, 5) refer to the process of data extraction in a scoping review as data charting*.*

§ The process of systematically examining research evidence to assess its validity, results, and relevance before using it to inform a decision. This term is used for items 12 and 19 instead of "risk of bias" (which is more applicable to systematic reviews of interventions) to include and acknowledge the various sources of evidence that may be used in a scoping review (e.g., quantitative and/or qualitative research, expert opinion, and policy document).

*From:* Tricco AC, Lillie E, Zarin W, O'Brien KK, Colquhoun H, Levac D, et al. PRISMA Extension for Scoping Reviews (PRISMAScR): Checklist and Explanation. Ann Intern Med. 2018;169:467–473. [doi: 10.7326/M18-0850](http://annals.org/aim/fullarticle/2700389/prisma-extension-scoping-reviews-prisma-scr-checklist-explanation).

Appendix V: Inclusion check list ( Please see Microsoft Excel document, sheet # 1).

Appendix VI: Data extraction instrument (See Microsoft Excel document, sheet # 2 for full data extraction)

| **Table 1.** Template of data extraction tool | | |
| --- | --- | --- |
| **Item** | **(Study 1)** | **(Study 2)** |
| Author |  |  |
| Country |  |  |
| Year |  |  |
| Aim |  |  |
| Study type/ source |  |  |
| Population |  |  |
| Sample size |  |  |
| Age |  |  |
| Gender |  |  |
| Other demographics |  |  |
| Setting |  |  |
| Concept |  |  |
| Social |  |  |
| Environmental |  |  |
| Economical |  |  |
| Outcomes (type of tool or purpose i.e., Adoption or health treatment’ s domains) |  |  |
| Gaps in research |  |  |

| Appendix VII: Data extraction instrument (S**usQI Framework)** | | | | | | | |
| --- | --- | --- | --- | --- | --- | --- | --- |
| Study | SusQI Element | **Sustainability aspects being evaluated** | | | **Conceptualisation** | **Strength** | **Weakness** |
|  |  | **Environmental** | **Social** | **Economical** |  |  |  |
|  |  |  |  |  |  |  |  |
|  |  |  |  |  |  |  |  |
|  |  |  |  |  |  |  |  |
|  |  |  |  |  |  |  |  |

Appendix VIII. Data charting (Please see at Microsoft Excel document, sheet # 3)
