# Supplementary material for: Sustainable assessment in digital health interventions for primary care: A scoping review
Source: J Public Health Res. 2026 Jan 23;15(1):22799036251407196. doi: 10.1177/22799036251407196 (PMC12833121; doi:10.1177/22799036251407196)
Supplement: sj-docx-3-phj-10.1177_22799036251407196 – Supplemental material for Sustainable assessment in digital health interventions for primary care: A scoping review [file sj-docx-3-phj-10.1177_22799036251407196.docx]

**Appendix VI: Data extraction instrument**

| **Item** | [**[1] - Broomhead et. Al. (2023)**](https://www.mdpi.com/1660-4601/20/14/6426#table_body_display_ijerph-20-06426-t0A1) | |
| --- | --- | --- |
| **Citations** | [1]. Broomhead S, Mars M, Scott RE. A New eHealth Investment Appraisal Framework for Africa: Validation. International Journal of Environmental Research and Public Health. 2023 Jul 21;20(14):6426–6. https://doi.org/10.3390/ijerph20146426 | |
| **Title** | A New eHealth Investment Appraisal Framework for Africa: Validation | |
| **Country** | South Africa framework were build based on African countries data) | |
| **Data collected** | Nov-23 | |
| **Year** | 2020 | 2023 |
| **Aim** | to identify appropriate metrics and data sources in order to judge the applicability of the Five Case Model in African eHealth settings, as an important step preceding field testing of the Five Case Model in Africa. | was to validate the new framework through a survey of international digital health experts from Africa and elsewhere and consider any refinements they proposed. |
| **Source of founding** | BMC Health service research | MDPI (International journal of environmental research and public health) |
| **Study type/ source** | Article (secondary data) data taken from available data bases and analysed | Article (primary data and previous literature review) |
| **Population** | accessible data | Digital health experts (leadership and implementation). expert in integration of digital health technologies in Africa and outside Africa |
| **Sample size** | internationally recognised metrics, and readily accessible data sources to assess the applicability of the models five cases to Africa countries | 11 |
| **Other demographics** | metrics from world health organisation, international telecommunication union and world bank (information was taken from the regional economic communities of the Africa union. | All participants have worked in LIC, LMIC, UMIC |
| **Setting** | public health government (the results illustrate in this research are to serve as a component of an ehealth impact model for Africa. The study uses African countries as model but is meant to use for other low- and middle-income countries. | Intended for use by officials working in resource-constrained settings who face the task of selecting the optimal eHealth investments despite any economics data and/or expertise limitations. Many of these officials are likely to be based in Africa working for governments, NGOs, and small companies interested in advancing eHealth |
| **Study designed** | readily accessible online data were explored to identify candidate metrics. So, the data that was chosen were accessible online, were from recognised webpage, and that at least 60% of the countries that this data were available | a validation of a previous tool by combining the literature review from previous study and expert opinion. By doing a survey which cover the following domains : development process, structure, content, completeness and utility |
|  | The first phase of this assessment tool was by using literature and the common frameworks for investment and the expert help to integrate the most important assesses to invest in ehealth. so, they collect previous tools that were relevant to evaluate ehealth (generally, that were aligned to the five cases framework at least have con component. After identifying the frameworks that align with the 5cases framework the authors integrate all the frameworks using the candidates opinions of the best metrics. (Broomhead et al 2020). this tool helps to develop further tools that incline more to economic aspects | |
| **Concept** | it has selected metrics that contribute to the strength of an ehealth product to be incorporated in a country | eHealth shouldn’t attract public investments until its probable impacts have been apprised. The main reason for estimating impact is to ensure that the benefits realised from an investment justify the costs over time for key stakeholders and rationalise the opportunity cost. this requires a value judgment tailored to local priorities such as access to services, sustainable development goals and universal health coverage |
| **Social** | Rationalise the cost opportunity. This requires a value judgement tailored to local priorities such as access to services, sustainable development goals and universal health coverage. ...there are numerous approaches to the assessment of economic impact in the health sector. ... extent beyond economic aspects to deal with broader societal impact often referred to as socioeconomic impact... ...countries with ehealth strategy and low governance score such as Nigeria should focus on governance strengthening as a foundational requirement for ehealth investment … | Decisions to use ehealth are complex. The implementations are risk, extend across several years and affect several stakeholder types including patients, healthcare workers, healthcare organisations, insurance companies, and governments…Africa lags behind other regions for most health indicators and this trend is forecast to continue. in response to these challenges, the Africa union launched "agenda 2063: the Africa we want” with the aim to establish a high standard of living associated with good health and wellbeing. however, Africa remains hampered by a significant disease burden and insufficient expenditure on healthcare... the WHO promotes a drive towards "Universal health coverage" define as all people have access to the full range of quality health services they need, when and where they need them, without financial hardship". to advance universal health coverage the world health assembly and WHO regional committee encourage member states to embrace opportunities to use Ehealth for strengthening health system. during covid 19 pandemic there was an increase in the promotion and uptake of Ehealth. digital tools were implemented for remote consultation contact tracing and patient management to support the covid 19 response... the WHO regional office for Africa encourages African countries to use Ehealth to help respond to high disease burdens despite constrained resources and there is growing recognition of the role of Ehealth in attaining UHC in Africa. this is likely to be supported by new generations of Africa health workers who use technology resources frequently and have positive attitudes towards Ehealth. nevertheless, in resource-constrained Africa countries which need UHC the most, the barriers to using Ehealth including finance and infrastructure barriers are significant. Ehealth is not possible without adequate infrastructure and connectivity, and the digital divide increases their costs...Competition for resources occurs in most countries and decision-makers need to confirm the socio-economic benefits of the possible options. under the constrained conditions describe above a robust investment case is especially important to clarify the value of ehealth when competing with other investment options. |
| **Environmental** | no mention | no mention |
| **Economical** | each decision to invest represent and opportunity cost that government must weight carefully to ensure optimal use of available funds… the main reason to estimate impact is to ensure that the benefits realised from the investment justify the costs over time for key stakeholders and rationalise the opportunity cost...the value judgement depends on local priorities such as health access and the affordability...this tool come from the five case model which is a decision making tool by comparing the value of the tool against the affordability... Mauritius' results identified four areas that, if strengthened, will improve the likelihood of successful Ehealth investment . these included updating its Ehealth strategy, addressing aspects of the GOE survey that scored poorly, growing the Mauritanian economy and lobbying for more allocation of the fiscus to health...countries with Ehealth strategy and high governance score, but lo CHE score such as Kenya Moroco and Senegal should start by focusing on the economic and finance aspects of their Ehealth programmes... the absence of recognised Ehealth impact appraisal frameworks in regular use in Africa countries increases the opportunity cost of ehealth and the risk that investments will not produce optimal net benefits... | competition for resources occurs in most countries and decision-makers need to confirm the socio-economic benefits of the possible options. Under the constrained conditions described above, a robust investment case is especially important to clarify the value of ehealth when competing with other investment options. the need to appraise ehealth investments has been recognised for more than a decade. in resource -constrained settings, where economic data and expertise may be limited, it is particularly difficult to determine whether a proposed ehealth initiative provides the best investment opportunity to strengthen health care. the current practice of economic appraisal of ehealth investments is not adequate in Africa countries. economic appraisal tools are also inadequate and there are substantial gaps in the digital health economic appraisal literature globally and in studies from Africa countries in particular. worse still most African Ehealth initiatives do not have a prior assessment of any kind, let alone economic appraisal ... ehealth investment appraisal provides a process to evaluate which information and communication technology investment in health procedures optimal net benefits. this planning process seeks the most advantageous balance between VFM and affordability while maintaining strategic alignment. it combines economic evaluation, which provides a comparative analysis of the costs and consequences of one or more interventions, with other perspectives essential for successful ehealth implementation. the WHO has noted that ti realise the potential, digital health initiatives must be part of the wider health needs and the digital health ecosystem and guided by robust strategy that integrates leadership, financial organisational, human and technological resources and is used as the basis for a costed action plan which enables coordination among multiple stakeholders. it is essential to ensure accountability for ehealth investment decisions particularly in low- and middle-income countries where ehealth investments compete with other healthcare needs for scarce resource, and where the implications of failed initiatives are magnified. therefore, a robust investment appraisal of each option is needed for satisfactory, affordable and sustainable action plan. to address this need, a new ehealth investment appraisal framework for Africa has been developed, informed by the literature. it is based by five case model and is designed for settings that lack sufficient economics expertise and dat. the proposed new eHIAF for Africa has six stages: establish a compact with key stakeholders, collect data, generate an economic model, establish affordability metrics, iterate to consider options and identify optimal investment choices, and establish a sustainable implementation...those making investment decisions in Africa countries frequently lack the economics data and / or expertise to perform adequate ehealth investment appraisals ... therefore an appropriate eHEIAF for Africa must be accessible to them and remain useful as their access to economics data and expertise grows... regarding the re-usability of outputs, a respondent suggested it is important to recognise that certain outputs delivered as part of an ehealth initiative can also enable or contribute to solutions for other sectors. for instance, digital registries or preventive health communication solutions can be easily re-used for other health applications or even cross-sectoral, e.g. e-learning...most respondents also agreed that the economic data and expertise needed to conduct ehealth investment appraisals were limited in low- and middle-income countries... |
| **Measurements** | | |
| **Social** | within the framework there are two metrics use in a indicator called workforce and governance which are the health coverage index and Ibrahim governance index that have metrics on access and human rights, HCI (which measure health and education in a population) , status of national ehealth strategy. Ibrahim governance index score (governance) | other recommendations included the extension of amortisation over the life-span of the digital health intervention when dealing with costs and outcomes, considering extending clinical effectiveness beyond clinical to include public health benefit and expanding the question on partnerships to include all relevant sectors...only one participant disagreed and suggested that the framework should deal directly with governance. further comments were to include a governance structure in stakeholder engagement, refine the framework and describe tools and approaches for implementing the framework. responders suggested that consideration given regarding how to include new developments such as AI internet of thing, confidentiality and security and to address compliance with local laws and regulations including adding pre-appraisal questions to ensure time is not wasted on an economic appraisal on a non-viable initiative. regarding resourcing respondents suggested that the framework call out human resources as it does connectivity and require a clear human resource plan... 2 attributes were proposed by respondent j: is there a data management plan? to include issues such as security, privacy and consent and have ethical and equity issues been considered and planned for. as suggestion was made to include political economy in the attribute dealing with change management... four responders suggested the eHIAF for Africa should be more inclusive of infrastructure. several suggestions were provided to extend the attribute is there adequate connectivity? such as to include IT infrastructure rather than singling out connectivity to address digital infrastructure more broadly (not connectivity alone" and to include issues such as infrastructure availability. a further comment was that the attribute should be expanded to include power availability. a further comment was that the attribute should be expanded to include power availability and hosting services and the policy enabling context including data security, privacy, confidentiality sharing and exchange to create and adequate ICT enable environment... |
| **Environmental** | no measurements on n environment | … and to consider reusability recognising that some outputs can enable or contribute to solutions for other sectors… |
| **Economical** | percentage of GDP, CHE, Rate growth of real GDP, IDI ICT suitability resources, internet penetration score, | …six responders had used either cost-benefit analysis, cost effectiveness analysis, or both. Four responders had not used either and one unsure of economic methods. Nine respondents felt that there were insufficient economics data available for LIC and MIC to conduct ehealth investment appraisals. 1 respondent felt that sufficient dt were available and referred to examples of available macro data. another was unsure and suggested that the private sector might have access to relevant data. the pattern of answers was similar regarding the availability of economics expertise with six answering that sufficient expertise was not available and 2 that it was available...simple observation of the colour coded data showed that the responses of those who had experience working with economic appraisal methods were similar to the views of the other experts... responder c suggested that procurement plans should include transition to a sustainability model and that sustainability could be separated into a group of its own.. due to its importance... |
| **Outcomes (type of tool or purpose i.e., Adoption or health treatment’ s domains)** | The summary metric provides an overall indication of a country’s eHealth investment readiness, relative to other countries. The inconsistency of data source years is a limitation, since a country’s economic condition, ICT development and eHealth development may vary from year to year. | public health universal health coverage |
| **SUSQI FRAMEWORK (this is the principles of the Centre of sustainable health care)** |  |  |
| **Prevention (SUSQI)Disease prevention and health promotion. All clinicians should be involved in prevention. Through broader advocacy and in individual patient care, specialties should aim to tackle underlying causes of disease – the social, economic and environmental determinants of health. Where possible, interventions should capture environmental co-benefits of healthy lifestyles, such as the improvements in air quality and carbon emissions from a shift to active travel.** | no | no |
| **patient empowerment and self-care (SUSQI)Patient education and empowerment. To reduce disease progression and pre-empt complications, many patients could be empowered to take on a greater role in the management of their own health and healthcare. Informed patients are also well placed to improve the coordination between clinical teams and reduce misunderstandings or duplication.** | yes | yes |
| **lean clinical pathways (SUSQI) Lean service delivery. Improving clinical decision-making in the selection and targeting of interventions will reduce lower value activities and their associated environmental impacts. Specialties can support this by describing the relevant patient pathways and providing clear, evidence-based guidance. Even where clinical input is of high value, a greater use of online records, email and telephone can reduce travel emissions by moving information in place of patients, staff and laboratory samples. Further efficiencies can follow from better integration of specialist services, such as diabetes, cardiovascular and renal care, which have a common patient base.** | yes | yes |
| **low carbon alternatives (SUSQI) Preferential use of treatment options and medical technologies with lower environmental impact. Inclusion of sustainability measures in the evaluation of medical technologies will allow service planners, clinicians and patients to choose clinically effective treatments with the best environmental profile and will encourage their further development.** | no | no |
| **Efficient resource use-improving use of energy, transport, water, waste and equipment.** |  |  |
| **Strengths** | This tool helps to identify strengths in neighbouring countries to support collaboration and to help decision-makers to invest in the best tool possible. Showing the strength and weakness of the countries in terms of governance, affordability and economic impact |  |
| **Weakness** | this tool needs validation |  |
| **Gaps in research** |  |  |
| **thoughts on this framework** | It starts from the 5-case model described in the green book, which is an instrument to evaluate new projects or programmes in the UK. but also, this is accepted in New Zealand. |  |

| **Item** | [**[2]-Chuo, J., Macy M., and Lorch S. (2020)**](https://pubmed.ncbi.nlm.nih.gov/32817398/) | [**[3]- Haig, M., et. Al., (2023)**](https://www.valueinhealthjournal.com/article/S1098-3015(23)03025-5/fulltext?_returnURL=https%3A%2F%2Flinkinghub.elsevier.com%2Fretrieve%2Fpii%2FS1098301523030255%3Fshowall%3Dtrue) |
| --- | --- | --- |
| **Citations** | [2]. Chuo J, Macy ML, Lorch SA. Strategies for Evaluating Telehealth. Pediatrics. 2020 Aug 18;146(5):e20201781. https://doi.org/10.1542/peds.2020-1781 | [3]. Haig M, Main C, Danitza Chávez, Kanavos P. A Value Framework to Assess Patient-Facing Digital Health Technologies That Aim to Improve Chronic Disease Management: A Delphi Approach. Value in Health. 2023 Oct 1;26(10):1474–84. https://doi.org/10.1016/j.jval.2023.06.008 |
| **Title** | Strategies for Evaluating Telehealth | A Value Framework to Assess Patient-Facing Digital Health Technologies That Aim to Improve Chronic Disease Management: A Delphi Approach |
| **Country** | United States | United states, United Kingdome and Germany |
| **Data collected** | Nov-23 | Nov-23 |
| **Year** | 2020 | 2023 |
| **Aim** | to synergize the above work into a single framework that researchers can use to study telehealth’s impact on patients. In the SPROUT Telehealth Evaluation and Measurement (STEM) profile | to develop a comprehensive framework to assess the value of novel patient-facing DHTs used to manage chronic diseases by eliciting stakeholder value preferences. |
| **Source of founding** | PubMed (paediatrics) | Value in health journal (ISPOR) |
| **Study type/ source** | Article (literature review) | Article (primary data) |
| **Population** | supporting paediatrics research on outcomes and utilisation of telehealth SPROUT) 12 to 19 children age that have visit in primary care | 5 stakeholder groups (patients, physicians, industry, decision makers, and influencers) the stakeholders consisted of patients, health care professionals, supply-side actors, decision makers, and policy influencers. |
| **Sample size** | Supporting Paediatric Research on Outcomes and Utilization of Telehealth (SPROUT) | 79 |
| **Other demographics** | The STEM profile is meant to communicate telehealth’s value to 4 key stakeholder groups: patients, providers, health systems, and payers | 3 countries (united state of America, United Kingdom, Germany) these three countries represent different archetypes of health system financing: taxation social insurance and a system with many private payers and a significant public sector respectively. |
| **Setting** | the SPROUT group create a framework to evaluate telehealth in paediatrics (pc) | Chronic disease management |
| **Study designed** | use the combination of previous frameworks such as NQf, work health organisation and AHRDQ concepts are organise. This is a paper for recommendations on the tools that can be used to evaluate telemedicine | The Web-Delphi approach elicited preferences and value concern of stakeholders. Delphi studies have been widely used to measure consensus since the emergence of the technique in 1960 and have been used in value framework creation in the health sector. We used the online platform Welphi to communicate with participants conduct the Delphi exercise, share preliminary results in round and extract data after each round. Welphi facilitates an online Delphi panel in which round results are collected asynchronously allowing participants to complete the exercise at their own pace. all opinions are shared anonymously with feedback incorporated iteratively through 3 rounds. |
|  |  | literature review and primary data collection from a three-round web-Delphi exercise was utilised. Literature review and primary data collection through a web-based Delphi exercise with statistical and thematic analysis to identify stakeholder value preferences in patient-facing DHTs. |
| **Concept** | **health outcomes :** measuring the individual or population level related to physiology, mental health, and quality of life; these measures may come from diagnostic tests and encounter records or from patient-reported outcomes. | literature review in this study shows that health technology assessment as an approach to evaluate digital health technologies needs to be beyond the usual aspects that are evaluating in health technology assessment |
| **Social** | Telehealth has the potential to impact the quality and cost of health care delivery, including areas of access, effectiveness, cost, safety and equity. Stays at home mandates in many communities; telehealth can help increase access and minimize the need for in person appointments. such impact can be measured at the pandemic's start in terms of the proportion of in-person appointments that were successfully converted to telehealth encounters. conversely, access may be impacted negatively in population without access to internet services, computers or smartphones technology necessary for telehealth visits. effectiveness is known typically as the extent evidence- based care is delivered reliably and consistently to patients. this care is often described by clinical recommendations. although the convenience of telehealth may afford providers more opportunities to improve patient compliance, providers must continue to follow recommended practices and not skip safeguards, as reported recently on the tendency to overprescribe antibiotics in treating upper respiratory infections when patients are seen via telemedicine...the provider and patient experience with a telehealth encounter and the logistic impact on their daily live are important considerations... the impact on provider's workload and disruption of family routines(work and school) are personal burdens that may affect appointment adherence and overall satisfaction. other experimental factors like encounter duration, video and audio quality and connectivity should also be tracked. | in doing so they can contribute to equity efficacy and quality of care improvements population health management and improve clinical decision making…DHT also have significant variation in functionality, risk profile, and value proposition, ranging from patient-facing technologies that monitor and influence individuals’ behaviours to system- facing technologies that directly affect health system - level operations data sharing and analyses. big data capabilities and artificial intelligence amplify several issues, including data bias, privacy, security and governance. the digital health ecosystem encompassed over 350000 regulated and unregulated health related apps available in 2020. many of these applications, along with other digital solutions, are seeking health system integration and coverage by healthcare budgets... conversely, the technical and security indicator complies with local data protection regulations" had the highest rated importance with 100% positive ratings, 81 % of which were "very important"...HTA value indicators continue to evolve from focusing on medical technologies safety efficacy and costs to including a broader set of criteria that include societal aspects, patient perspectives and equity in access, focusing on promoting value-based care. in this study and as part of a multistakeholder co-creation process, Delphi participants suggested indicators that assess aspects of value for DHT that go beyond traditional HTA indicators and those include in the study countries' framework. naturally the biggest differences in HTA for DHTs compared with standard HTA approaches are inherent to digital technology and data collection. the data rights and governance, technical and security and user preferences domains encompass indicators very specific to digital health. during R1, numerous responses were received in the user preferences and data rights and governance domains, which were very strongly worded and primarily from patients, illustrating how much patients value owning and inputting their health information. the data rights and governance indicator "data are user owned" was created because of these R1 responses and had consensus but this is not reflected in any study country's regulation or value assessment framework. indeed, there is no consensus around who owns certain medical data or whether it can be owned at all. in the United States the health insurance portability and accountability act (HIPPAA). classifies data rights by the entity who collects and uses the data, instead of the ability of those data to reveal sensitive information about the data subject, conversely, the general data protection regulation which relates to Germany and the united kingdom covers all personally identifiable data regardless of who collects, stores or owns them because data governance rules are classified by rights of the data subject. data protection regulation tends to focus on a patient's right to access their health information rather than the right to study countries that patients have a right to access their health data, but ownership is a different matter. ... the inability to meet traditional evidence standards increases the need to use real-world evidence (RWE) to prove value; therefore, whose intellectual property are the collected data and further who can benefit from using those data, regardless of their identifiability? ... the differences identified between stakeholder’s value preferences and the laws that govern them reflect a need for a comprehensive policy approach involving a mix of regulation and value assessment initiatives. although some indicators can be part of HTA appraisals other may require institutional interventions with data ownership being a clear example. Many questions whether digital health specific frameworks are necessary or whether existing pharmaceutical frameworks, particularly those which include social value elements, can be adopted with minor adaptations... among other issues, widespread data collection, the use of remote monitoring and RWE to meet evidence standards illustrate that DHT not only bring forward new domains of value to be assessed but also point out gaps in regulatory needs. by understanding what different stakeholders value, healthcare decision makers ca introduces policies promoting the creation of solutions that can meet the needs of multiple stakeholders. To do this facilitation of multistakeholder discussions is essential. Multistakeholder involvement in policy creation is one proven way to address varying opinions on value. In our study, the user-preferences indicator "multistakeholder design, development, and implementation" did not have consensus based on IQR because of high neutrality ratings. This reflects a notable juxtaposition: despite participating in a study based on multistakeholder co-creation, not all respondents believe that adoption of such an approach is an important criterion with which to assess DHTs. In a patient-facing DHT context this is surprising because one might think multistakeholder input in creating digital solutions would be particularly valuable. in the National institute for health and care excellence (NICE) Evidence standards framework, having relevant clinical, social care professionals involved in the design development and testing is minimum evidence standard... one might argue dissensus highlight resistance to change because people tend to be wary of changes they may not entirely understand. in the subgroup analysis, significant differences were found between country groups but not stakeholder groups. it is not surprising that disagreement seem to stem from country-specific issues and differences in system archetypes. the disagreements demonstrate a lack of shred vision about how DHTs should be assessed and potentially a lack of understanding about potential health system benefits. policy changes around incorporation of value- based care methodologies and increased patient involvement are wide-spread and will require a shift in stakeholder mindset to build rewarding market pathways for such initiatives...policies are needed that allow patients to input information credibly solve issues around data custody ownership and privacy and facilitate RWE creation for HTA and population health management initiatives. keeping in mind that DHTs required a different approach to assessment than other medical technologies, it is important to align stakeholder opinions on value characteristics to create regulations and assessment methods that will pave the way for continued digital innovation that promotes value-based healthcare for all. |
| **Environmental** | no mention | no mention |
| **Economical** | future economic analyses will be possible if programs gather data on the costs of telehealth service expansion and delivery, such as time and resources spent to credential and prepare members of the care team, establish new billing procedures and provide telehealth tools. | …DHTs have the capacity to alleviate strains caused by rising chronic disease prevalence and the associated rise in cost… in doing so they can contribute to cost optimization … health systems and HTA bodies use a number of value frameworks to evaluate innovations and allocate resources efficiently. value frameworks reflect the differing preferences of key stakeholders involved in their construction and aim to communicate essential value dimensions transparently and explicitly, ultimately supporting informed decision making. Germany is the first country in Europe with a DHT-specific pricing and reimbursement pathway for prescribed patient facing DHTs, the Diga directory. the UK national institute for health and care excellence has created and evidence standards framework for DHTs to assist local decision makers in deciding which technologies to reimburse in their integrated care system. comparatively DHT are met with considerable scepticism in many other countries with no formal reimbursement pathways. in Italy, for example there are no regulations for DHT reimbursement beyond the 2017 EU medical device legislation, and there are no DHTs marketed, used, or reimbursed to date... (three indicators had statistically significant dissensus across all respondents based on a IQR of which 2 within the economic characteristics domain and 1 in the user preferences domain...).the economic characteristics indicator "pre-marketing approval, innovation incentives exits for supply-side actors...had the lowest rated importance of the final indicators with 61%overall positive ratings. these findings illustrate that the broad stakeholder network may not perceive innovation incentives as critical as other indicators... two indicators in the economic characteristics domain also had dissensus: "...value-based care methodology" and "sustainable system improvements" this is surprising considering DHTs are well suited to support a shift to the value-based care and offer resource optimisation benefits. |
| **Measurements** | | |
| **Social** | **Health delivery :** (quality and cost) (measuring timely access, effectiveness of care, costs, safety, and equity) **Individual experience:** (measuring the quality and characteristics of the telehealth encounter itself and its impact on the individual patient or provider in terms of workload burden, satisfaction, and issues experienced with technology and logistics) (...the telehealth usability questionnaire , telemedicine satisfaction and usefulness questionnaire, patient assessment communication during the telemedicine and the net promotor score are examples of assessment tools that can be used to assess satisfaction with the provider-patient communication, technology and usefulness...) | **(a) health inequalities: "**Helps reduce social, economic and geographical health inequalities." means- A digital solution that reduces social health inequalities improves accessibility and reduces health disparities for population groups such as those with protected characteristics (race, gender, age, sexual orientation, disability, etc.), vulnerable populations (migrants, homeless population, sex workers, etc.) and/or low income populations. This includes but is not limited to clinical support tools that reduce human bias and error. At the same time, a digital solution that helps reduces geographic health inequalities improves accessibility and can be used by all patients within the system no matter their location. This can include but is not limited to remote monitoring, connectivity... "Supports Digital Literacy": The digital solution will aim to address potential communication barriers between patients and health care providers. Digital-based interventions could improve patient engagement and outcomes, e.g, monitoring after hospitalisations, however they can only be effective if all patients are able to access, use and adequately understand them...Does not exacerbate existing health inequalities: Predictive algorithms have been known to make inequalities worse, so systems need to be in place for developing and evaluating algorithms to combat these issues.  **(b) data rights and governance**: Adheres to strong information governance standards: These standards, which differ between markets, are typically set by national governments and/or international organisations. Strong information governance adheres to regulations around privacy, data governance, safety and technical specifications (CE mark) ...Data and analytics contribute toward Real-World Data collection: Real World Evidence (RWE) is the analysis of aggregated Real-World Data collected in the context of routine care. RWE can provide a holistic view of patients and solutions that may not be measured in the context of clinical trials...Rules surrounding data privacy and commercialisation of data are simply and transparently communicated to all users: Not all privacy policies are easy to understand, and this opacity can lead to distrust amongst users. Patients can control how their data is shared, including de-registering and deleting data: Solutions in which patients can control how their data is shared promote ownership of one’s own data. This does not relate to the ultimate commitment of medical information to a patient’s electronic health record but rather the option for users to retract companies’ permission to access their health data. Data processing occurs in country of use: Some digital solutions have data processing and storage in places other than the country the data was collected. The location of data processing and storage may have legal implications surrounding the management of data, analytics and security breaches. Primary data storage is cloud-based: Cloud servers can be safer and easier to backup than physical servers. **(d) economic characteristics:** Affordability to the patient, out of pocket costs may occur for some digital solutions.  **(f) user preferences: Relevance of the solution to the targeted user group: These digital solutions are focused on the targeted user group and only offer relevant services. Ease of adoption and use with minimal training: These digital solutions are easy for anyone to begin using and integrate well into their lifestyle and treatment plan. Technical and user support: This type of solution offers a contact or other form of assistance to users for queries and issues.** |
| **Environmental** |  |  |
| **Economical** | Program implementation and KPIs (key performance indicators) (measuring program performance, implementation process, and benchmarks with peer programs; measuring how the system adapted human resources, processes, and tools) which involves economic impact | **(c) technical and security:** Uses multi-factor authentication: This process for validating a user’s identity requires two or more pieces of evidence to grant user access. Systems are in place for continued product development and security updating after product release: Frequent updates allow continual product improvement and manufacturers to combat security breaches. Processes in place to prevent unauthorised access to patient and outcomes data: Processes can be in place not just to ensure the correct user is accessing the correct information but also ensure that, in transmitting data (cloud storage, Real World Evidence collection, Electronic Health Record interoperability, etc.), there are security features preventing unauthorised access. Interoperability across platforms with access available in multiple formats: Devices can push data to Electronic Health Records or shared care record systems. This can facilitate remote monitoring and other activities. Capable of working and storing data offline and then syncing when internet restored: This means data is housed locally and synced periodically when connected to the internet. This allows users to maintain access to the digital product in the case of a security breach and the online system temporarily being shut down. Convenient replacement of device consumables: Low frequency and cost to replace consumables. Examples of device consumables are insulin pumps. **(d) economic characteristics:** Affordability to the system: Affordable digital solutions offer a moderate or modest current budget impact for the health system. For example, clinical practices need to be able to afford to purchase and run new imaging software while payers must be able to afford to reimburse them. Long-term cost effectiveness to the system: Digital solutions that are cost-effective in the long term offer lower future budget impact than the comparable clinical or process improvement. This combines affordability with resource optimisation. Examples of long-term cost effectiveness include but are not limited to reductions in 30-day readmission rates, reductions in unscheduled, and improved quality of life scores. Optimises resource use: These digital solutions offer resource allocation while minimising costs or maximising benefits in the presence of constraints. For example, digital solutions that clinical visits act in a complementary manner to health care providers. This includes, but is not limited to, scheduling, capacity planning, workload coordination, use of services, data integration, among others. (Full or partial) reimbursement offered by the health system: Reimbursed solutions are those covered by insurance. For example, if payers agree to reimburse new AI-assisted imaging technologies, patients and providers could benefit from this solution. Financial incentives offered to users by the payer: Solutions with financial incentives offer users financial rewards for use. This includes but is not limited to payments for adherence and data usage. For example, insurers may offer patients a reduction in their monthly premium if they show improved adherence to therapy through a digital solution. Financial incentives offered to practitioners by the payer: These solutions offer financial incentives to the practitioner from the payer. For example, payers may offer higher reimbursement rates when clinical decision-making support tools are used. Adheres to value-based care methodology: These digital solutions adhere to value-based health methodology, which considers patient’s treatment and how well a coordinated care team can improve patient outcomes based on certain metrics. The aim of the solution is to improve patients’ experience of care, populations’ health, as well as reducing the cost of health care. Managed-entry agreements for innovators: Financial incentives for supply-side actors in pre-marketing approval stage, such as managed-entry and risk-sharing agreements. |
| **Outcomes (type of tool or purpose i.e., Adoption or health treatment’ s domains)** | The STEM profile offers a construct to define and organize telehealth measures in terms of health outcome, health delivery quality and cost, and individual experience, as well as emphasizes program implementation and benchmarks. Findings from rigorous telehealth program evaluation in these areas can be used to inform data-driven reimbursement and policy changes that encourage appropriate telehealth use, especially amid the explosion of telehealth services associated with the COVID-19 pandemic. | Decision makers can utilize this framework when assessing potential technologies to include in their health system packages. (lit… Health systems in The post - COVID-19 world have entered an age of increased reliance on digital technology whereby patient interactions with the health system are increasingly through digital health technologies and their information is digitally stored processed and transmitted. as populations continue to age and chronic diseases continue to be the leading cause of death. DHT has emerged as a potential solution particularly regarding improved diagnostics, remote monitoring and disease self-management... health systems and HTA bodies use a number of value frameworks to evaluate innovations and allocate resources efficiently. value frameworks reflect the differing preferences of key stakeholders involved in their construction and aim to communicate essential value dimensions transparently and explicitly, ultimately supporting informed decision making... in this study we develop a holistic framework for assessment of regulated patient-facing DHTs for chronic disease management by eliciting value concerns and preferences of different stakeholders. regulated DHTs are those subjected to oversight by governmental agencies and must meet certain safety and effectiveness standards before gaining marketing authorisation. technologies considered to be medical devices which are designed to diagnose or treat disease and may pose a risk to patients are likely to be subject to regulation whereas other digital technologies such as telehealth platforms used exclusively for video consultation are unlikely to be subject to regulation... patient-facing DHT include solutions classified as software as a medical device and may be used for active self-monitoring by patients as well as remote monitoring by health care professionals thus offering active data monitoring and transmission. ... these highlights yet another reason why HTA for DHTs needs to be approached differently to standard HTA: the inability to meet traditional evidence standards increases the need to use real-world evidence (RWE) to prove value; therefore, whose intellectual property are the collected data and further who can benefit from using those data, regardless of their identifiability? ...this technology- agnostic and collaborative value framework comprising 33 indicators aims to assist not only decision makers in effectively assessing DHTs but also innovators creating technologies that deliver benefit to patients and, more generally, to health systems and society. (Measurements- Evidence of clinical benefit based on clinical endpoint: Clinical endpoints directly measure whether people in clinical trials feel or function better. Examples include overall survival, disease free survival, and progression free survival. Evidence of clinical benefit based on surrogate endpoint: Surrogate endpoints are sometimes used instead of clinical endpoints in clinical trials. They tend to be used when the clinical outcome normally used takes a significantly long time to be realised (i.e., overall survival or prolonged time to therapy escalation), or it would be unethical to run a clinical trial using a clinical end point. Surrogate endpoints used in clinical trials tend to have a well-understood relationship with the clinical endpoint (i.e., blood pressure and stroke prevention). Evidence of clinical benefit based on digital technology-related endpoint: Digital innovations may offer clinical benefits beyond clinical and surrogate endpoints. Examples include an active interface, data generation, coordination of stakeholders and improved care coordination. Clinical risk management in place: Clinical risk management is a mechanism for managing exposure to risk and enabling recognition of activities which may result in negative outcomes. Supports and sustains lifestyle changes: Lifestyle changes are behaviour modifications or habit changes that encourage positive changes in a user’s life. This can include but is not limited to dietary improvements, fitness promotion, and mental health support. Improves patient adherence: These digital solutions improve adherence to a patient’s current prescribed treatment beyond lifestyle changes. Improves personal health engagement: These digital solutions promote patient motivation and involvement in their own care. |
| **SUSQI FRAMEWORK (this is the principles of the Centre of sustainable health care)** |  |  |
| **Prevention (SUSQI)Disease prevention and health promotion. All clinicians should be involved in prevention. Through broader advocacy and in individual patient care, specialties should aim to tackle underlying causes of disease – the social, economic and environmental determinants of health. Where possible, interventions should capture environmental co-benefits of healthy lifestyles, such as the improvements in air quality and carbon emissions from a shift to active travel.** | yes | yes |
| **patient empowerment and self-care (SUSQI)Patient education and empowerment. To reduce disease progression and pre-empt complications, many patients could be empowered to take on a greater role in the management of their own health and healthcare. Informed patients are also well placed to improve the coordination between clinical teams and reduce misunderstandings or duplication.** | no | yes |
| **lean clinical pathways (SUSQI) Lean service delivery. Improving clinical decision-making in the selection and targeting of interventions will reduce lower value activities and their associated environmental impacts. Specialties can support this by describing the relevant patient pathways and providing clear, evidence-based guidance. Even where clinical input is of high value, a greater use of online records, email and telephone can reduce travel emissions by moving information in place of patients, staff and laboratory samples. Further efficiencies can follow from better integration of specialist services, such as diabetes, cardiovascular and renal care, which have a common patient base.** | yes | yes |
| **low carbon alternatives (SUSQI) Preferential use of treatment options and medical technologies with lower environmental impact. Inclusion of sustainability measures in the evaluation of medical technologies will allow service planners, clinicians and patients to choose clinically effective treatments with the best environmental profile and will encourage their further development.** | no | no |
| **Efficient resource use-improving use of energy, transport, water, waste and equipment.** |  |  |
| **Strengths** | integration of sustainable values implicitly | validated tool |
| **Weakness** | this is a guidance, but it is a collection of tools that could be use in each item to evaluate. |  |
| **Gaps in research** |  |  |
| **thoughts on this framework** |  |  |

| **Item** | [**[4]-Carmel Williams (2020)**](https://www.ncbi.nlm.nih.gov/pmc/articles/PMC7762915/) | [**[5]- Graduate institute of international and development studies (2023)**](https://www.governinghealthfutures2030.org/pdf/policy-briefs/DigitalHealthFuturesReadiness.pdf) |
| --- | --- | --- |
| **Citations** | [4]. Williams C. A Health Rights Impact Assessment Guide for Artificial Intelligence Projects. Health and human rights [Internet]. 2020 [cited 2023 Oct 2];22(2):55–62. | [5]. Secretariat. Governing health futures 2030: Growing up in a digital world [Internet]. Growing up 2030 in a digital world - Governing Health Futures. 2023 [cited 2024 Oct 7]. https://www.governinghealthfutures2030.org (accessed 2024 Oct 7) |
| **Title** | A Health Rights Impact Assessment Guide for Artificial Intelligence Projects | Digital health futures readiness |
| **Country** | United Kingdome | Geneva Switzerland (each region youth network) worldwide |
| **Data collected** | Nov-23 | Nov-23 |
| **Year** | 2020 | 2021 |
| **Aim** | this paper presents an expanded tool to help states and other actors undertake a right to health impact assessment prior to implementing AI projects. The tool, informed by the WHO guideline, is a refinement of an earlier impact assessment tool of aid-funded health projects in low-resource settings.10 It accommodates additional considerations necessary when AI health projects are under development. It explores possible impacts, specifically on the right to health, moving beyond the civil and political rights most frequently associated with digital health, big data, and AI—namely, data privacy and protection, security, and algorithm transparency. It is a guide that provides a sample of the type of questions across the health system that need to be explored—but each project will need its own context-specific adjustments. | develop a framework that policy makers and implementers in digital health ecosystem can use to guide and influence decision making. That tool should encourage the universal health coverage. |
| **Source of founding** | Health and Human Rights Journal (NIH) | governing health futures web page |
| **Study type/ source** | Article (literature review) (In response, this paper presents an expanded tool to help states and other actors undertake a right to health impact assessment prior to implementing AI projects. ) | guidance - policy brief |
| **Population** | no population is a literature review perspective | young population |
| **Sample size** | N/A | doesn’t say |
| **Other demographics** | N/A | framework was developed with young people and build on existing tools and initiatives such UNESCO. |
| **Setting** | implementation of AI projects in general health population | Public health |
| **Study designed** |  |  |
|  | literature review and guidance of the literature review | this tool is based on existing tool UNESCO WORK ON INTERNET UNIVERSLITY AND young people opinions |
| **Concept** |  | based on 4 main and broad principles (1. broad DH approaches are aligned to health goals, 2. approaches to digital health are principle based 3. digital health transformation is inclusive and people centre; 4 everyone Is able to benefit from digital health transformations. |
| **Social** | The WHO 2019 guideline on DHT for strengthening the health care system provides useful provides useful indicators for assessing some of the impacts of AI on health systems, but it fails to locate the centrality of health systems to the fulfilment of the right to health. The guideline followed a resolution brought to the World Health Assembly in 2018 that recognized the value of digital technologies (including AI) and their capacity to advance universal health coverage and the SDGs... It stresses the need to evaluate the positive and negative impacts of proposed digital health technologies and to ensure that such investments do not inappropriately divert resources from alternative, nondigital approaches and thereby increase health inequities...It calls for an assessment of the health system’s ability to absorb digital interventions and warns that new technology must not jeopardize the provision of quality nondigital services in places where digital technologies cannot be deployed... It demonstrates the assessment of various applications of health-related technology based on effectiveness, acceptability, feasibility, resource use, and “gender, equity and human rights.” The guideline encourages technology developers to work with users and to think broadly about context both within and beyond the health system, as well as to consider whether a given digital health intervention will improve universal health coverage. Although human rights are included with the “gender, equity and human rights” component for impact analysis, the specific indicator selected to assess this component is limited to the technology’s impact on equity. But equity—important as it may be—is only one human rights consideration. It is also necessary to examine other key principles of the right to health when assessing health interventions...In response, this paper presents an expanded tool to help states and other actors undertake a right to health impact assessment prior to implementing AI projects...Because the health system is the core institution through which the right to health can be realized, governments and other agencies have a duty to ensure that health systems are enabled to fulfil people’s entitlements to available, accessible, acceptable, and quality health services (AAAQ)...One way to prevent a weakening of the health system while demonstrating a commitment to the progressive realization of the right to health is to carry out human rights impact assessments prior to adopting and implementing policies and programs...people and communities must also be included, as the right to health entitles them to participate in a meaningful way in the planning, delivery, and monitoring of health care and health promotion. Human rights-based approaches to health care and health projects promote the active engagement of people who will be using services, as well as the understanding that people are legally entitled to these services as a function of their right to health. Without people’s participation, health services cannot achieve AAAQ for all...A health rights impact assessment is a systematic examination of a project, undertaken prior to its implementation, to anticipate the effect that it will have on human rights and health, including and extending beyond its own project-related goals...A health rights impact assessment predicts immediate and longer-term impacts on the whole health system by examining each of the system’s component parts and assessing the ways in which the project could strengthen or weaken that component...it aims to strengthen the health system by helping design projects that will be sustainable and contribute to the protection and fulfilment of health rights...It would therefore be of great benefit to health systems and communities if technological advances could help reduce burdens on systems and health care costs while increasing accessibility and equity...Even if the technology is designed elsewhere and imported, its ongoing use requires an adequate, well-trained, and available workforce; infrastructure (including, at the very least, electricity and internet); and accessible health facilities so that the benefits of such advances are equitably available to all people. Designing data-driven technological projects for health care in low-resource settings requires a detailed understanding of their challenging contexts; otherwise, the interventions will almost certainly be inappropriate or unsustainable. It is difficult to acquire such an understanding from afar. But even locally developed AI-based technological solutions can fail to respect and protect human rights if they are not supporting the local health system in meeting the health rights of the people in its jurisdiction...it is imperative that systematic health rights impact assessments are undertaken and that they are broad enough to anticipate impacts on the health system components, as well as on civil and political rights relating to data privacy, ownership, and security...Introducing an app that can, for example, diagnose skin cancer or detect a pregnant person’s increased risk of pre-term birth, does nothing to fulfil people’s right to health entitlements, or universal health coverage, if there are no suitable treatments available for skin cancer or secondary-level obstetric services accessible to those who need them. Every component of the health system must be functioning well before a service can become equitably available, accessible, acceptable, and of good quality; and if these and other right to health features are not achieved, people’s rights cannot be fulfilled...It is not enough for developers of a new AI application to claim that their application will address one health service and will therefore “help achieve SDG3 and universal health coverage”; without a right to health impact assessment, there can be no confidence that this is a likely outcome...Similarly, all human rights are interrelated and indivisible, which means that a rights-based app assessment must look beyond the health sector to determine how the technology could also affect other rights, including those related to privacy, confidentiality, and security....1.To ensure compliance with legal context...2. To ensure that standard operating procedures have been established for patient consent, data protection and storage, and verifying provider licensing and credentials (WHO guideline)...3.**To ensure that patients are aware of who owns their data and whether the data may be used by a third party, as well as to ensure their consent to such ownership and use...4.To explore the possibility of the new technology distorting other services: Will pre-AI services remain available and supported in case new services cannot reach everyone? Will staff or resources move from other services to this new one? ...7.To** ensure the participation of the population in designing the project, its implementation, and its monitoring...13. To ensure that the data are stored securely and integrated within the health system...14.To ensure that this technology is deployed only in settings where the health system can support its implementation in an integrated manner; is used for tasks that are already defined as within the scope of practice for health workers; and is deployed in settings where concerns about data privacy and transmission of sensitive content can be addressed (WHO guideline)...15.To ensure that the context is assessed to determine the geographic range of internet access, uptake by different communities, gender use differences, and any other concerns that could increase inequitable health outcomes...16,To ensure that the technology remains accessible and available...19,To assess whether user fees will affect accessibility...20To ensure that the state is not losing ownership of data, which could cause sustainability and privacy issues in the future...22.To ensure that the state does not transfer public goods to private owners, thereby reducing state capacity to achieve full realization of the right to health...23.To ensure that there is adequate capacity within the health system to take on additional work without reducing quality or equity... | At present, countries lack approaches to digital health that are grounded in the core principles of universal health coverage (UHC) and the Sustainable Development Goals (SDG) agenda, namely: equity, solidarity, and human rights...Digital health ecosystems are developing without adequately considering the unequal distribution of power and resources that affect an individual’s or community’s access to, engagement with, and ability to benefit from digital health technologies. The absence of a strong equity and rights analysis when designing, implementing, and evaluating digital health policies and programmes can lead to ignoring or exacerbating existing health inequities and other forms of discrimination, or even creating new ones. High digital health readiness at individual and societal levels are a prerequisite for harnessing the benefits of the digital transformations in support of UHC. The ability of individuals, including young people, to harness digital transformation requires that they have the knowledge, skills, access, and agency needed to make free and informed choices and act independently in relation to the digital technology and data that is evolving around them and how it interacts with and influences their health and wellbeing. The Governing Health Futures 2030 Commission analysed existing tools for assessing a country’s digital readiness and health readiness. It found that whilst some tools include indicators to measure equity outcomes, few indicators are disaggregated, and principles of inclusion, equity, and rights are not fully embedded into the overall assessment of a country’s readiness. Existing tools are therefore insufficient for assessing the extent to which a country’s digitally enabled health ecosystem can support the achievement of UHC and maximise the health and wellbeing of young people, now and in the future. To achieve UHC, an equity and rights centred approach to digital health that prioritises those with the least power–such as children, youth, women, people with disabilities, minority groups, and marginalised communities–and that considers how actions taken today will affect subsequent generations is required from the onset. The readiness of a country to harness digital transformations in support of UHC and better health futures should therefore be assessed through an equity and rights-based lens.,...The Commission defines ‘digital health futures readiness’ as when all people and their communities, the health ecosystems they interact with, and the countries they live in are prepared, equipped, and empowered to use digital technology and data to meet personal health and wellbeing needs and to improve the health and wellbeing of the whole population...This necessitates an analysis of where control and power lie over matters of digital and technological development and who does not have a seat at the table. It also requires a deeper understanding of the intersecting forms of discrimination and inequalities that undermine the agency of people in relation to digital health. By identifying those people who are most left behind at all stages of design, implementation, and monitoring of digital approaches, policymakers can respond by addressing imbalances of power and structural barriers that prevent everyone from benefitting equally from digital transformation in health.... Such a tool should encourage progressive realisation of UHC and human rights for all countries, regardless of their stage of development and digital maturity... |
| **Environmental** | no mention | **We emphasise futures since  digital technology and data should be  harnessed in support of more equitable  health and wellbeing outcomes today,  and also for future generations** |
| **Economical** | 6.To determine how the technology will be supported when IT partners exit…11.To consider who pays for training, ongoing support, and the cost of data: Is this sustainable?...17.To make transparent who will have to bear the costs of the AI and the impacts that this will entail on that source...18,To ensure sustainable funding...24. To ensure that there is budgeted financial support for sustainability. | One well-acknowledged aspect of digital health readiness is a country’s overall level of digital development. Several initiatives have resulted in tools to measure digital readiness across countries.1 Common indicators used include internet usage, mobile network coverage, and the number of fixed and mobile broadband connections. In all these indices, a clear digital divide is visible, with countries defined as ‘least developed’—with high mortality rates and the largest shares of young people—ranking lowest, reflecting the KEY MESSAGEExisting tools for measuring digital health readiness are insufficient for assessing the extent to which a country’s approach to digital health can support the achievement of UHC and maximise the health and wellbeing of young people, now and in the future. The Governing Health Futures 2030 Commission proposes ten enablers for digital health futures readiness. It recommends that these enablers form the basis of a new way of assessing digital health readiness that encourages all actors in a digitally enabled health ecosystem to align their digital transformation efforts with their UHC and SDG goals.Digital health futures readiness \| 3ability of countries with higher incomes to invest more in the foundational infrastructure and technology required for digital health |
| **Measurements** | | |
| **Social** | LEGAL CONTEXT (1. Is the software or app compliant with relevant national and regional legal requirements, including algorithmic transparency? - 2. What protocols are in place to inform patients, gain consent, protect privacy, and store data securely? - 3. Who owns the data, and what protections are in place regarding future use, ownership, and price protections?) ...7.Has the community been consulted to assess the technology’s acceptability and accessibility?... 13.How does the project collect patient data, and can the data be integrated into patient records and the broader health information system?... 14. Does the project involve the digital tracking of patient health status and use of health services? 15. Can this technology function within the current infrastructure?... 16. How will this technology be updated?... 19. Will patients be charged user fees?20. Will the state own the data generated from the use of the technology?... 22. Who will own, manage, and protect the data collected in the project? Human rights, equity | **… A new way of assessing digital health  readiness is needed that encourages all actors  in a digitally enabled health ecosystem to  align their approach to digital transformation  with their UHC and SDG goals...**promoting human rights: Policies and programmes related to digital technology and data should  be assessed from a human rights and child rights perspective to ensure  that all rights are promoted and protected. Where such assessments  cannot be conducted at the national level, periodic reporting to human  rights treaty bodies offer an opportunity for countries to assess whether  human rights obligations are being realised in the digital environment. Children’s entitlement to additional protections both on- and offline  must also be realised. For example, governments should put additional  measures in place to protect children’s data and to safeguard children  from online harms and commercial exploitation. Further ways to use  technology and data to promote children’s rights, including the right to  health, should be explored, equitable health care: Governments, donors, and private investors should target and prioritise  their investments in digitally enabled health systems and health care  so that they contribute towards the realisation of UHC. Government  stewardship of domestic and external investments should strive to  achieve scale whilst prioritising primary health care and the needs of  the poorest and most left behind. Governments and civil society should  monitor whether equity and rights considerations are embedded within  each of the digital health building blocks and to track whether progress  is equal for different population groups,... governing for equitable health futures: Building digital health futures where UHC is achieved and all young  people flourish requires strong political leadership and collaboration  across multiple sectors and stakeholder groups. Approaches to  governance of digital technology and data must be grounded in equity  and human rights so that the benefits of digital transformation can be  realised, and the risks mitigated, for all. Governments should reflect the  specific needs and views of children, youth, and other groups at risk  of being excluded and oppressed in all relevant legislation, regulation,  and governance frameworks. Civil society groups led by youth and  marginalised communities should be resourced to independently assess  whether governance frameworks reflect their needs..., Engineering inclusive decision making, Inclusive and representative processes to develop and monitor  strategies and plans related to digital transformation and health care  are critical for promoting equity and human rights, empowering  communities, and building trust. Children’s, youth’s, and marginalised  communities’ participation in decision-making should be a regular  practice and fully resourced by relevant ministries and other digital  health actors. Their engagement should be carried out in ways that  are mutually beneficial, resourced, and built on recognition of young  people’s agency and capacity to participate in civic activities... prioritise all people in the design: Digital technologies, initiatives, and services should be designed with  and for all groups that may directly use or be indirectly affected by them.  To tackle structural inequalities and biases, technology developers  should implement design processes that place the voices and needs  of the most vulnerable, marginalised, and oppressed at the centre.  Digital health tools and services should be designed to be accessible,  relevant, and appropriate for children, youth, and other groups who  are traditionally overlooked and excluded from the design process.  Developers should also include mechanisms to collect and incorporate  user feedback so that digital tools and services can be updated in  response to unforeseen issues or harms. , increase digital health literacy: Digital health literacy is essential for young people and other groups at  risk of being left behind to fully benefit from digital transformation in  health and to navigate the digital environment safely and effectively.  Adequate digital health literacy empowers and enables individuals to  seek, find, understand, and appraise the reliability of health information  from electronic sources and apply the knowledge gained to addressing  or solving a health problem. Digital health literacy requires greater  domestic and international investment in multiple forms of literacy  (e.g. health literacy, digital literacy and civil literacy) and in foundational  knowledge and skills. Data on digital health literacy and ICT skills should  be disaggregated by governments and international organisations, such  as UNESCO, to identify equity gaps., connecting every health worker and health facility: Health facilities at all levels–from national hospitals to community  clinics–should be connected through reliable digital infrastructure that  is regularly maintained. Ministries of health and infrastructure should  prioritise connecting local-level health facilities providing primary  health care to underserved communities and putting measures in  place to protect the security of critical infrastructure and data. All  health workers, including community health workers, should have the  tools, skills, and support needed to use digital technologies to assist  their work in a manner complementary to the aims of quality care..., embedding health and wellness policies : Building on the Health in All Policies approach, governments,  international organisations, and other digital health actors should  consider the potential benefits and risks for health systems,  determinants of health, and individual health and wellbeing in all digital  and data-related policies and programmes. Particular consideration  should be given to the potential benefits and harms for children,  youth, and other vulnerable groups. Policymakers should also consider  how to reduce inequalities and tension points that may emerge or be  magnified within the population by digital transformation and explore  the opportunities of digital technology to build greater social cohesion |
| **Environmental** | NO DATA | **...… A new way of assessing digital health  readiness is needed that encourages all actors  in a digitally enabled health ecosystem to  align their approach to digital transformation  with their UHC and SDG goals**....doing no harm to the planet (Proactive measures should also be taken at local, national, and global levels to mitigate any negative environmental impacts of digital transformation through, for example, use of renewable energy sources for data storage, responsible management of e-waste, and sustainable production of digital devices). |
| **Economical** | 5.To protect against distortions in national health plans with nonprioritized health care services being introduced because technology partners seek their inclusion...the accountability counts as the economical sustainability and funs of the technology update 11. **How will health workers be trained and provided with ongoing support in their use of the technology? 17. Will the local health system have to pay for this technology (for example, after a pilot period)?...18. Has this cost been accepted by health authorities and factored into budgets?**23. Are management systems and capacities sufficiently robust to accommodate the demands of this new technology?24. Have ongoing recurrent costs and replacement of technology costs been estimated and entered into forward budgets? | ......… A new way of assessing digital health  readiness is needed that encourages all actors  in a digitally enabled health ecosystem to  align their approach to digital transformation  with their UHC and SDG goals…connecting every household: The backbone infrastructure, hardware, and services required for reliable  internet access should be available, accessible, and affordable to all.  Governments, and the ICT companies they work with, should give  priority to connecting the most underserved households. National and  sub-national data on mobile and internet coverage, use, and quality  collected by governments and international organisations like the ITU  should be disaggregated to identify equity gaps between different  geographical regions, age groups, genders, etc |
| **Outcomes (type of tool or purpose i.e., Adoption or health treatment’ s domains)** | this is an adaptation framework that evaluate the health care system before the implementation of ai health rights impact assessment tool adapted to technological projects 4, Could the project affect the availability, accessibility, acceptability, and quality of other health goods and services in the country? -5. Is the project addressing priority health areas as identified in national health plans?8. Have health care workers been consulted on whether the project aligns with the national health workforce strategy? 9. Could the project affect the number of health workers available to meet primary health care obligations or obligations?10. What cadres of health workers will use the technology?12. Were departments and hospitals consulted on whether the project strengthens the present health information system? ...21.Were national or local health plans and leaders consulted before designing this technology, to ensure its alignment with plans?... 8.To ensure that state plans and strategies, health care management, and health care workers been consulted about the technology...9.To ensure that the new technology-driven project will not draw health workers away from other essential services...10.To ensure that the use of digital technology is for tasks already defined as within the scope of practice for the health worker (WHO recommendation)...12.To ensure that departments and divisions in the health system and referral hospital are consulted prior to the technology’s design...21.To ensure that the AI project is designed to further the health plan and not the profits of the AI developer... | Digital health readiness refers to the extent to which individuals and countries have the capacity to use digital technology and data for improving their own, or their population’s, health and wellbeing...A new rights- and equity-centred readiness assessment tool should be developed that policymakers and implementers in the digital health ecosystem can use to guide and influence decision-making in support of an approach to digital health that leaves no one behind, and for young people and wider civil society to use in support of advocacy and accountability efforts. |
| **SUSQI FRAMEWORK (this is the principles of the Centre of sustainable health care)** |  |  |
| **Prevention (SUSQI)Disease prevention and health promotion. All clinicians should be involved in prevention. Through broader advocacy and in individual patient care, specialties should aim to tackle underlying causes of disease – the social, economic and environmental determinants of health. Where possible, interventions should capture environmental co-benefits of healthy lifestyles, such as the improvements in air quality and carbon emissions from a shift to active travel.** | NO | yes |
| **patient empowerment and self-care (SUSQI)Patient education and empowerment. To reduce disease progression and pre-empt complications, many patients could be empowered to take on a greater role in the management of their own health and healthcare. Informed patients are also well placed to improve the coordination between clinical teams and reduce misunderstandings or duplication.** | NO | yes |
| **lean clinical pathways (SUSQI) Lean service delivery. Improving clinical decision-making in the selection and targeting of interventions will reduce lower value activities and their associated environmental impacts. Specialties can support this by describing the relevant patient pathways and providing clear, evidence-based guidance. Even where clinical input is of high value, a greater use of online records, email and telephone can reduce travel emissions by moving information in place of patients, staff and laboratory samples. Further efficiencies can follow from better integration of specialist services, such as diabetes, cardiovascular and renal care, which have a common patient base.** | YES | yes |
| **low carbon alternatives (SUSQI) Preferential use of treatment options and medical technologies with lower environmental impact. Inclusion of sustainability measures in the evaluation of medical technologies will allow service planners, clinicians and patients to choose clinically effective treatments with the best environmental profile and will encourage their further development.** | NO | yes |
| **Efficient resource use-improving use of energy, transport, water, waste and equipment.** |  |  |
| **Strengths** |  | it promotes the sustainable integration under the triple bottom line but mostly focus on social value and environmental value and the structure and policies based on human rights. It is very aligned with the SUSQI framework |
| **Weakness** | to general doesn’t have specific settings regarding to health | it is a framework with suggestions, but it is not a tool of assessment, so it is not validated, this gives a general recommendation no very specific |
| **Gaps in research** |  |  |
| **thoughts on this framework** |  |  |

| **Item** | [**[6] - Kabelo Leonard Mauco, Richard E. Scott, Maurice Mars (2021)**](https://ebooks.iospress.nl/doi/10.3233/SHTI210029) | [**[7]-Unsworth, H, et. al. (2021)**](https://www.ncbi.nlm.nih.gov/pmc/articles/PMC8236783/) |
| --- | --- | --- |
| **Citations** | [6]. Kabelo Leonard Mauco, Scott R, Mars M. Development of a Conceptual Framework for e-Health Readiness Assessment in the Context of Developing Countries [Internet]. Studies in Health Technology and Informatics. 2021 [cited 2024 Oct 7]. https://www.semanticscholar.org/paper/Development-of-a-Conceptual-Framework-for-e-Health-Mauco-Scott/72e895cae2dab5eed418e3835858d12e05552dd8 (accessed 2024 Oct 7) | [7]. Unsworth H, Dillon B, Collinson L, Powell H, Salmon M, Oladapo T, et al. The NICE Evidence Standards Framework for digital health and care technologies – Developing and maintaining an innovative evidence framework with global impact. DIGITAL HEALTH. 2021 Jan;7(24):205520762110186. https://doi.org/10.1177/20552076211018617 |
| **Title** | Development of a Conceptual Framework for e-Health Readiness Assessment in the Context of Developing Countries | The NICE Evidence Standards Framework for digital health and care technologies – Developing and maintaining an innovative evidence framework with global impact |
| **Country** | Botswana, Canada, Australia (developed framework based on Botswana) | United Kingdom |
| **Data collected** | Jan-24 | Jan-24 |
| **Year** | 2021 | 2021 |
| **Aim** | The aim of this study was to develop an e-health readiness assessment framework applicable to developing countries. | In this paper, we describe the agile policy research approach used to develop the ESF, outline how the ESF works, and describe its impact to date and ongoing work to ensure that the ESF remains up to date with the rapidly changing field of digital healthcare. |
| **Source of founding** | Telehealth Innovations in Remote Healthcare Services Delivery | Digital health |
| **Study type/ source** | article (primary and secondary data) | Article (primary data) |
| **Population** | 18 interview first stage of the framework and 15 for validation | 150 people combine with in academic HTA experts, clinicians. (The framework is for people who is responsible to 1. identifying and evaluating new DHTs, 2. conducting formal product reviews 3. authorising DHT product coverage, funding, or reimbursement 4. conducting ongoing clinical and economic product evaluations in real-world settings. |
| **Sample size** | 18 Key informants (local experts) interviewed were a director from the Botswana communications regulatory authority, three heads of district health management teams, three hospital managers, three hospital ICT managers, three community leaders, and five people with relevant experience in electronic solutions (e-solutions). rural settings (n=8), urban settings (n=10) across Botswana. | 150 experts |
| **Other demographics** | 15 e-health experts. The experts comprised six of the 18 Botswana e-health experts whose input had contributed to the development of the eHRAF, three e-health experts from low- and middle-income countries within sub-Saharan Africa (LMIC within SSA), three e-health experts from low- and middle-income countries outside sub-Saharan Africa (LMIC outside SSA), and three e-health experts from the developed world. Selecting six out the previously engaged 18 Botswana e-health experts was based on the principle that, in qualitative research, more data does not necessarily lead to more information [28] and that data collection and analysis should continue only until no new concepts emerge [29]. Hence the number of Botswana e-health experts included in this study was based on ‘data saturation’, involving iterative analysis of responses from sets of consecutively sampled groups of experts within Botswana (two per group) until no new concepts or patterns were generated. An ‘initial sample size’ of four was adopted, with the ‘stopping criterion’ being a progressive sampling of two more individuals with re-analysis until data saturation was reached [30]. International experts were purposively selected based on their e-health experience and expertise. | 95 organisations were involved including universities, industry, national institute for health research NHS trust, national association, royal colleges, department of health and social care, NHS digital, NHS improvement, private health insurers, Office for Life Sciences and Public Health England. |
| **Setting** | public health | general health |
| **Study designed** |  | In total, 13 workshops were held between June and December 2018 with representatives of the MedTech and DHT industry, health and care commissioners, academic HTA experts, clinicians and others. Over 150 people from 95 organisations, including universities, industry, National Institute for Health Research, NHS Trusts, National Associations, Royal Colleges, Department of Health and Social Care, NHS Digital, NHS Improvement, private health insurers, Office for Life Sciences and Public Health England, took part. ...Based on the literature searches and feedback from stakeholder workshops, we developed a bespoke taxonomy with 10 functional categories that are expected to cover the functions of the majority of the DHTs most frequently commissioned in the UK health and care system |
|  | inductive iterative approach to conceptualise the framework, the authors reflect on the previous cases and previous knowledge. the first framework developed use interviews and thematic analyses from 18 expert opinions. then in another study the framework was validated with 15 experts | a literature search was the first step, which search for methods that classify and evaluate digital health technology. the framework was drafted using NHS previous experience on evaluation, then it was modify repeatedly based on the workshops feedback. |
| **Concept** | the main idea of the framework is to measure the readiness of a country to integrate digital health technologies. | the approach use to develop this framework was that the quantity and quality of evidence should be proportionate to the potential clinical risk and the financial and impact of the DHT. This framework is mostly focus on clinical effectiveness, clinical safeness and acceptability ...This approach was grounded in the concept that the quality and quantity of evidence should be proportionate to the potential clinical risk and the financial and impact of the DHT. |
| **Social** | ...Various e-health readiness assessment frameworks are available, but before a framework is chosen as suitable to conduct an e-health readiness assessment in any setting, it is essential to understand the perspective and/or assumptions followed when the framework and any associated tool(s) were developed. These perspectives were the; institutional level, community level, and country level. As a result, there is little literature evidence of authors considering the influence of the government on overall e-health readiness of a country...In order to comprehensively assess e-health readiness, all stakeholders must be considered too; these include government, healthcare institutions, healthcare providers, insurers, funders, members of the public, and patients. This requires development of frameworks, and associated tools, that are relevant to each group. For example, some participants of the assessment process might not be exposed to aspects of ICT, requiring different questions asked of them. Some of the reviewed articles failed to address this issue which may result in an unreliable assessment.22 This is exemplified by Khoja et al. who developed e-health readiness assessment tools for completion by managers and healthcare providers only, but within them raised issues like political will that could only be verified by politicians themselves.11 Chipps and Mars faced challenges using this assessment tool when study participants failed to respond to some questions because they were unfamiliar with specific matters raised, or because they felt they did not have the political authority to answer the questions.22 This highlights the need for group specific e-health readiness assessment frameworks and tools.22 Thus, a tool for technical officers to assess technological readiness, would be inappropriate for healthcare providers with little or no information technology background...Culture and appropriateness of technology solutions are not considered in current frameworks. Unique social structures exist in many African countries which must be considered when developing e-health readiness assessment tools. In many developing countries there are community leaders as well as tribal leaders (chiefs) who can provide more appropriate information for assessing public readiness as they are very aware of the dynamics as well as socio-cultural factors at play in their communities. None of the tools addressed this concern. Furthermore, the issue of ‘technologically appropriate’ e-health solutions was not articulated.23–26 For example, developing world countries often face power challenges characterised by frequent power outages. A construct in a framework by Ojo et al. assessed ‘available or accessible ICT and power supply’,10 highlighting the power supply challenges characteristic of developing countries.28 It is therefore important to assess the suitability and appropriateness of the e-health technology to be implemented...Based on the study findings, a suitable e-health readiness assessment tool for Botswana would address the high level role of Government as well as the locally important roles of community and tribal leaders. It would also consider the density, distribution and literacy (general, technological and health) of the population. For example, in Botswana ICT infrastructure as well as Internet awareness and accessibility varies markedly between rural and urban areas, as well as between rural areas. In urban areas, and those rural areas closer to the only two cities in the country, ICT infrastructure is usually comparatively well established and Internet accessibility better. In contrast, communities in rural areas further away from the cities have comparatively low literacy rates, low or no Internet accessibility, and larger elderly populations, as well as individuals who may not even be aware of the Internet or wireless mobile telecommunication technologies....All relevant stakeholders need to be engaged from the inception of a national eHealth strategy to ensure that their interests are understood and addressed, including the benefits that may be delivered to each stakeholder group. They must also remain informed on progress to ensure the vision (eHealth implementation) has their continued support, and each group remains involved in the planning and delivery of the vision itself... ....and challenges with healthcare quality, accessibility and affordability.... Despite benefits associated with e-health, implementation failures unrelated to the Ehealth technology used have also been reported. Most of these failures might actually be related to a lack of e-health readiness...Within the framework, government is at the core of stakeholder engagement (Figure 1). Illustrated categories of relevant and essential stakeholders to be engaged are the private sector, community leaders, international partners, as well as nongovernmental organisations (NGOs), humanitarian organisations, and faith-based groups. The need for development of a comprehensive and informed national e-health strategy as a prerequisite to e-health readiness is shown. | …concerns: (the need for greater clarification of terms and definitions; more explanation of how the standards fit within the current regulatory compliance regime and their place in existing market access arrangements...the need to engage further with commissioners and social care, the need for guidance on the use of real-world evidence in the ESF...insufficient recognition of the widespread use of DHTs within social care; a lack of patient and public engagement; difficulties using the framework arising from the dynamic and rapidly evolving nature of digital tools and the likely crossover of technologies between evidence tiers)... |
| **Environmental** | not included | not mention in the first version but after an update in April 2022 one recommendation for stakeholders suggest including standards for environmental sustainability. |
| **Economical** | This study identified four eHealth readiness themes (governance, stakeholder issues, resources, and access), THE FOLLOWING could be resources but is systemic...However, both definitions of organizational readiness and societal readiness lacked explicit mention of interoperability as a means of attaining eHealth readiness. The issue of interoperability emerged during interviews under the subtheme of institutional governance. One key informant stated, “We currently have so many systems in place, we need to find out if they are able to speak to each other and if there is a backup system.” Interoperability has been defined as the extent to which systems and devices can exchange data and interpret the shared data [Healthcare Information Management Systems Society. 2013. What is interoperability? URL: https://www.himss.org/library/interoperability-standards/what-is-interoperability [accessed 2019-07-10] [WebCite Cache]18]. Most developing countries including Botswana are, or have been, recipients of eHealth systems from foreign donors and international partnerships. This results in the presence of a number of systems that are unable to communicate with each other. Hence, interoperability is an issue that needs to be addressed in any eHealth readiness assessment framework meant for developing countries. The importance of interoperability can be estimated by the fact that it is specifically mentioned in the WHO and ITU National eHealth Strategy Toolkit as one of the eHealth components to be addressed in the development of a national eHealth vision [World Health Organization, International Telecommunications Union. National eHealth strategy toolkit. Geneva: WHO Press; 2012:1-37.17]....One expert suggested that e-commerce be removed from the framework. e-Commerce refers to the use of ICTs to conduct business transactions among buyers, sellers, and other trading partners, as well as mechanisms for reimbursement of healthcare providers for services provided. No matter the country, healthcare incurs ‘costs’ typically paid for through one or more of government, insurers, NGOs, or consumers. To patients or consumers, the costs are usually what they pay out of pocket for healthcare services or insurance premiums. To healthcare providers (healthcare organisations or clinicians) the costs relate to expenses they incur in delivering services (and can include items such as equipment, depreciation, personnel, and overhead) [31]. Literature has documented e-commerce as a critical and integral component of e-health [32,33,34] and should be a component of eHRAF...ll. The intricacy and opportunity costs of e-health implementations, and their ongoing maintenance and sustainability costs, cannot be disregarded. Ensuring e-health readiness prior to a healthcare facility or country committing to e-health implementations is critical and requires real-life application of the eHRAF... | ...concerns:( a perceived lack of capacity and capability in the system for economic analysis; greater attention needed on the impact of real-world data and real-world evidence...requesting additional resources to help users understand evidence generation and health economic analyses, a perceived lack of clarity about the remit of the ESF and how it fitted with other initiatives including the NHS Digital DAQ and 2017 EU Medical Device Regulation (MDR) for software)...The evidence for economic impact standards provide information on the key considerations required for undertaking and reporting economic evaluations of DHTs, as well as categorising the level of financial ‘risk’ into 3 levels: basic, low and high. Typical examples of these 3 levels are, for basic: a pilot project at a local level; for low: a regional initiative or a potentially cost-saving national initiative; and for high: a national commissioning decision likely to be cost-incurring, for example, through service redesign. The type of economic analysis specified in the standards is related to the level of financial risk identified ... |
| **Measurements** | | |
| **Social** | Government readiness: Gauges the extent to which a country’s government and politicians support and promote awareness, implementation, and use of eHealth innovations (eg, presence of relevant policies and funding) .... Societal readiness: Gauges the degree of “interaction” associated with a health care institution. Interaction is described by three parameters: interaction among members of a health care institution, interaction of a health care institution with other health care institutions, and interaction of a health care institution with its local communities...Health care provider readiness: Gauges the influence of a health care provider’s personal experience, primarily their perception and receptiveness toward the use of eHealth technology....Engagement readiness: Gauges the extent to which members of a community are exposed to the concept of eHealth and are actively debating its perceived benefits as well as negative impacts. It also involves gauging the willingness of members of a community to accept training on eHealth....Core readiness: Gauges the extent to which members of a community are dissatisfied with the current status of their health care service provision, see eHealth as a solution, and express their need and preparedness for eHealth services....Public-patient readiness: Gauges the extent to which members of the public and patients are aware of, and can afford and access, eHealth services. It also involves gauging the influence of their personal experiences on their perception and receptiveness toward the use of eHealth technology.... Governance captured various subthemes that the key informants believed needed consideration at both national and institutional levels to ensure eHealth readiness. Stakeholder issues encapsulated subthemes concerned with ensuring that community members were involved during implementation of eHealth projects. Resources identified human, structural, and budgetary subthemes. Access comprised several subthemes concerned with ensuring all community members (eg, citizens and health care workers) were able to access eHealth services...DOMAINS IN THE INTERVIEWS: Organizational readiness: Gauges the extent to which the institutional setting and culture supports and promotes awareness, implementation, and use of eHealth innovations (eg, presence of relevant policies and senior management support)....This study also identified lack of literacy as an issue, with a participant noting, “Another challenge is that of education level. If you go to villages, you will find a lot of people that are illiterate and not sensitized to the benefits of electronic communications.” Lack of basic literacy, technical literacy, and health literacy, as highlighted during the interviews, can also contribute to denying the populace access to eHealth services. Measures of such types of literacy also need to be incorporated into any eHealth readiness assessment framework and tool. This is associated with the need to ensure that eHealth resources can be accessed in local languages...domains : Organizational readiness- Gauges the extent to which the institutional setting and culture supports and promotes awareness, implementation, and use of e-health innovations (e.g., presence of relevant policies; senior management support) health care providers readiness: Gauges the influence of a healthcare provider’s personal experience; primarily their perception and receptiveness towards the use of e-health technology. engagement readiness: Gauges the extent to which members of a community are exposed to the concept of e-health and are actively debating its perceived benefits as well as negative impacts. It also involves gauging the willingness of members of a community to accept training on e-health.... societal readiness : Gauges the degree of ‘interaction’ associated with a healthcare institution. Interaction is described by three parameters; interaction among members of a healthcare institution, interaction of a healthcare institution with other healthcare institutions, and interaction of a healthcare institution with its local communities. Core readiness: Gauges the extent to which members of a community are dissatisfied with the current status of their healthcare service provision, see e-health as a solution, and express their need and preparedness for e-health services. Public/Patient readiness: Gauges the extent to which members of the public and patients are aware of, and can afford and access, e-health services. It also involves gauging the influence of their personal experiences on their perception and receptiveness towards the use of e-health technology...Two aspects are then identified as essential factors in determining an e-health ready setting, both of which require specific assessment at different points in time. First, the presence of stakeholder engagement, ideally addressed early in the process, i.e., from inception. | Acceptability with users: Be able to show that representatives from intended user groups were involved in the design, development or testing of the DHT. Provide data to show user satisfaction with the DHT...Published or publicly available evidence to show that representatives from intended user groups were involved in the design, development or testing of the DHT and to show that users are satisfied with the DHT...Equalities considerations: Evidence, if relevant, that the DHT: Contributes to challenging health inequalities in the UK health and social care system, or improving access to care among hard-to-reach populations. Contribute to promoting equality, eliminating unlawful discrimination and fostering good relations between people with protected characteristics (as described in the 2010 Equalities Act) and others, Show evidence of the DHT being used in hard-to-reach populations, or that its use reduces health inequalities... TIER B: Quality and safeguarding: Show that appropriate safeguarding measures are in place around peer-support and other communication functions within the platform. Describe: who has access to the platform and their roles within the platform, why these people or groups are suitable and qualified to have access, any measures in place to ensure safety in peer-to-peer communication, for example through user agreements or moderation. As for the minimum evidence standard... Acceptability with users: Be able to show that representatives from intended user groups were involved in the design, development or testing of the DHT. Provide data to show user satisfaction with the DHT. Published or publicly available evidence to show that representatives from intended user groups were involved in the design, development or testing of the DHT and to show that users are satisfied with the DHT...Equalities considerations: Evidence, if relevant, that the DHT:  Contributes to challenging health inequalities in the UK health and social care system, or improving access to care among hard-to-reach populations. Contribute to promoting equality, eliminating unlawful discrimination and fostering good relations between people with protected characteristics (as described in the 2010 Equalities Act) and others. Show evidence of the DHT being used in hard-to-reach populations, or that its use reduces health inequalities. access acceptability and clinical safe |
| **Environmental** | no features on this | Standard 3: consider environmental sustainability  Applies to DHTs in tiers A, B and C.  Information that can be used to meet standard 3  The NHS has set ambitions to have a net zero carbon footprint by 2040. Environmental  sustainability should be factored into all stages of the life cycle of the DHT starting at the  design of the DHT. The company should provide a narrative description of any expected  environmental sustainability benefits and negative impacts from using the DHT. This  should focus on impacts on greenhouse gas emissions, in line with the NHS carbon  footprint and carbon footprint plus. |
| **Economical** | DOMAINS IN THE INTERVIEW: Technological-infrastructural readiness: Gauges the availability and affordability of ICT resources necessary to implement a proposed eHealth innovation (eg, skilled human resources, ICT support, quality ICT infrastructure, and power supply)...Government readiness: Gauges the extent to which a country’s government and politicians support and promote awareness, implementation, and use of eHealth innovations (eg, presence of relevant policies and funding).. The definition seems to be more concerned with ICT resources and does not adequately address the need for other resources such as a budget specific for eHealth, ICT infostructure, and the relevant human resources. A specific budget for eHealth is crucial for sustainability of a project and must be determined as part of the business plan prior to embarking on eHealth implementation. Equally important is the availability of sufficient and appropriate human health resources, or more specifically, human eHealth resources (i.e., professionals knowledgeable and trained in eHealth). Infostructure is an ill-defined term but has been considered as all needs beyond physical hardware and software infrastructure. Despite its ephemeral nature, it is an important inclusion as a factor determining readiness.... domains - Technological/Infrastructural readiness: Gauges the availability and affordability of ICT resources necessary to implement a proposed e-health innovation (e.g., skilled human resources, ICT support, quality ICT infrastructure, and power supply. Government readiness: Gauges the extent to which a country’s Government and politicians support and promote awareness, implementation, and use of e-health innovations (e.g., presence of relevant policies, and funding) in organisational level it is mentioned how the institution will support the innovation of digital health interventions. in the technological an infrastructure it is mentioned the affordability of supporting ehealth... Second, the presence of relevant e-health infostructure and infrastructure, ideally addressed later in the process once specific e-health solutions have been identified. | The evidence for economic impact standards provides information on the key considerations required for undertaking and reporting economic evaluations of DHTs, as well as categorising the level of financial ‘risk’ into 3 levels: basic, low and high. Typical examples of these 3 levels are, for basic: a pilot project at a local level; for low: a regional initiative or a potentially cost-saving national initiative; and for high: a national commissioning decision likely to be cost-incurring, for example, through service redesign. The type of economic analysis specified in the standards is related to the level of financial risk identified...CONTEXTUAL QUESTIONS: Is the financial or organisational risk of the DHT expected to be very high? DHTs with very high financial risk should be assessed using the best practice standards to provide surety that the DHT represents good value. High organisational risks may include situations in which implementing the DHT would need complex changes in working practice or care pathways...EVIDENCE FOR ECONOMIC IMPACT STANDARDS: Basic: Budget impact analysis. Estimated yearly budget impact for years 1 to 2. Data may be collected to inform future economic analyses...Low financial commitment. Cost–consequence analysis. Estimated costs and benefits. Sensitivity analysis results. Budget impact analysis. Estimated yearly budget impact for years 1 to 5. Sensitivity analysis results. High financial commitment. For DHTs with health outcomes funded by the NHS and Personal Social Services, a cost–utility analysis should be done using NICE's guide to the methods of technology appraisal as a reference case. Estimated incremental cost–effectiveness ratio. Sensitivity analysis results. For DHTs funded by the public sector with health and non-health outcomes, or for DHTs that focus on social care, a cost–utility analysis should be done. If this is not possible, a cost–consequence analysis may be acceptable. The analysis should be done using developing NICE guidelines: the manual as a reference case. Estimated incremental cost–effectiveness ratio (cost–utility analysis) or estimated costs and benefits (cost–consequence analysis). Sensitivity analysis results. Budget impact analysis. Estimated yearly budget impact for years 1 to 5. Sensitivity analysis results. |
| **Outcomes (type of tool or purpose i.e., Adoption or health treatment’ s domains)** | ...Botswana, like many developing countries, must now contend with communicable diseases, and the increase in burden of ‘Western’ non-communicable diseases (e.g. diabetes, heart disease, obesity). In Botswana both morbidity and mortality for all ages are still dominated by infectious diseases, with HIV/AIDS and other communicable diseases causing about half of all deaths.29 The infant mortality and under five mortality rates remain high with year on year fluctuations,29 with more than two-thirds of these deaths due to communicable diseases: diarrhoea and pneumonia being the two main contributors. Life expectancy in Botswana is estimated at 54.4 years (48.8 males and 60 females), and there is a skewed distribution of wealth in Botswana,29 with 23.1% of the total population living on less than US $ 1.25 per day...tool for assess readiness of ehealth in a developing country | ...concerns: (refinement of the risk concepts and the approach to artificial intelligence. Following this feedback, minor changes were made to the framework to provide additional clarity and specificity...a need for greater clarity in the functional classification system and evidence requirements, requesting information on the role of users in testing and signing off new DHTs...questions about the process of how the ESF will be used in practice)...the outcome is a framework to support local and national purchasing decisions around DHTs in England, and to help developers of DHTs to plan the generation of their evidence base. MEASUREMENTS TIER A : (Credibility with UK health and social care professionals: Be able to show that the DHT has a plausible mode of action that is viewed as useful and relevant by professional experts or expert groups in the relevant field. Either: show that relevant clinical or social care professionals working within the UK health and social care system have been involved in the design, development or testing of the DHT, or show that relevant clinical or social care professionals working within the UK health and social care system have been involved in signing-off the DHT, indicating their informed approval of the DHT...Published or publicly available evidence documenting that the DHT has a plausible mode of action that is viewed as useful and relevant by professional experts or expert groups in the relevant field. Either: show that relevant clinical or social care professionals working within the UK health and social care system have been involved in the design, development or testing of the DHT, or show that relevant clinical or social care professionals working within the UK health and social care system have been involved in signing-off the DHT, indicating their informed approval of the DHT...Relevance to current care pathways in the UK health and social care system: Evidence to show that the DHT has been successfully piloted in the UK health and social care system, showing that it is relevant to current care pathways and service provision in the UK. Also, evidence that the DHT can perform its intended function to the scale needed (for example, having servers that can scale to manage the expected number of users)-Evidence to show successful implementation of the DHT in the UK health and social care system)...TIER A(Accurate and reliable measurements: Data or analysis which shows that the data generated or recorded by the DHT is: accurate, reproducible, relevant to the range of values expected in the target population. Also data showing that the DHT is able to detect clinically relevant changes or responses. As for the minimum evidence standard, but with quantitative data...Accurate and reliable transmission of data: Technical data showing that numerical, text, audio, image-based, graphic-based or video information is: not changed during the transmission process, not biased by the data ‘value’ expected from the target patient population. As for the minimum evidence standard, but with quantitative data.). TIER B MEASUREMENTS( Reliable information content: Be able to show that any health information provided by the DHT is:  valid (aligned to best available sources, such as NICE guidance, relevant professional organisations or recognised UK patient organisations, and appropriate for the target population), accurate, up to date, reviewed and updated by relevant experts at defined intervals, such as every year, sufficiently comprehensive. Evidence of endorsement, accreditation or recommendation by NICE, NHS England, a relevant professional body or recognised UK patient organisation. Alternatively, evidence that the information content has been validated though an independent accreditation...Ongoing data collection to show usage of the DHT: Commitment to ongoing data collection to show usage of the DHT in the target population, and commitment to share, when available, with relevant decision-makers such as commissioners in a clear and useful format. Evidence that data on usage is being collected in line with the minimum standards and can be made available to relevant decision-makers...Ongoing data collection to show value of the DHT: Commitment to ongoing data collection to show user outcomes (if relevant) or user satisfaction (using non-patient identifiable information) to show ongoing value, and commitment to share, when available, with relevant decision-makers such as commissioners in a clear and useful format. Evidence that data on outcomes or user satisfaction is being collected in line with the minimum standard and can be made available to relevant decision-makers...Credibility with UK health and social care professionals: Be able to show that the DHT has a plausible mode of action that is viewed as useful and relevant by professional experts or expert groups in the relevant field. Either:  show that relevant clinical or social care professionals working within the UK health and social care system have been involved in the design, development or testing of the DHT, or show that relevant clinical or social care professionals working within the UK health and social care system have been involved in signing-off the DHT, indicating their informed approval of the DHT. Published or publicly available evidence documenting that the DHT has a plausible mode of action that is viewed as useful and relevant by professional experts or expert groups in the relevant field. Either: show that relevant clinical or social care professionals working within the UK health and social care system have been involved in the design, development or testing of the DHT, or show that relevant clinical or social care professionals working within the UK health and social care system have been involved in signing-off the DHT, indicating their informed approval of the DHT...Relevance to current care pathways in the UK health and social care system: Evidence to show that the DHT has been successfully piloted in the UK health and social care system, showing that it is relevant to current care pathways and service provision in the UK. Also, evidence that the DHT can perform its intended function to the scale needed (e.g., having servers that can scale to manage the expected number of users). Evidence to show successful implementation of the DHT in the UK health and social care system. MEASURMENTS TIER C: Demonstrating effectiveness – for preventative behaviour change or self-manage functions: High quality observational or quasi-experimental studies demonstrating relevant outcomes. These studies should present comparative data. Comparisons could include relevant outcomes in a control group, use of historical controls, routinely collected data. Relevant outcomes may include:  behavioural or condition-related user outcomes such as reduction in smoking or improvement in condition management, evidence of positive behaviour change, user satisfaction. High quality intervention study (quasi-experimental or experimental design) which incorporates a comparison group, showing improvements in relevant outcomes, such as:  patient-reported outcomes (preferably using validated tools) including symptom severity or quality of life, other clinical measures of disease severity or disability, healthy behaviours,  physiological measures, user satisfaction and engagement, health and social care resource use, such as admissions or appointments. The comparator should be a care option that is reflective of standard care in the current care pathway, such as a commonly used active intervention. ...Demonstrating effectiveness for Treat, Active monitoring, Calculate or Diagnose functions: High quality intervention study (experimental or quasi-experimental design) showing improvements in relevant outcomes, such as:  diagnostic accuracy, patient-reported outcomes (preferably using validated tools), including symptom severity or quality of life, other clinical measures of disease severity or disability, healthy behaviours, physiological measures, user satisfaction and engagement.  Generic outcome measures may also be useful when reported alongside condition-specific outcomes. The comparator should be a care option that is reflective of the current care pathway, such as a commonly used active intervention...High quality randomised controlled study or studies done in a setting relevant to the UK health and social care system, comparing the DHT with a relevant comparator and demonstrating consistent benefit including in clinical outcomes in the target population, using validated condition-specific outcome measures. Alternatively, a well-conducted meta-analysis of randomised controlled studies if there are enough available studies on the DHT...Use of appropriate behaviour change techniques: Be able to show that the techniques used in the DHT are: consistent with recognised behaviour change theory and recommended practice (aligned to guidance from NICE or relevant professional organisations) appropriate for the target population. Published qualitative or quantitative evidence showing that the techniques used in the DHT are: based on published and recognised effective behaviour change techniques, aligned with recommended practice, appropriate for the target population...Reliable information content: Be able to show that any health information provided by the DHT is:  valid (aligned to best available sources, such as NICE guidance, relevant professional organisations or recognised UK patient organisations, and appropriate for the target population),  accurate, up to date, reviewed and updated by relevant experts at defined intervals, such as every year, sufficiently comprehensive. Evidence of endorsement, accreditation or recommendation by NICE, NHS England, a relevant professional body or recognised UK patient organisation. Alternatively, evidence that the information content has been validated though an independent accreditation such as The Information Standard or HONcode certification...CONTEXTUAL QUESITONS: Are the intended users of the DHT considered to be in a potentially vulnerable group such as children or at-risk adults?: NHS England defines an at-risk adult as an adult ‘who may be in need of community care services by reason of mental or other disability, age or illness; and who is or may be unable to take care of him or herself, or unable to protect him or herself against significant harm or exploitation.’ If the DHT is intended to be used by people considered to be in a potentially vulnerable group then a higher level of evidence may be needed, or relevant expert opinion on whether the needs of the users are being appropriately addressed...How serious could the consequences be to the user if the DHT failed to perform as described?: A higher level of potential harm may indicate that the best practice evidence standards should be used...Is the DHT intended to be used with regular support from a suitably qualified and experienced health or social care professional?: DHTs that are intended to be used with support (that is, with regular support or guidance from a suitably qualified and experienced health or social care professional) could be considered to have lower risk than DHTs that are intended to be used by the patient on their own. This contextual question may require careful interpretation depending on the individual DHT as the involvement of a clinician may in itself indicate that the DHT presents a specific risk...Does the DHT include machine learning algorithms or artificial intelligence? Refer to the code of conduct for data-driven health and care technology for additional considerations when assessing DHTs that use artificial intelligence or machine learning... |
| **SUSQI FRAMEWORK (this is the principles of the Centre of sustainable health care)** |  |  |
| **Prevention (SUSQI)Disease prevention and health promotion. All clinicians should be involved in prevention. Through broader advocacy and in individual patient care, specialties should aim to tackle underlying causes of disease – the social, economic and environmental determinants of health. Where possible, interventions should capture environmental co-benefits of healthy lifestyles, such as the improvements in air quality and carbon emissions from a shift to active travel.** | no | no |
| **patient empowerment and self-care (SUSQI)Patient education and empowerment. To reduce disease progression and pre-empt complications, many patients could be empowered to take on a greater role in the management of their own health and healthcare. Informed patients are also well placed to improve the coordination between clinical teams and reduce misunderstandings or duplication.** | yes | yes |
| **lean clinical pathways (SUSQI) Lean service delivery. Improving clinical decision-making in the selection and targeting of interventions will reduce lower value activities and their associated environmental impacts. Specialties can support this by describing the relevant patient pathways and providing clear, evidence-based guidance. Even where clinical input is of high value, a greater use of online records, email and telephone can reduce travel emissions by moving information in place of patients, staff and laboratory samples. Further efficiencies can follow from better integration of specialist services, such as diabetes, cardiovascular and renal care, which have a common patient base.** | no | yes |
| **low carbon alternatives (SUSQI) Preferential use of treatment options and medical technologies with lower environmental impact. Inclusion of sustainability measures in the evaluation of medical technologies will allow service planners, clinicians and patients to choose clinically effective treatments with the best environmental profile and will encourage their further development.** | no | yes |
| **Efficient resource use-improving use of energy, transport, water, waste and equipment.** |  |  |
| **Strengths** | it is validated | validation and wide population The ESF page on the NICE website has been viewed over 55,000 times since publication in December 2018 and downloaded over 19,000 times. The most downloaded of the supporting documents are the user guide (over 2500 downloads), the budget impact template (over 1500 downloads) and the functional classification case studies (over 1400 downloads). Several healthcare systems, academic groups and commercial organisations outside of the UK have expressed interest in the NICE ESF. These include academic groups, national evaluators or governmental bodies from India, Norway, Indonesia, The Republic of Korea, Denmark, the Netherlands, and Sweden. The ESF has been cited in over 50 academic publications. The descriptive paper explaining the purpose of the ESF 10 has been cited 35 times. The accelerator groups that were involved in the co-design of the ESF use the standards routinely to advise innovators on evidence generation. The ESF is now being used as the standard by innovators seeking to access funding from the UK’s major research and development funders such as NIHR i4i and Innovate UK SME. |
| **Weakness** | This illustrates that the use of a single generic tool for e-health readiness assessment within any framework has the potential to negatively affect the validity of such an assessment. Indeed, the same sentiment was expressed by Khoja et al. who in their study developed separate e-health readiness assessment tools for managers and healthcare providers, so that e-health readiness could be determined from both perspectives ...e-Health consists of various components including health informatics, telehealth, ecommerce, as well as technology enabled learning [2]. As previously discussed, to assesse-health readiness comprehensively the various components must all be taken intoconsideration [7]. Furthermore, readiness for one component does not necessarilytranslate to an overall e-health readiness, or readiness for any other specific component. | mention it in the text: Another limitation is that our main focus was on health settings, but we recognise the extensive and increasing use of DHTs in social care...It is anticipated that as consensus develops on the HTA of DHTs that use adaptive AI, the ESF would be extended to include these. Recent work by the British Standards Institute (BSI) and the Association for the advancement of Medical Instrumentation (AAMI) has set out recommendations for AI in medical devices.25,26 NHSX’s AI lab 27 has been created to address the challenges of safe and ethical adoption of AI-based DHTs within the healthcare system and has a number of workstreams to address these challenges. NHSX, NICE, CQC and MHRA will be jointly working on developing a ‘joined-up’ regulatory and approval system 28 for AI-based DHTs in the health and care system. This work will be providing a useful means for pulling together the currently fragmented system for regulating and evaluating AI-based DHTs, into a coherent process for the UK health and care system.... A further (and related) limitation of this work is that it conceptualises DHTs as standalone tools which provide specific functions, rather than as part of services or pathways. Future developments in this area are likely to see DHTs become harder to separate as single entities for evaluation, as they become more integrated within clinical pathways or more embedded within health consumer’s own digital ecosystems as providers such as Apple and Google incorporate health functions across a range of devices and consumer facing services. The ESF was designed to be used for appraising evidence for DHTs being commissioned in the UK health and care system and it is less relevant to technologies that are available directly to public users, such as through app stores |
| **Gaps in research** |  |  |
| **thoughts on this framework** |  |  |

| **Item** | [**[8]- WHO GUIDELINES YOUTH (2021)**](https://www.who.int/publications/i/item/9789240011717) | [**[9] DIIG Digital implementation investment guide (2020)**](https://iris.who.int/bitstream/handle/10665/334306/9789240010567-eng.pdf?sequence=1&isAllowed=y) |
| --- | --- | --- |
| **Citations** | [8]. World health organisation. Youth-centred digital health interventions: a framework for planning, developing and implementing solutions with and for young people [Internet]. www.who.int. 2021 [cited 2024 Feb 2]. https://www.who.int/publications/i/item/9789240011717 (accessed 2024 Feb 2) | [9]. World health organisation. Digital Implementation Investment Guide (DIIG): Integrating Digital Interventions into Health Programmes [Internet]. www.who.int. 2020. https://www.who.int/publications/i/item/9789240010567 |
| **Title** | Youth-centred digital health interventions: a framework for planning, developing and implementing solutions with and for young people. | Digital implementation investment guide (DIIG): integrating digital interventions into health programmes |
| **Country** | Geneva WHO (worldwide) | GENEVEWHO, PATH, UNFPA, UNICEF, HRP |
| **Data collected** | Jan-24 | Jan-24 |
| **Year** | 2020 | 2020 |
| **Aim** | This is a guidance on planning, developing and implementing digital  interventions to promote better health among adolescents and youth. | The Guide is designed to walk users of the document step-by-step through planning, costing and implementing digital health interventions within a digital health enterprise. This consists of selecting digital health interventions that are aligned with identified health needs, appropriate to a specific country context and integrated with existing technologies and the broader digital architecture. Users of the Guide will learn from diverse experiences deploying digital health technologies over the past decade and will be guided through a systematic  approach to designing, costing and implementing meaningful digital health interventions that are part of a digital health enterprise. |
| **Source of founding** | WHO- IRIS (Institutional repository for information sharing) | WHO WEB PAGE IRIS |
| **Study type/ source** | Government Guidelines | White paper |
| **Population** | Youth | NO POULATION LITERATURE REVIEW |
| **Sample size** |  |  |
| **Other demographics** | The next step was to interview global experts from academia, nongovernmental organizations (NGOs),  research institutes, social enterprises and UN agencies about designing digital health interventions for  young people. At a subsequent three-day workshop co-hosted by WHO and UNICEF, these and other  experts reviewed, vetted and discussed each area of the evolving framework and shared key lessons  from their experiences in the field. The experts subsequently provided additional input to help refine  the framework and this document. | Based on technologies mean to promote and prevent health |
| **Setting** | General health for youth | public health |
| **Study designed** |  |  |
|  | that included a  targeted literature review, consultations, interviews, a workshop and additional input from experts and  young people |  |
| **Concept** | this framework is based on the following 3 principles iteration, equity and sustainability |  |
| **Social** | ...Engaging adolescents and youth means actively and intentionally involving them as co-creators, collaborators, problem solvers, champions and change agents. Meaningful engagement requires an inclusive and mutually respectful partnership between young people and adults in which power is shared, respective contributions are valued and acknowledged, and young people’s ideas, perspectives, skills and strengths are integrated into the design and delivery of intervention...As a result, young people are not just the beneficiaries of the intervention but also drivers of change, especially when it comes to their own health (33). They are the experts on what health information young people need and what technology young people are using. Engaging them meaningfully, as part of the project team, requires careful planning as well as monitoring and evaluation (M&E) throughout the planning, development and implementation stages of the digital health intervention. This is mutually beneficial for them and for the developers of the intervention: the young people have an opportunity to contribute their ideas and experiences to the development of an intervention they can use, and the developers gain a better understanding of the population they are trying to reach and can create a better product that is more likely to succeed. For many organizations, engaging young people in this way can be challenging, so they vary in how much they involve young people in the process. It can range from having several young people engaged in every aspect of planning, development and implementation to having a youth advisory board to consult throughout the process. Annex 2 offers some do’s and don’ts for engaging young people. Half-hearted efforts should be avoided at all costs, such as those in which young people are seemingly given a voice but in fact have little or no say in the content or the style of communicating it and little or no decision-making authority (34). Tokenism should also be avoided, such as inviting one young person to a meeting to represent “the voices of young people” or having young people participate in consultations but not following up with them. Approaching youth engagement in these half-hearted ways is generally unsuccessful and can be harmful to the young people involved, including making them feel disrespected and used...Involving young people at every phase of the development process helps ensure that the intervention will be valued and sustainable. To ensure authentic engagement and create a safe space for young people, Youth Development Labs (YLabs) recommends emphasizing two areas: safety and fun. Safety. Creating an environment that is safe and feels safe is critical. YLabs works to ensure this by developing, practicing and implementing “safeguarding protocols.” For example, one safeguarding protocol is beginning each session by explaining that participation is always voluntary. Because of the sensitive nature of the topics and the power dynamics between the adult developers and young participants, it is important to let the participants know that there are no right or wrong answers, they can decline to answer any question and they can stop the session if they feel uncomfortable. Another example of a safeguarding protocol is working with local implementing partners to provide access to counsellors and resources after the sessions, in case participants need additional health information or support. Fun. YLabs begins workshops with warm-up games, drawing and other activities that help young participants relax. Young people are often bored by long presentations, so YLabs uses activities that include more “showing” activities to keep young people engaged. Role-playing games are particularly useful because they provide young people with an  energizing and enjoyable opportunity to share their own stories and provide the development team with rich data and nuances. Other engaging activities include mapping and card sorting, which are helpful for understanding how young people relate to their friends, family and community. Bold colours, visual designs, costumes and music can help make the session feel more like a party than a formal interview...Involve the right people at the right time Each part of the process requires different expertise, input and buy-in. Involving the right people at the right time means knowing who should be at the table, for what purpose and at what point in the process. Some people or organizations may be involved throughout the entire process, but at different levels. The core team members should know their own roles and identify the expertise they will need at particular times—for example, expertise in digital technologies or promotion and marketing. Team members should perform only the tasks they are trained for and capable of doing, while allowing others on the team or other participants to contribute their skills when and where needed. The core team involved throughout the whole process may include young people, a project manager, a financial manager, researchers, behavioural and data scientists, health content-area experts, digital health specialists, implementing partners (such as local organizations or NGOs), M&E analysts, representatives from funding organizations, and representatives from local and national government (including the ministry of health, ministry of information and communications technology and possibly the ministry of finance). Some team members may have the experience and affiliation to fulfil more than one role. All team members who work with young people should have skills and training in providing a safe and engaging experience for young collaborators. Additional participants who should be kept informed and consulted throughout the process may include telecommunications partners, mobile network operators, recruiting partners (such as schools or local youth networks), local policymakers, civil society organizations, marketing organizations and significant figures for young people (including parents, schools, educators, clinicians and youth influencers)...Make decisions based on data and evidence Every activity and decision should be supported by quantitative and qualitative data and evidence, which will serve as a check on intuition and ensure that the intervention is aligned with actual user needs and preferences. Each stage requires collecting data and  evidence and factoring it into the decision-making process...Promote safety, privacy and ethical standards Young collaborators and future users should be protected from potential abuse, incompetence and violations of privacy (35). The information and data they provide should be kept confidential, and they should be able to decide how much information they want to reveal and to whom (35). The following are key considerations for ensuring that young people are kept safe, their privacy is respected and ethical standards are followed: Use appropriate terminology when describing young people. A range of terminology applies to adolescents—for example, child, minor, juvenile, teenager, youth and ward. Although these terms may seem similar and may overlap in meaning, they have specific legal, social, cultural and health connotations and implications. Their definitions also vary within and between countries, within and between regions, and even at the international level because of different social and cultural assumptions (36). Establish processes for obtaining informed consent for young people’s participation, particularly for those under age 18 (36). Married young people under age 18 are often considered emancipated and legally capable of giving consent; unmarried people under age 18 may assent to participate but still need formal consent from a parent or guardian (37). Resources Youth-centred digital health interventions. Respect, protect and ensure young people’s right to privacy and their right to share, access and receive information. In some cases, the personal information or stories that young people disclose may need to be reported to relevant parties as an ethical or legal obligation, even though it may jeopardize the trust being built with them. For example, a young person may have a serious illness that requires medical intervention, be at risk of harming themselves or others, or be at risk of neglect or abuse (36). The specifics of keeping young people safe, ensuring their privacy and maintaining ethical standards will depend on the delivery channel of the intervention, the context in which the intervention is being implemented and the laws and regulations of the country. This will affect what data are collected; how data are collected, used, stored and shared; and how confidential information and the identities of young people are protected. However, it should be standard practice to acknowledge the sensitivity of the data being collected, to be transparent about how the data will be collected and used, and to develop, adopt and enforce security policies to protect the data...Digital health interventions should not be standalone solutions; rather, they should complement and strategically integrate with existing health interventions. Therefore, it is crucial to understand not only young people’s actual needs but also the implementing environment, what already exists and how a potential digital health intervention can enhance current efforts. A landscape analysis and a needs assessment can provide a deeper understanding of and objective information about the local context, the target audience, and implementing constraints and opportunities. A landscape analysis gathers information on the mobile technology infrastructure, existing relevant digital and nondigital health interventions, and relevant organizations and community groups working to improve the health of young people. A needs assessment determines who needs the intervention, how great their need is and what activities will best address those needs (25). Both activities should be revisited routinely to understand the changing digital and health landscapes and users’ needs and as a way to collect data for continuous improvement of the intervention...Enlist research partners to systematically undertake the steps required for a landscape analysis and a needs  assessment...Plan enough time for these activities, especially formative research, which may require ethical approvals from institutional review boards and may involve identifying young people and others to interview and consult. Communicate the timeline to funders early in the process...Engage not only young people but also key community gatekeepers such as community leaders, parents and teachers to determine the acceptability of the intended intervention...Clearly document the steps taken during the landscape  analysis and the needs assessment; this will be helpful when developing the intervention...Look ahead to implementation by identifying successful existing promotion and marketing efforts for health and non-health-related content that young people are already sharing. Build on what they are doing and find ways to collaborate...Ensure ethical research practices, including appropriate training in human subjects research, appropriate procedures for research staff, and robust assent/consent processes and data protection measures...Ensure that the mapping of the theory-driven approach is a consultative process that includes facilitated workshops with relevant parties, including young people. Involving stakeholders in a workshop to develop the theory-driven approach can provide additional and deeper insight. This may also improve buy-in and engagement from stakeholders who could be collaborators for implementation...The importance of youth-driven workshops The digital communication channels most favoured by young people are always changing and may differ by location. When YTH (Youth Tech Health), an initiative of ETR, was developing an intervention called ZonaSegura to address dating violence among Honduran teens (in partnership with the Public Health Institute’s GOJoven Honduras), the initial plan was to create a mobile application for girls and an SMS intervention for all young people. YTH representatives visited Honduras on two occasions to hold workshops with about 35 teens (aged 14–19). Within the first minutes of the first workshop, the teens reported never using or receiving SMS messages; they communicated instead via WhatsApp—the most popular platform among the intervention’s target audience. This vital information redirected plans for the delivery mechanism for ZonaSegura. YTH’s previous messaging interventions used SMS, but these projects were primarily located in the United States. YTH invited young people to help generate ideas during the second workshop. Participants were asked how they would talk to their friends about healthy relationships and toxic masculinity and what kinds of messages their friends would want to receive in Instagram- or Pinterest-like inspirational quotes. After they were shown examples, participants were given 10 minutes to brainstorm ideas and write messages on sticky notes, many of which were directly incorporated into ZonaSegura. YTH provided the following tips for creating a youth-friendly environment for focus groups and co-design workshops: » Create a welcoming environment. In the United  States, for example, YTH provides familiar and interactive objects and materials such as fidget spinners, playdough and pipe cleaners. » A welcoming environment is not the same in all cultures. During workshops in Honduras, the YTH team learned that it was considered rude to not offer coffee. Organizations should rely on in-country partners or contacts to gain these kinds of insights and nuanced information ahead of time. Tailoring the environment to the participants and adhering to common cultural practices can have a positive impact on the outcome of workshops.» Hold sessions in familiar spaces. Young people are likely to be more engaged in a classroom or cafe than in a hotel conference room...Co-designing with more than young people One of YLabs’ flagship projects is CyberRwanda, a digital platform that aims to improve the health of peri-urban and urban adolescents (aged 12–19) in Rwanda and increase access to high-quality, youth-friendly services and contraceptive methods. While contraceptives are free or available through insurance in Rwandan health clinics, many young people prefer to not use those clinics because they lack privacy and young people perceive that they are a low priority for providers there. The YLabs team found that young people turned instead to neighbourhood pharmacies that offered quick, private access to contraceptive methods and health products. This important insight led YLabs to hold co-design workshops with pharmacists to learn about their attitudes toward young customers, understand the challenges pharmacies faced in fulfilling orders, and learn what would motivate them to use the digital platform. By working with the pharmacists to map the order fulfilment journey, the YLabs team was able to better understand their needs and challenges and design a product they would actually use...KEY LESSONS: Engaging young people is crucial at all stages of the development and implementation process. This involvement should take place in a methodical way and go beyond tokenistic engagement. When involving young people, it is important to protect their safety and abide by ethical principles and legal regulations...Developers should demonstrate a clear understanding of the implementing environment. The implementing  environment includes the digital health interventions that already exist and the gaps that a new digital health  intervention might fill. Developers should ensure that a digital intervention is the best approach for the  implementing environment and for achieving overarching health goals | 1.FOR THE TEAM AND ESTABLISH GOALS: PRINCIPLE FOR DIGITAL DEVELOPMENT: Engage diverse experts across disciplines, countries and industries throughout the project life cycle. Create an engagement plan to apply this expertise at all phases and incorporate insights through feedback loops. Look for tools and approaches from other sectors and publish your findings so that they are available to other groups and countries... IDENTIFY HEALTH SYSTEM CHALLENGE AND NEEDS : PRINCIPLES FOR DIGITAL DEVELOPMENT: Engage with your target end-users and consult existing research to develop an understanding of the people, networks, cultures, politics, data needs, infrastructure and markets that make up your ecosystem before designing your initiative or tool...Coordinate with other implementing organizations, civil society and the government early on to learn from successful and unsuccessful initiatives in the ecosystem, to avoid duplicating efforts and to integrate with existing technical systems more easily...Incorporate multiple user types and stakeholders in each phase of the project life cycle to direct feature needs and revise the design. Here, users are people who will interact directly with the tool or system, and stakeholders are people who will be affected by or have an interest in the tool or system, such as people whose data are being collected, government officials or researchers who may study the data collected. Design tools that improve users’ current processes, saving time, using fewer resources and improving quality. Develop a context-appropriate digital implementation informed by end-users’ priorities and needs, considering the ecosystem and accepting that some digital approaches will not be appropriate. Develop the digital enterprise in an incremental and iterative manner, with clear objectives and purpose in mind. Ensure that the design is sensitive to and considers the needs of the historically underserved. Embrace an iterative process that allows for incorporating feedback and adapting your implementation after initial testing and launch. Be open about setting expectations and let people opt out of participating in the design process... 3.. DETERMINE APPROPRIATE DEIGITAL HEALTH INTERVENTION: PRINCIPLES OF DIGITAL DEVELOPMENT: Define and communicate what being open means for your initiative. Adopt and expand on existing open standards, such as Health Level 7 Fast Healthcare Interoperability Resource (HL7 FHIR): specifications developed by, agreed to, adopted by and maintained by a community that enable sharing of data across digital applications and the digital health platform. Share nonsensitive data after ensuring that data privacy needs are addressed; to  encourage open innovation by any group or sector, do not place restrictions on data use. Use existing open source and open standards–based software where appropriate to help automate data sharing, connect your tool or system with others and add flexibility to adapt to future needs. Develop any new software code to be open source, which anyone can view, copy, modify and share, and distribute the code in public repositories. Enable innovation by sharing freely without restrictions, collaborating widely and co-creating tools when it makes sense in your context...LEGISLATION, POLICY AND COMPLIANCE: Adopt national policies and legislation in priority areas; review sectoral policies for alignment and comprehensiveness; establish regular policy reviews. + Create a legal and enforcement environment to establish trust and protection for consumers and industry in eHealth practice and systems. |
| **Environmental** | no mention | THIS IS NOT mentioned ENVIRONEMNTAL PURPOSE BUT IT COULD HELP IN THAT :3 DETERMINE APPROPRIATE DEIGITAL HEALTH INTERVENTION: PRINCIPLES OF DIGITAL DEVELOPMENT: Identify the existing technology tools (local and global), data and frameworks being used by your target population, in your geography or in your sector. Evaluate how these could be reused, modified or extended for use in your program. + Develop modular, interoperable approaches instead of those that stand alone or are attempting to be all-encompassing in their features. Interoperability will ensure that you can adopt and build on components from others and that others can adopt and build on your tool in the future; and swap out systems when improved – standards based –solutions become available.+ Collaborate with other digital development practitioners through technical working groups, communities of practice and other knowledge-sharing events to become aware of existing tools and to build relationships that could lead to the future reuse and improvement of your tool... |
| **Economical** | ….Secure adequate resources for conducting a landscape analysis and a needs assessment; these are often underfunded activities….Do a thorough analysis of existing digital products locally and globally, to avoid duplication of what is already in use…Carefully cost and map out a timeline for the development of the digital intervention, building in time for rounds of user testing and refinement and exploring whether the team has the capacity, time and budget to build the intervention...The pace, timeline and scope of digital health interventions are often determined by available funding and the priorities of funding entities. This annex provides a funder’s perspective on important lessons related to supporting the development and implementation of youth-centred digital health interventions... | DETERMINE APPROPRIATE DEIGITAL HEALTH INTERVENTION:STRATEGY AND INVESTMENT: Align financing with priorities; donor, government and private-sector funding identified for medium term. WORKFORCE: Make eHealth knowledge and skills available through internal expertise, technical cooperation or the private sector. Build national, regional and specialized networks for eHealth implementation. Establish eHealth education and training programmes for health workforce capacity-building. STANDARDS AND INTEROPERABILITY: Introduce standards that enable consistent and accurate collection and exchange of health information across health systems and services. INFRASTRUCTURE: Form the foundations for electronic information exchange across geographical and health-sector boundaries. This includes the physical infrastructure (e.g. networks), core services and applications that underpin a national eHealth environment. SERVICES AND APPLICATIONS: Provide tangible means for enabling services and systems that deliver health content and; access to, and exchange and management of information and content. Users include the general public, patients, providers, insurance, and others. The means may be supplied by government or commercially. |
| **Measurements** | | |
| **Social** | general concept of the framework: equity : Equity means ensuring fair and impartial access to the digital health intervention. This has proven to be a challenge on both the local and global levels because access to mobile phones can vary between groups. For example, women in low- and middle-income countries are 10% less likely to own a mobile phone and 26% less likely to use mobile internet than men (27, 28). Rural populations in those countries are 40% less likely to use mobile internet than urban populations (17). The digital divide also mirrors prevailing economic gaps, amplifying the advantages of wealthier people and failing to deliver opportunities to the poorest and most disadvantaged (20). Nearly 90% of young people who are not using the internet live in Africa or in the Asia-Pacific region. In 2017, Africa had the highest proportion of nonusers aged 15 to 24...1 lists examples of questions that can be answered through a combination of literature review, community consultation and formative research. It is important to keep in mind when collecting and interpreting this information that young people are a diverse group of individuals with varying needs... QUESTIONS RECOMMENDED FOR LANDSCAPE ANALYSIS AND NEEDS ASSESSMENT : Individual level Who are the intended users of the intervention?...What are their literacy and digital literacy levels?...Where are they geographically located?...What are their education levels?...What are their health interests, concerns and priorities?...Individual level – digital channels...What devices are young people using?...What digital channels, apps and websites are most popular among them?...What are they using the technology and delivery channels for?...What are their digital habits, including when and where they use their devices and how much time they spend on them?...How affordable is internet/phone access for young people?...What does phone ownership look like among young people? Do they share phones?...In what ways do young people safeguard digital information in their day-to-day lives?...Are there privacy and confidentiality limitations in their current patterns of technology use (e.g., accessing the internet at internet cafes, sharing phones)?...Interpersonal level What are young people’s family environments like? ... What are the social influences on young people? ...How do they engage with each other as family members, friends or romantic partners? Community level What health interventions and programs, both digital and nondigital, already exist for young people in the community? ...What content already exists for young people on the intervention topic? ...What types of people in the community are involved with young people? ...Who are potential partners for developing, testing, delivering or promoting the intervention? ...What technology or delivery channels are perceived to have the greatest reach and influence in the community? ...What community-level challenges are associated with young people accessing relevant health information or services? ...What are the social expectations regarding young people’s roles, behaviours and positions in their families and communities? ... What types of information are young people seeking out or lacking that a digital health intervention could help address? ...Public health and public policy level: What current health statistics are available on young people? ...What regional or national health programs or campaigns are currently in progress? ...What current health policies focus on young people? ...What current national policies relate to digital programming? ... What is the state of the region or country’s digital and technology infrastructure and capabilities? ...What are the relevant local, regional and international laws/standards around consent and data protection? | 4. DETERMINE APPROPIATE DIGITAL HEALHT INTERVENTIONS: Successful deployment of digital health applications requires a thorough knowledge of the ecosystem where the interventions will be deployed and whether they can be supported in that environment. Understanding this context can inform the feasibility of implementing the digital health enterprise, as well as demonstrate where system integrations will be required. For example, in settings with limited infrastructure and governance structures, it may be prudent to opt for less complex digital health implementations until these building-block enabling factors evolve to a more mature state. Regardless, each subsequent investment in digital health should contribute cumulative value to the functioning of the digital health enterprise, addressing health needs within the health programme and across the health system. Investments in ball-of-mud health software characterized by an evolving agglomeration of functions, originating without a predetermined scope or design pattern, which are monolithic contribute to an accumulation of technical debt and are not advised (see Fig. 1.3.1)...The national digital health strategy, investment roadmap, country assessment of digital maturity, national inventory of digital assets and enterprise architecture documentation, if available, should serve as a starting point for understanding the priorities and state of the national digital health ecosystem, and hence the feasibility of selected digital health interventions and the context for integrating the prioritized interventions into the national system (see Fig. 1.1.4). The digital health strategy outlines a country’s vision as it relates to the enabling environment (see Fig. 4.2.1), such as legal, regulatory and policy frameworks and ICT workforce needs, as well as the ICT environment, including infrastructure and foundational architecture. ENABLING ENVIRONMENT: LEADERSHIP, GOVERNANCE AND MULTISECTOR ENGAGEMENT: Direct and coordinate eHealth at the national level; ensure alignment with health goals and political support; promote awareness and engage stakeholders.+ Use mechanisms, expertise, coordination and partnerships to develop or adopt eHealth components (e.g. standards). + Support and empower required change, implementation of recommendations and monitoring results for delivery of expected benefits...STRATEGY AND INVESTMENT: Ensure a responsive strategy and plan for the national eHealth environment. Lead planning, with involvement of major stakeholders and sectors...DETERMINE WHAT THE INTERVENITONS WILL NEED TO DO: The requirements should not be just digitizing the processes identified in the current-state workflows developed in Chapter 3; also think through ways that introducing efficiencies can optimize the performance of the health system and programme area. Answer these three questions to determine the requirements (see Fig. 4.3.1).1. What do members of the planning team (stakeholders) expect the digital health interventions to do for them? 2. What do end-users expect the digital health interventions to do for them? 3. What do beneficiaries expect the digital health interventions to do for them? ...IDENTIFY FUNCTIONAL REQUIREMENTS AND END USER STORIES: Functional requirements describe what the digital health application needs to do to address the health system challenges identified in Chapter 3. These requirements answer the question, “What does the intervention need to do to help overcome a health system challenge?” Software developers use the functional requirements as a reference to ensure that the intervention meets the needs of the targeted end-users. The functional requirements consist of simple statements that summarize what the end-user needs the digital health intervention to do (see Table 4.3.1.1for an example). Start with a brainstorming session to generate all possible scenarios that your team can imagine in a logical sequence along the process workflow developed in Chapter 3. Consider all end-users who will access the intervention directly or who will need to access information provided by the intervention to make decisions...UNDERSTAND AND MANAGE EXPECTATION FROM END-USERS AND STAKEHOLDERS.: It is also essential to understand how the digital health implementation will work for everyone involved and how to improve the usability and value of the applications for end-users. Some functions may not be part of a end-user’s workflow or direct experience but are relevant to the stakeholders. A senior policymaker may need to see aggregate performance data characterized by geographic region, for example, but a health worker working in close contact with communities may not have immediate use for this macrolevel information. Refer to the current-state workflow diagrams, which you created in Chapter 3, to determine who performs what tasks within a health system and their roles as beneficiaries, end-users or stakeholders. Consider three distinct groups when describing what the intervention needs to do (the functional requirements): » end-users, the people who will actually use the intervention » beneficiaries, individuals or groups whose health the intervention is intended to improve » other stakeholders, those with a keen interest in the success of the digital health implementation and the health programme, such as members of  the planning team. Understanding the perceptions, roles and responsibilities, as well as the motivations, of the people who will interact with or be affected by the digital health intervention ensures that the intervention responds to these human needs. Make the lists of these actors as broad or as close to the health programme as your team feels is necessary. Briefly describe each kind of stakeholder or end-user and assess their potential responses to identified digital health interventions (see Fig. 4.3.2.1 for an example). Once this is done, the  design team may choose to modify the requirements to mitigate potential risks and ensure that the digital health implementation meets broader needs... |
| **Environmental** | not mention |  |
| **Economical** | sustainability principle in this framework means financial models |  |
| **Outcomes (type of tool or purpose i.e., Adoption or health treatment’ s domains)** | main general concept: Having an iterative process means being open to change and factoring in time to modify and adapt the intervention. At the start of the process, it is easy to have a preconceived idea of what the intervention will look like. But current practices and field experiences indicate the need to include many points throughout the development and implementation process for collecting data and feedback, prototyping, testing, learning and updating the intervention to incrementally and progressively improve each “draft version.” This iterative process may ultimately lead to pivoting away from the original notions... Be realistic when identifying expected outcomes of the intervention. If outcomes are too dependent on broader factors outside the users’ control, it may be difficult to observe meaningful changes and attribute those changes to the intervention. A theory-driven approach can include indicators for changes directly influenced by the intervention as well as intermediate and longer-term outcomes that the intervention may influence to a lesser extent...Look to existing youth-related results frameworks and conceptual models as sources. A robust and diverse set of existing theories are available in the areas of behaviour change, communication and psychology, many of which have been tested in a variety of health contexts...Useful workshop strategies include using different colours of sticky notes on a large wall to depict the relationship between activities and desired outcomes (immediate, intermediate and long-term), individual and external factors and conditions needed to achieve the desired outcomes, activities needed to move from one outcome to the next, the rationale for each link in the causal pathway, and M&E indicators...Finding the underlying theory in two interventionsThe Unified Theory of Behaviour describes the factors that affect behavioural intention and how behavioural intention and external factors affect behaviour. (See the two figures below.) Planned Parenthood Federation of America (PPFA) has used the Unified Theory of Behaviour to inform the development of digital interventions, including an educational video series about consent and a birth control and period tracker application. In 2016, PPFA released the video series on YouTube to help young people understand consent and encourage them to ask for consent in their sexual relationships. The videos promote the behavioural belief that asking for consent will lead to better sex. They also normalize asking for consent by emphasizing that people who are like the viewer generally ask for consent. The cast of the series is diverse in terms of race, gender and sexual orientation, which allows viewers to relate the situations to their self-concept. The sexy and fun conversations push against negative emotions (such as fear or discomfort) that are often associated with asking for consent. Finally, by providing actual language to comfortably ask for consent, the series strengthens viewers’ sense of self-efficacy. In 2016, PPFA also launched Spot On Period Tracker, an app that allows users to track and manage their menstrual periods and birth control use. The app focuses primarily on external factors that can lead to behaviour change, since those who download the app probably want to track their birth control use and/or periods. For birth control, the app targets users’ knowledge and skills, uses a reminder system to create new habits and provides notifications to emphasize the salience of taking birth control regularly. It also asks users questions to account for environmental constraints, such as their ability to access their birth control earlier than usual if medical guidance recommends it. These constraints are built into algorithms that personalize next steps if the user has made a mistake with her birth control...Human-centred design The people designing digital health interventions for young people are usually adults (42). To better understand and work with young people, they can benefit from using an approach known as human-centred design (or user centred design), which focuses on users’ needs, wants and limitations (25, 43). Designing with the intended users in mind is crucial to developing successful interventions (25, 44). Key human-centred design approaches include: » Personas. Personas are generic descriptions of the types of people involved in or benefiting from the intervention (45). Personas help the core team and other participants view the objectives and challenges of the effort from the vantage point of the people who will receive the intervention. Personas also help align the team and other participants around shared definitions and perceptions. Finally, they provide a common point of reference for those who deliver the intervention, those who monitor or supervise it and, ultimately, those who receive it (45).» User journeys. User journeys are a way to visualize the user’s experience with the intervention from beginning to end, from the moment of awareness of the intervention to the decision to engage with it, the first interactions, subsequent engagement with it and being affected by it (46).» Co-design workshops. Co-design workshops convene the intended users, bring them into the design process (46) and build trust. The goal of such a workshop is to identify what health information the intended users want to know about and how they want the information delivered to them, including the look and feel of the digital solution. This is an opportunity to collaborate with young people and involve them in design decisions, not just to hear from them (46). The workshop should be a safe space where young people can discuss these health topics openly and share what they need most out of the intervention... | BG:... The process outlined in the Guide is also informed by the Principles for Digital Development, which help stakeholders effectively and appropriately apply digital technologies in their health programmes (digitalprinciples.org/principles). Core tenets of these nine principles will be referenced throughout this Guide.1.FOR THE TEAM AND ESTABLISH GOALS : Once you are ready to start using this Guide, the first step is to form the team and establish goals for your investments in the digital health enterprise. In this chapter, you will determine team roles and responsibilities, develop a common understanding of the health programme’s goals, and begin to understand how the health programme functions across all levels of the health system...National digital health/eHealth strategy (if one exists) + Documents including health programme objectives, progress and any evaluations + Organograms describing directorates/departments in the Ministry of Health (MOH) and relevant government bodies, such as Ministry of ICT, civil registrars and so on. PRINCIPLE FOR DIGITAL DEVELOPMETN: Understand how your work fits into the global development landscape. Identify others working on the same problem in other geographies and determine if there is a community of practice that relates to your work. Find the technical leaders through virtual networks or communities of practice, such as the Global Digital Health Network, the Asia eHealth Information Network (AeHIN), Global Digital Health Partnership, Digital Health and Interoperability Working Group, Health Data Collaborative, African Alliance for Digital Health and/or Implementing Best Practices Initiative, who can help you disseminate your work to other teams, regions and countries. Plan to collaborate from the beginning. Build collaborative activities into proposals, work plans, budgets and job descriptions. Identify indicators for measuring collaboration in your M&E plan... DEVELOP A COMMON UNDERSTANDING OF THE HEALTH PROGRAMMES NEEDS AND GOALS: Once you have formed the planning team, convene them and any necessary additional stakeholders to articulate a common understanding of the main goals of the health programme and how they align with your country’s national digital health strategy (if there is one). The team should also review core programme documents, data and assessment reports that describe the programme’s goals and objectives, including how it has performed to date...In this review of health programme documents, aim to clearly identify the following: 1. Short- and long-term goals and objectives of the health programme a. Assess how the program aligns with priorities under the national strategic health plan or other government strategies for investing in health. Ensure that all stakeholders have a shared understanding of the health programme’s goals. b. Assess how the health programme aligns with the national digital health strategy (if one exists). For example, the national digital health strategy might include overarching goals like “improved access to health services”. This may be followed by “digital health outcomes” (possibly stated as “eHealth outcomes” or “health ICT outcomes”, as in the example of Nigeria in Fig. 2.2.1), such as “effective use of telemedicine and ICT for health worker training and support” (48). c. Assess how well these goals align with the needs of the population that the health programme targets. Should the health programme focus on particular populations or groups to improve equity and coverage?...UNDERSTAND PROGRAMME OPERATIONS ACROSS LEVELS OF THE HEALTH SYSTEM: All stakeholders need to understand how the current health programme operates in practice, including the workflows and information flows across all levels of the health system (a focus of Chapter 3). You may find it helpful to document the structure of the health system and the types of workers and their roles at each level of the system. Resources like organizational diagrams (organograms) and health workforce operational guidance may exist that describe the health programme’s management, human-resource structure and expected roles and responsibilities. During this step, try to describe the following:» the different tiers of the health system, including the community, district and provincial levels» the types of health provider and management workforce cadres associated with the programme area and their relationships with the levels of the health system» linkages across different health programme areas, such as immunizations and antenatal care» the names of the health facility types and the health workforce cadres associated with providing care within the health programme area. You may find it helpful to create a diagram using lines and arrows to illustrate the levels of the health system, facility types and cadres, their relationships to one another and their collective responsibilities (see Fig. 2.3.1)...IDENTIFY HEALTH SYSTEM CHALLENGE AND NEEDS: Now that you have identified and described important processes in the health programme, you can further examine the user journey to understand where to make improvements. Describing these processes as they occur, with as much input from programme implementers with on-the-ground experience as possible, is vital to capture events as they typically happen, rather than how they are officially supposed to happen or imagined happening in theory. Detailing tasks, or the specific activities within a health programme process, will uncover opportunities to improve the overall process. Workflows, or task flow diagrams, are one way of illustrating the user journey. Workflow diagrams are visual representations of the progression of activities (tasks, events and interactions) performed within a health programme process. These diagrams help visualize specific activities within the process and illustrate the interactions between the personas who perform those activities (see Fig. 3.1.2.1). The result of one task generally triggers another task, until the final process objective is reached. All tasks associated with the process being mapped should appear at least once on the workflow diagram. These diagrams also map how information moves through the system and can be used to identify and illustrate where bottlenecks occur. (See Box 3.1.2.2 for a description of symbols generally used in workflow diagrams.) Develop workflows for the different processes through discussions with the people who provide services within the health programme. Try to get multiple perspectives of how work is actually performed, rather than how health system managers may think (or hope) the work is done. This should also be complemented by mapping the range of processes and interventions delivered during a given interaction. By doing so, you can ensure that you avoid designing around a single health need but missing other interactions that the health provider may have at the same time with the same patient. Ultimately, any separate workflows that are part of a process should tie together when the analysis is complete. As stakeholders review activities within the different processes, they can reflect on the challenges that prevent achieving the outlined activities. When designing an intervention, you could use the workflow diagrams to explain how the health programme works, the interrelations between people and places and the issues to address in order to improve performance. You may also want to review common workflow diagrams documented in WHO digital accelerator kits (22) relevant to the programme area(s), which can offer a starting point for discussion and comparison with your own workflow systems...**QI** As you create the workflows, challenges – or bottlenecks – should emerge. These are areas where failures in service delivery occur, where health workers experience frustrations or even where patients may be lost to follow-up. Bottlenecks are the specific gaps that prevent personas from reaching their goals of success and achieving positive health outcomes. Bottlenecks contribute to the suboptimal implementation of health programmes and are often causes of the failure to meet the programme’s goals. For example, in the workflow shown in Fig. 3.1.2.1, you may find that clients routinely do not show up for their expected first encounters (Task 1). Further discussion may reveal that clients experience difficulties (such as when articulating their needs) that prevent them from benefiting from a health worker’s consultation and diagnosis (Task 2). Issues like inaccurate diagnoses or adherence to clinical protocols during consultations may emerge as additional bottlenecks associated with the health worker, also occurring at Task 2. You could validate that the workflow diagrams are accurate through observations and interviews with stakeholders who are involved in performing the work, as well as with clients attempting to access the health system. This validation should take place with the health workers and clients who know what happens on a daily basis and who can share rich insights into how these activities work in practice, rather than with directors and supervisors. You could organize discussions with those at the frontlines who can additionally articulate their challenges in delivering health services, highlighting the bottlenecks. Reviewing the workflow diagram with them and explaining your goals and what you will do with the information will improve accuracy in representing the workflows. This process of engaging with personas will help document gaps between the current state and the desired future state of the health programme by identifying the following: inefficiencies or gaps, efficiencies that can be gained with repeatable ,tasks,, redundancy in tasks, such as information collected more than once , blocks to the optimal flow from one task to the next...DETERMINE APPROPIATE DIGITAL HEALHT INTERVENTION : DETERMINE AND SELECT DIGITAL HEALHT INTERVENTIONS FOR THE PRIORITIZED HEALTH SYSTEM CHALLENGES: Over the past two decades, a variety of digital health approaches have been tested as ways of alleviating health system challenges that have not otherwise been adequately addressed. From health-promotion messages sent to clients to applications that track stock levels, digital health interventions have been used individually (siloed) or combined with shared services using data exchange standards to form robust and extensible digital health enterprises. You could reflect on the root causes of each health system challenge to understand how a particular intervention may overcome or mitigate it (see Chapter3). Involve potential end-users (clients, health workers, supervisors and so on) at this stage as they may be instrumental in understanding whether and how digital health interventions can help address the identified issues. You may also find it appropriate to address a health system challenge by combining digital and nondigital approaches. To begin selecting digital health interventions, first review the WHO Classification of digital health interventions shown in Fig. 4.1.1 (4). This classification system presents the diverse ways that technology has been documented to support health system needs and address challenges (see Fig. 4.1.2). Each digital health intervention included in the Classification represents a discrete unit of technology functionality to address a health programme need or overcome a health system challenge. Furthermore, the Classification provides a standardized vocabulary that public health practitioners and software vendors can understand when expressing how the digital health intervention should function. Your review of this document should facilitate your understanding of the opportunities that may exist whenbuilding a digital health enterprise and how the digital health interventions will address identified health system challenges... |
| **SUSQI FRAMEWORK (this is the principles of the Centre of sustainable health care)** |  |  |
| **Prevention (SUSQI)Disease prevention and health promotion. All clinicians should be involved in prevention. Through broader advocacy and in individual patient care, specialties should aim to tackle underlying causes of disease – the social, economic and environmental determinants of health. Where possible, interventions should capture environmental co-benefits of healthy lifestyles, such as the improvements in air quality and carbon emissions from a shift to active travel.** | yes | NO |
| **patient empowerment and self-care (SUSQI)Patient education and empowerment. To reduce disease progression and pre-empt complications, many patients could be empowered to take on a greater role in the management of their own health and healthcare. Informed patients are also well placed to improve the coordination between clinical teams and reduce misunderstandings or duplication.** | yes | YES |
| **lean clinical pathways (SUSQI) Lean service delivery. Improving clinical decision-making in the selection and targeting of interventions will reduce lower value activities and their associated environmental impacts. Specialties can support this by describing the relevant patient pathways and providing clear, evidence-based guidance. Even where clinical input is of high value, a greater use of online records, email and telephone can reduce travel emissions by moving information in place of patients, staff and laboratory samples. Further efficiencies can follow from better integration of specialist services, such as diabetes, cardiovascular and renal care, which have a common patient base.** | yes | YES |
| **low carbon alternatives (SUSQI) Preferential use of treatment options and medical technologies with lower environmental impact. Inclusion of sustainability measures in the evaluation of medical technologies will allow service planners, clinicians and patients to choose clinically effective treatments with the best environmental profile and will encourage their further development.** | no | NO |
| **Efficient resource use-improving use of energy, transport, water, waste and equipment.** |  |  |
| **Strengths** |  |  |
| **Weakness** |  |  |
| **Gaps in research** | definition of sustainability: Sustainability can mean different things at each stage, but in general it calls for thinking through how the intervention will be able to stand on its own, beyond the initial development and implementation and beyond initial funding. Sustainability may also involve operating at a larger scale (with more users, more functionality and/or more features) in the long term. Building a sustainable intervention involves “beginning with the end in mind” and paying attention at the earliest stages to developing a financial model and enlisting supportive partners |  |
| **thoughts on this framework** | The guidance presented in this document is intended for digital health intervention designers, developers, implementers, researchers and funders. Newcomers to digital health can use it as a start-to-finish primer on how to collaboratively and responsibly develop youth-centred digital health interventions. |  |
