# Supplementary material for: Sustainable assessment in digital health interventions for primary care: A scoping review
Source: J Public Health Res. 2026 Jan 23;15(1):22799036251407196. doi: 10.1177/22799036251407196 (PMC12833121; doi:10.1177/22799036251407196)
Supplement: sj-pdf-4-phj-10.1177_22799036251407196 – Supplemental material for Sustainable assessment in digital health interventions for primary care: A scoping review [file sj-pdf-4-phj-10.1177_22799036251407196.pdf]

Appendix V-Inclusion check list

Inclusion checklist	Access	Wellbeing	Cibersecurity (trust)	Costeffectiveness	Financial Impact	Procurement analisys	Ecological impact	GHG emissions	Life Cycle	Digital Health Intervention-General	Artificial intelligence	Wearables-IoT	Mobile applications	Webpage	Patient portals	Community pharmacy	Dental	Optometry	Community health	Population health	Primary care - General	Developed of evaluation	OR Integration	OR Adoption	(DevE,I,A) Guidelines	(DevE,I,A) Models	(DevE,I,A)Standards	(DevE,I,A) Framework	Status - fulltext stage	Reason
Fahad Alanezi ; Turki Alanzi	no	no	no	yes			no	no	no				yes								yes	no	no	no	no	no	no	no	EXCLUDED	Integration
Rosalind et al. 2023																													NO access	
Ofir Ben-Assuli (2022)	no	no	no	yes			no	no	no	no	no	no	no	no	no						yes	no	no	no	no	no	no	no	EXCLUDED	Develop DHI
Sean C. Broomhead, Maurice Mars and Richard E. Scott (2023)	no	no	no			yes	no	no	no			yes	yes	yes							yes		yes					yes	EXCLUDED	only economic
Sean C. Broomhead, Maurice Mars, Richard E. Scott & Tom Jones (2020)	yes			yes			no	no	no			yes	yes	yes							yes					yes			INCLUDED	
Sean C. Broomhead, Maurice Mars, and Tom Jones (2021)	no	no	no	yes			no	no	no			yes	yes	yes							yes	no	no	no	no	no	no	no	EXCLUDED	Develop DHI
Lior Carmi, Mishael Zohar, and Gianluigi M. Riva (2022)			yes				no	no	no			yes	yes	yes							yes	no	no	no	no	no	no	no	EXCLUDED	Develop DHI
John Chuo,Michelle L. Macy, and Scott A. Lorch, 2020	yes					yes	no	no	no	yes										yes								yes	INCLUDED	
Alison Curfman, David McSwain, and Kit Simpson (2021)	yes			yes			no	no	no	yes						no	no	no	no	no	no	no	no	no	no	no	no	no	EXCLUDED	NO PRIMARY CARE NO EVALUATION DHI IS COMPARISIO N
Keshia R De Guzman et.al. (2021)	no	no	no	yes			no	no	no	yes											yes	no	no	no	no	no	no	no	EXCLUDED	Develop DHI
M. Fragao-Marques and T. Ozben	no	no	no	no	no	no	yes			no	no	no	no	no	no						yes	no	no	no	no	no	no	no	EXCLUDED	Develop DHI
Gentili, Andrea ;Failla, Giovanna ;Melnyk, Andriy ;Puleo, Valeria ;Tanna, Gian Luca Di ;Ricciardi, Walter ;Cascini, Fidelia	no	no	no	yes			no	no	no	yes											yes	no	no	no	no	no	no	no	EXCLUDED	Develop DHI
Ghani, Zartashia ;Jarl, Johan ;Sanmartin Berglund, Johan ;Andersson, Martin ;Anderberg, Peter	no	no	no	yes			no	no	no	yes											yes	no	no	no	no	no	no	no	EXCLUDED	Develop DHI
Grigorovich, Alisa ;Xi, Min ;Lam, Natascha ;Pakosh, Maureen ;Chan, Brian C. F.	no	no	no	yes			no	no	no	yes						no	no	no	no	no	no	no	no	no	no	no	no	no	EXCLUDED	Develop DHI
Gu, Wenjun ;Shu, Luchengchen ;Chen, Wanning ;Wang, Jinhua ;Wu, Dingfeng ;Ai, Zisheng ;Li, Jiyu	no	no	no	no	no	no	no	no	no	yes						no	no	no	no	no	no	yes							EXCLUDED	Integration
Haig, Madeleine ;Main, Caitlin ;Chavez, Danitza ;Kanavos, Panos	yes	yes	yes	yes			no	no	no	yes											yes	yes							INCLUDED	

Inclusion checklist	Access	Wellbeing	Cibersecurity (trust)	Costeffectiveness	Financial Impact	Procurement analisys	Ecological impact	GHG emissions	Life Cycle	Digital Health Intervention-General	Artificial intelligence	Wearebles-IoT	Mobile applications	Webpage	Patient portals	Community pharmacy	Dental	Optometry	Community health	Population health	Primary care - General	Developed of evaluation	OR Integration	OR Adoption	(DevE,I,A) Guidelines	(DevE,I,A) Models	(DevE,I,A)Standards	(DevE,I,A) Framework	Status - fulltext stage	Reason
Hilty, Donald M.<br>Serhal, Eva<br>Crawford, Allison																													NO access	
Jo, Youngji<br>LeFevre, Amnesty Elizabeth<br>Ali, Hasmot<br>Mehra, Sucheta<br>Alland, Kelsey<br>Shaikh, Saijuddin<br>Haque, Rezwanul<br>Pak, Esther Semee<br>Chowdhury, Mridul<br>Labrique, Alain B.	no	no	no	yes			no	no	no	yes						no	no	no	no	no	no	no	no	no	no	no	no	no	EXCLUDED	Develop Evaluation
Kiberu, Vincent M.<br>Mars, Maurice<br>Scott, Richard E.	no	no	no	no	no	no	no	no	no	no	no	no	no	no	no						yes	yes							EXCLUDED	
K. Kidholm, L. K. Jensen, T. Kjolhede, E. Nielsen and M. B. Hor	yes	yes		yes			no	no	no	yes											yes					yes			EXCLUDED	YEAR 2016
T. Krishnamurti, M. Birru Talabi, L. S. Callegari, T. M. Kazmerski and S. Borrero		yes		no	no	no	no	no	no	yes									yes			no	no	no	no	no	no	no	EXCLUDED	Develop evaluation
Le Douarin, Yann<br>Traversino, Yannick<br>Graciet, Armelle<br>Josseran, Anne	no	no	no	yes			no	no	no	yes											yes	yes							EXCLUDED	language
Liddy, Clare<br>Moroz, Isabella<br>Afkham, Amir<br>Keely, Erin		yes		yes			no	no	no	yes											yes	no	no	no	no	no	no	no	EXCLUDED	Develop evaluation
Lin, Tzu-Wei			yes	no	no	no	no	no	no	yes											yes	no	no	no	no	no	no	no	EXCLUDED	Develop evaluation
Mastoi, Qurat-Ul-Ain<br>Ying Wah, Teh<br>Gopal Raj, Ram<br>Lakhan, Abdullah	no	no	no	yes			no	no	no			yes									yes	no	no	no	no	no	no	no	EXCLUDED	Develop evaluation
Nadhamuni, Sunita<br>John, Oommen<br>Kulkarni, Mallari<br>Nanda, Eshan<br>Venkatraman, Sethuraman<br>Varma, Devesh<br>Balsari, Satchit<br>Gudi, Nachiket<br>Samantaray, Shantidev<br>Reddy, Haritha<br>Sheel, Vikas			yes	no	no	no	no	no	no	yes											yes				yes				EXCLUDED	Develop evaluation

Inclusion checklist	Access	Wellbeing	Cibersecurity (trust)	Costeffectiveness	Financial Impact	Procurement analisys	Ecological impact	GHG emissions	Life Cycle	Digital Health Intervention-General	Artificial intelligence	Wearebles-IoT	Mobile applications	Webpage	Patient portals	Community pharmacy	Dental	Optometry	Community health	Population health	Primary care - General	Developed of evaluation	OR Integration	OR Adoption	(DevE,I,A) Guidelines	(DevE,I,A) Models	(DevE,I,A)Standards	(DevE,I,A) Framework	Status - fulltext stage	Reason
Poleto, Thiago<br>Silva, Maisa Mendonca<br>Clemente, Tharcylla<br>Rebecca Negreiros<br>de Gusmao, Ana Paula<br>Henriques<br>Araujo, Ana Paula de Barros<br>Costa, Ana Paula Cabral<br>Seixas			yes	no	no	no	no	no	no	yes											yes							yes	EXCLUDED	Technical framework
Powers, Brenton<br>Bucher, Amy	no	no	no		yes		no	no	no	yes										yes		no	no	no	no	no	no	no	EXCLUDED	Develop evaluation
Ramanadhan, Shoba<br>Ganapathy, Krishnan<br>Nukala, Lovakanth<br>Rajagopalan, Subramaniya<br>Camillus, John C.	yes	yes		yes			no	no	no	no	no	no	no	no	no				yes									yes	EXCLUDED	Develop DHI
Velayati, Farnia<br>Ayatollahi, Haleh<br>Hemmat, Morteza<br>Dehghan, Reza	yes	yes				yes	no	no	no	yes										yes		yes							EXCLUDED	Develop evaluation framework for integration not for bussiness
C. J. Wang, T. T. Liu, J. Car and B. Zuckerman		yes		yes			no	no	no	yes										yes		no	no	no	no	no	no	no	EXCLUDED	Develop evaluation
A. A. Abdellatif, M. S. Allahham, A. Mohamed, A. Erbad and M. Guizani				yes			no	no	no	yes										yes		no	no	no	no	no	no	no	EXCLUDED	Develop evaluation
S. M. S. Al-Gayar, I. Marin, M. Almalchy, N. Goga, N. Al-Habeeb and C. Taslitschi	yes		yes				no	no	no	yes										yes		no	no	no	no	no	no	no	EXCLUDED	Develop evaluation
B. Ali, M. A. Gregory and S. Li			yes				no	no	no	yes										yes		no	no	no	no	no	no	no	EXCLUDED	Develop evaluation
K. Babar and M. A. Shah			yes				no	no	no	yes										yes		no	no	no	no	no	no	no	EXCLUDED	Develop evaluation
M. Ebrahimabadi, M. Younis, W. Lalouani, A. Alshaeri and N. Karimi			yes				no	no	no	yes										yes		no	no	no	no	no	no	no	EXCLUDED	Develop evaluation
M. Prabhu<br>A. Hanumanthaiah	no	no	no	no	no	no	no	no	no	yes										yes		no	no	no	no	no	no	no	EXCLUDED	Develop evaluation
G. Hatzivasilis, O. Soultatos, S. Ioannidis, C. Verikoukis, G. Demetriou and C. Tsatsoulis			yes	yes			no	no	no			yes								yes					yes				EXCLUDED	technical framework
H. Kordestani, K. Barkaoui and W. Zahran			yes	no	no	no	no	no	no	yes										yes		no	no	no	no	no	no	no	EXCLUDED	Develop evaluation
J. Nie, Y. Hu, Y. Wang, S. Xia and X. Jiang	no	no	no	yes			no	no	no			yes								yes		no	no	no	no	no	no	no	EXCLUDED	Develop evaluation

Inclusion checklist	Access	Wellbeing	Cibersecurity (trust)	Costeffectivene ss	Financial Impact	Procurement analisys	Ecological impact	GHG emissions	Life Cycle	Digital Health Intervention-General	Artificial intelligence	Wearebles-IoT	Mobile applications	Webpage	Patient portals	Community pharmacy	Dental	Optometry	Community health	Population health	Primary care - General	Developed of evaluation	OR Integration	OR Adoption	(DevE,I,A) Guidelines	(DevE,I,A) Models	(DevE,I,A)Stand ards	(DevE,I,A) Framework	Status - fulltext stage	Reason
J. Nurmi, Y. Xu, J. Boutellier and B. Tan			yes	no	no	no	no	no	no	yes										yes		no	no	no	no	no	no	no	EXCLUDED	not evaluation by human is a machine laerning that give reslut on the ciber security of tech
P. Nuzzo, N. Bajaj, M. Masin, D. Kirov, R. Passerone and A. L. Sangiovanni-Vincentelli			yes	yes			no	no	no	yes										yes		no	no	no	no	no	no	no	EXCLUDED	Develop evaluation
M. A. R. Rahil, M. Waleed, S. Almajid, N. Bucheeri and Z. Bahri	no	no	no	yes			no	no	no	yes										yes		no	no	no	no	no	no	no	EXCLUDED	Develop evaluation
J. Randolph, M. J. H. Faruk, B. Saha, H. Shahriar, M. Valero, L. Zhao and N. Sakib			yes	no	no	no	no	no	no			yes				no	no	no	no	no	no	no	no	no	no	no	no	no	EXCLUDED	Develop evaluation
P. Rastogi, D. Singh and S. S. Bedi			yes	no	no	no	no	no	no			yes								yes		no	no	no	no	no	no	no	EXCLUDED	Develop evaluation
S. Sanyal, D. Wu and B. Nour			yes	no	no	no	no	no	no	yes										yes		no	no	no	no	no	no	no	EXCLUDED	it is a technical evaluation not a integration evaluation
P. Sundaravadivel, C. Tumwesigye, S. P. Mohanty and E. Kougianos	yes		yes	no	no	no	no	no	no			yes									yes	no	no	no	no	no	no	no	EXCLUDED	it is a technical framework
C. B. Wilfred, A. Beno, A. D. N, E. Thenmozhi, S. Bagavathy and S. S. Rani			yes	no	no	no	no	no	no			yes								yes		no	no	no	no	no	no	no	EXCLUDED	it is a technical framework
M. Zawish, N. Ashraf, R. I. Ansari and S. Davy	no	no	no	no	no	no	yes			yes										yes		no	no	no	no	no	no	no	EXCLUDED	it is a technical framework
X. Jia<br>M. Luo<br>H. Wang<br>J. Shen<br>D. He			yes	no	no	no	no	no	no	yes										yes		no	no	no	no	no	no	no	EXCLUDED	Develop evaluation
U. Tripathi<br>S. Sangani<br>L. L. Y. Liu<br>A. Lamontagne	no	no	no	no	no	no	no	no	no	yes										yes		no	no	no	no	no	no	no	EXCLUDED	Develop evaluation
S. Yu<br>K. Park			yes	yes								yes								yes		no	no	no	no	no	no	no	EXCLUDED	Develop evaluation
**SOCIAL**																														
M. G. Antonio and O. Petrovskaya	yes			no	no	no	no	no	no	yes										yes		yes						yes	INCLUDED	
M. Brown, E. O. Ofili, D. Okirie, P. Pemu, C. Franklin, Y. Suk, et al.	yes			yes						no	no	no	no	no	no					yes		no	no	no	no	no	no	no	EXCLUDED	Develop DHI
A. d'Elia, M. Gabbay, S. Rodgers, C. Kierans, E. Jones, I. Durrani, et al.	yes	yes	yes								yes									yes					yes				INCLUDED	

Inclusion checklist	Access	Wellbeing	Cibersecurity (trust)	Costeffectiveness	Financial Impact	Procurement analisys	Ecological impact	GHG emissions	Life Cycle	Digital Health Intervention-General	Artificial intelligence	Wearables-IoT	Mobile applications	Webpage	Patient portals	Community pharmacy	Dental	Optometry	Community health	Population health	Primary care - General	Developed of evaluation	OR Integration	OR Adoption	(DevE,I,A) Guidelines	(DevE,I,A) Models	(DevE,I,A)Standards	(DevE,I,A) Framework	Status - fulltext stage	Reason
C. Dulude, S. Sutherland, S. Vanderhout, W. J. King, C. Zuijdwijk, N. Major, et al.	yes	yes	yes	no	no	no	no	no	no	yes						no	no	no	no	no	no	yes							ask	it doesn't have other sustainable values and it is pediatric
Y. Q. Huang, L. Liu, Z. Goodarzi and J. A. Watt	no	no	no	no	no	no	no	no	no	yes										yes		no	no	no	no	no	no	no	EXCLUDED	Develop evaluation integration or adoption
Israni, Sonoo Thadaney<br>Matheny, Michael E.<br>Matlow, Ryan<br>Whicher, Danielle		yes		yes			no	no	no		yes									yes		no	no	no	no	no	no	no	EXCLUDED	not sustainable value only reflection n ethical and human aspects of IA integration
Kayser, Lars<br>Rossen, Sine<br>Karnoe, Astrid<br>Elsworth, Gerald<br>Vibe-Petersen, Jette<br>Christensen, Jesper Frank<br>Ried-Larsen, Mathias<br>Osborne, Richard H.	yes		yes	no	no	no	no	no	no	yes										yes		yes							ask	it doesn't have other sustainable values and it is pediatric
Kobeissi, Mahrokh M.<br>Hickey, Joanne V.	yes			no	no	no	no	no	no		yes									yes		yes							EXCLUDED	1 value
Lyles, Courtney R.<br>Nguyen, Oanh Kieu<br>Khoong, Elaine C.<br>Aguilera, Adrian<br>Sarkar, Urmimala	yes			no	no	no	no	no	no	yes										yes					yes				EXCLUDED	1 value
Miller, Sarah J.<br>Sly, Jamilia R.<br>Alcaraz, Kassandra I.<br>Ashing, Kimlin<br>Christy, Shannon M.<br>Gonzalez, Brian<br>Lu, Qian<br>Newton, Robert L.<br>Redmond, Michelle<br>Shen, Megan<br>Thomas-Purcell, Kamilah<br>Yi, Jean<br>Veinot, Tiffany<br>Meade, Cathy D.	yes			no	no	no	no	no	no	yes										yes								yes	EXCLUDED	1 value
Samuels-Kalow, Margaret<br>Jaffe, Todd<br>Zachrison, Kori	yes			no	no	no	no	no	no	yes										yes								yes	EXCLUDED	it doesn look to evaluate financial value but it mention it to into the access but only mention to improve grants

Inclusion checklist	Access	Wellbeing	Cibersecurity (trust)	Costeffectivess	Financial Impact	Procurement analisys	Ecological impact	GHG emissions	Life Cycle	Digital Health Intervention-General	Artificial intelligence	Wearebles-IoT	Mobile applications	Webpage	Patient portals	Community pharmacy	Dental	Optometry	Community health	Population health	Primary care - General	Developed of evaluation	OR Integration	OR Adoption	(DevE,I,A) Guidelines	(DevE,I,A) Models	(DevE,I,A)Standards	(DevE,I,A) Framework	Status - fulltext stage	Reason
Scheerens, Charlotte<br>Gilissen, Joni<br>Volow, Aiesha M.<br>Powell, Jana L.<br>Ferguson, Clarissa M.<br>Farrell, David<br>Li, Brookelle<br>Berry, Corinne<br>Sudore, Rebecca L.	no	no	no	no	no	no	no	no	no	yes										yes								yes	EXCLUDED	no sustainable value
Were, Martin C.<br>Sinha, Chaitali<br>Catalani, Caricia	yes			no	no	no	no	no	no	no	no	no	no	no	no					yes		no	no	no	no	no	no	no	EXCLUDED	DHI evaluation
Williams, Carmel	yes	yes	yes	yes	yes						yes									yes								yes	INCLUDED	
M. Wu<br>Q. Huang<br>S. Gao	yes																			yes								yes	EXCLUDED	hybrid no focus on ly in DHI
F. Ivars-Silva<br>R. J. F. Rossetti	yes	yes	yes	yes	yes	yes	yes	yes	yes	no	no	no	no	no	no	no	no	no	no	no	no	no	no	no	no	no	no	no	EXCLUDED	DHI evaluation
H. D. Mohammadian<br>V. Wittberg<br>M. Castro<br>G. Bolandian	yes	yes	yes	yes	yes	yes	yes	yes	yes	no	no	no	no	no	no	no	no	no	no	no	no							yes	EXCLUDED	no healthcare settings
H. Regenbrecht<br>S. Zwanenburg<br>T. Langlotz	yes	yes	yes	no	no	no	no	no	no	no	no	no	no	no	no	no	no	no	no	no	no							yes	EXCLUDED	no healthcare settings
M. Luna-delRisco<br>M. G. Palacio<br>C. A. A. Orozco<br>S. V. Moncada<br>L. G. Palacio<br>J. J. Q. Montealegre<br>I. Diaz-Forero	yes	yes		yes	yes					yes										yes		no	no	no	no	no	no	no	EXCLUDED	no evaluation framework
Socially determined organisation	yes			yes						yes										yes								yes	INCLUDED	???
Antonio, Marcy G.<br>Petrovskaya, Olga	yes			no	no	no	no	no	no	yes										yes								yes	EXCLUDED	focus on ly on social sustainability
Benda, Natalie C.<br>Veinot, Tiffany C.<br>Sieck, Cynthia J.<br>Ancker, Jessica S.	yes			no	no	no	no	no	no	yes										yes		no	no	no	no	no	no	no	EXCLUDED	1 sustianable value
Richardson, Safiya<br>Lawrence, Katharine<br>Schoenthaler, Antoinette M.<br>Mann, Devin	yes	yes		no	no	no	no	no	no	yes										yes		no	no	no	no	no	no	no	EXCLUDED	1 value
Demilade Adedinsewo; Lauren Eberly; Olayemi Sokumbi; Jorge Alberto Rodriguez; Christi A. Patten ;LaPrincess C. Brewer	yes			no	no	no	no	no	no	yes										yes								yes	EXCLUDED	1 value

Inclusion checklist	Access	Wellbeing	Cibersecurity (trust)	Costeffectiveness	Financial Impact	Procurement analisys	Ecological impact	GHG emissions	Life Cycle	Digital Health Intervention-General	Artificial intelligence	Wearebles-IoT	Mobile applications	Webpage	Patient portals	Community pharmacy	Dental	Optometry	Community health	Population health	Primary care - General	Developed of evaluation	OR Integration	OR Adoption	(DevE,I,A) Guidelines	(DevE,I,A) Models	(DevE,I,A)Standards	(DevE,I,A) Framework	Status - fulltext stage	Reason
Rima Gibbings a, Nilmini Wickramasinghe	yes			no	no	no	no	no	no	no	no	no	no	no	no					yes		no	no	no	no	no	no	no	EXCLUDED	noe evaluation of DHI
Ilona Kickbusch; Dario Piselli ; Anurag Agrawal; Ran Balicer ;Olivia Banner; Michael Adelhardt ; Emanuele Capobianco ; Christopher Fabian ; Amandeep Singh Gill; Deborah Lupton; Rohinton P Medhora; Njide Ndili ; Andrzej Ryś ; Nanjira Sambuli; Dykki Settle; Soumya Swaminathan ; Jeanette Vega Morales ; Miranda Wolpert ; Andrew W Wyckoff ; Lan Xue ; Brian Li Han Wong	yes			yes						yes										yes		no	no	no	no	no	no	no	EXCLUDED	develop evaluation
**ENVIRONMENTAL**																														
Lokmic-Tomkins, Zerina<br>Davies, Shauna<br>Block, Lorraine J.<br>Cochrane, Lindy<br>Dorin, Alan<br>von Gerich, Hanna<br>Lozada-Perezmitre, Erika<br>Reid, Lisa<br>Peltonen, Laura-Maria	no	no	no				yes	yes	yes	yes										yes		no	no	no	no	no	no	no	EXCLUDED	not an evaluation framwork
Pickard Strange, Martha<br>Booth, Amy<br>Akiki, Melissa<br>Wieringa, Sietse<br>Shaw, Sara E.	yes			yes	yes	yes	yes	yes	yes	yes										yes		no	no	no	no	no	no	no	EXCLUDED	No an evaluation framework
Savoldelli, Anna<br>Landi, Daniele<br>Rizzi, Caterina							yes	yes	yes	no	no	no	no	no	no					yes		no	no	no	no	no	no	no	EXCLUDED	No digital healht interventions
Cristina Richie	no	no	no	no	no	no	yes			yes										yes		no	no	no	no	no	no	no	EXCLUDED	no an evaluation framework
Saisó, Sebastián García<br>Marti, Myrna C.<br>Medina, Felipe Mejia<br>Pascha, Victoria Malek<br>Nelson, Jennifer<br>Tejerina, Luis<br>Bagolle, Alexander<br>D'Agostino, Marcelo	yes	yes	yes	yes						yes										yes					yes				INCLUDED	
Hayes, Angela							yes			no	no	no	no	no	no					yes		no	no	no	no	no	no	no	EXCLUDED	evaluation development nr digital healt tools focus
NICE Framework																													INCLUDED	
WHO guidelines																													INCLUDED	

Inclusion checklist	Access	Wellbeing	Cibersecurity (trust)	Costeffectiveness	Financial Impact	Procurement analisys	Ecological impact	GHG emissions	Life Cycle	Digital Health Intervention-General	Artificial intelligence	Wearebles-IoT	Mobile applications	Webpage	Patient portals	Community pharmacy	Dental	Optometry	Community health	Population health	Primary care - General	Developed of evaluation	OR Integration	OR Adoption	(DevE,I,A) Guidelines	(DevE,I,A) Models	(DevE,I,A)Standards	(DevE,I,A) Framework	Status - fulltext stage	Reason
WHO young people DHI guidance																													INCLUDED	
Governing Health Futures 2030 Commission	yes			yes			yes			yes										yes								yes	INCLUDED	
