# Supplementary material for: Sustainable assessment in digital health interventions for primary care: A scoping review
Source: J Public Health Res. 2026 Jan 23;15(1):22799036251407196. doi: 10.1177/22799036251407196 (PMC12833121; doi:10.1177/22799036251407196)
Supplement: sj-pdf-5-phj-10.1177_22799036251407196 – Supplemental material for Sustainable assessment in digital health interventions for primary care: A scoping review [file sj-pdf-5-phj-10.1177_22799036251407196.pdf]

Appendix VIII-data charting

scopus (18/01/2023)						
		"sustainable value" 1245			Most articles journal in sustianble value	
Sustainable DevelopmentShow preview for(321)	CommerceShow preview for(21)	Waste ManagementShow preview for(16)	Case StudyShow preview for(11)	Controlled StudyShow preview for(9)	Sustainability Switzerland	96
SustainabilityShow preview for(301)	Environmental ManagementShow preview for(21)	Conceptual FrameworkShow preview for(15)	Environmental PerformanceShow preview for(11)	Digital TechnologiesShow preview for(9)	Journal Of Cleaner Production	55
Sustainable ValuesShow preview for(222)	Corporate SustainabilityShow preview for(20)	Industrial ResearchShow preview for(15)	EthicsShow preview for(11)	EcosystemsShow preview for(9)	Procedia CIRP	19
Sustainable ValueShow preview for(106)	Information SystemsShow preview for(20)	Risk AssessmentShow preview for(15)	Information ManagementShow preview for(11)	Lean ManufacturingShow preview for(9)	Csr Sustainability Ethics And Governance	18
Value CreationShow preview for(67)	ProfitabilityShow preview for(20)	AgricultureShow preview for(14)	Intellectual CapitalShow preview for(11)	Life Cycle AnalysisShow preview for(9)	Business Strategy And The Environment	16
InnovationShow preview for(60)	Sustainability AssessmentShow preview for(20)	BusinessShow preview for(14)	ItalyShow preview for(11)	ManagementShow preview for(9)		
Decision MakingShow preview for(57)	Sustainable Value ChainShow preview for(20)	Competitive AdvantageShow preview for(14)	LeadershipShow preview for(11)	Manufacturing CompaniesShow preview for(9)		
Sustainable Value CreationShow preview for(54)	Value Co-creation (20)	Risk ManagementShow preview for(14)	MarketingShow preview for(11)	ModelShow preview for(9)		
Life CycleShow preview for(50)	Climate ChangeShow preview for(19)	ValuationShow preview for(14)	OptimizationShow preview for(11)	NonhumanShow preview for(9)		
ManufactureShow preview for(45)	Energy EfficiencyShow preview for(19)	Value PropositionShow preview for(14)	PerformanceShow preview for(11)	ResearchShow preview for(9)		
StakeholderShow preview for(40)	Triple Bottom LineShow preview for(19)	Corporate StrategyShow preview for(13)	RecyclingShow preview for(11)	SurveysShow preview for(9)		
Corporate Social ResponsibilityShow preview for(38)	Value ChainShow preview for(19)	CostsShow preview for(13)	Sustainable AgricultureShow preview for(11)	Theoretical StudyShow preview for(9)		
EconomicsShow preview for(37)	Business ModelShow preview for(18)	EducationShow preview for(13)	Value EngineeringShow preview for(11)	United StatesShow preview for(9)		
PlanningShow preview for(36)	DesignShow preview for(18)	Energy UtilizationShow preview for(13)	ArchitectureShow preview for(10)	Chemical IndustryShow preview for(8)		
ArticleShow preview for(35)	Eco-efficiencyShow preview for(18)	Environmental SustainabilityShow preview for(13)	ChinaShow preview for(10)	ChemistryShow preview for(8)		
Business ModelsShow preview for(35)	Economic AnalysisShow preview for(18)	European Union (13)	Corporate-sustainabilityShow preview for(10)	Co-creationShow preview for(8)		
CompetitionShow preview for(33)	EfficiencyShow preview for(18)	MethodologyShow preview for(13)	Customer SatisfactionShow preview for(10)	Construction IndustryShow preview for(8)		
Economic And Social EffectsShow preview for(33)	HumansShow preview for(18)	Product-service SystemsShow preview for(13)	Decision Support SystemsShow preview for(10)	Crop ProductionShow preview for(8)		
Supply Chain ManagementShow preview for(33)	MappingShow preview for(18)	Social AspectsShow preview for(13)	EcodesignShow preview for(10)	Environmental ValuesShow preview for(8)		
Circular EconomyShow preview for(32)	Sustainability PerformanceShow preview for(18)	StakeholdersShow preview for(13)	EnvironmentShow preview for(10)	Food WasteShow preview for(8)		
Product DesignShow preview for(32)	Sustainable Business ModelShow preview for(18)	Supply ChainShow preview for(13)	Environmental EconomicsShow preview for(10)	Industrial EconomicsShow preview for(8)		
Environmental ImpactShow preview for(31)	BenchmarkingShow preview for(17)	Sustainable Development GoalsShow preview for(13)	Governance ApproachShow preview for(10)	Information UseShow preview for(8)		
HumanShow preview for(31)	BiomassShow preview for(17)	Environmental TechnologyShow preview for(12)	Industrial ManagementShow preview for(10)	Life Cycle AssessmentShow preview for(8)		
Supply ChainsShow preview for(30)	Environmental ProtectionShow preview for(17)	IndustryShow preview for(12)	Lean Production (10)	LigninShow preview for(8)		
Sustainable BusinessShow preview for(28)	ManufacturingShow preview for(17)	InvestmentShow preview for(12)	Manufacturing ProcessShow preview for(10)	Literature ReviewsShow preview for(8)		
Sustainable ManufacturingShow preview for(28)	Project ManagementShow preview for(17)	PerceptionShow preview for(12)	Product DevelopmentShow preview for(10)	Performance MeasurementShow preview for(8)		
Value ChainsShow preview for(25)	SalesShow preview for(17)	Sustainable Business ModelsShow preview for(12)	ReviewShow preview for(10)	Production EfficiencyShow preview for(8)		
Corporate GovernanceShow preview for(24)	Business DevelopmentShow preview for(16)	Value Stream MappingShow preview for(12)	Social And EnvironmentalShow preview for(10)	Questionnaire SurveyShow preview for(8)		
Literature ReviewShow preview for(24)	EcologyShow preview for(16)	Alternative AgricultureShow preview for(11)	Social ResponsibilityShow preview for(10)	Research WorkShow preview for(8)		
Industry 4.0Show preview for(23)	Knowledge ManagementShow preview for(16)	Artificial IntelligenceShow preview for(11)	Assessment MethodShow preview for(9)	Resource UseShow preview for(8)		
InvestmentsShow preview for(22)	Performance AssessmentShow preview for(16)	CarbonShow preview for(11)	ChainsShow preview for(9)	Sharing EconomyShow preview for(8)		
Business Model InnovationShow preview for(21)	Strategic ApproachShow preview for(16)	Carbon DioxideShow preview for(11)	Conceptual FrameworksShow preview for(9)	Stakeholder Engagement		

Sustainable health (1149)					popular journals for the key word
HumanShow preview for(600)	HumanShow preview for(600)	ChildShow preview for(43)	Sustainable HealthShow preview for(29)	Surveys And QuestionnairesShow preview for(21)	Sustainability SwitzerlandShow preview for(28)
HumansShow preview for(471)	HumansShow preview for(471)	Developing CountriesShow preview for(43)	AfricaShow preview for(28)	Health EquityShow preview for(20)	Studies In Health Technology And InformaticsShow preview for(18)
ArticleShow preview for(339)	ArticleShow preview for(339)	Financial ManagementShow preview for(43)	CanadaShow preview for(28)	Health ExpendituresShow preview for(20)	BMC Health Services ResearchShow preview for(17)
Health Care DeliveryShow preview for(175)	Health Care DeliveryShow preview for(175)	LeadershipShow preview for(43)	Health Care OrganizationShow preview for(28)	ManagementShow preview for(20)	Frontiers In Public HealthShow preview for(17)
FemaleShow preview for(154)	FemaleShow preview for(154)	Quality Of LifeShow preview for(43)	Health PersonnelShow preview for(28)	National Health ServiceShow preview for(20)	International Journal Of Environmental Research And Public Health (14)
Health Care PolicyShow preview for(145)	Health Care PolicyShow preview for(145)	AgedShow preview for(42)	Risk FactorShow preview for(28)	PoliticsShow preview for(20)	
MaleShow preview for(139)	MaleShow preview for(139)	Health EducationShow preview for(42)	Attitude To HealthShow preview for(27)	Program DevelopmentShow preview for(20)	
Public HealthShow preview for(131)	Public HealthShow preview for(131)	Health ServicesShow preview for(41)	LearningShow preview for(27)	WellbeingShow preview for(20)	
Organization And ManagementShow preview for(124)	Organization And ManagementShow preview for(124)	MethodologyShow preview for(41)	PsychologyShow preview for(27)	Clinical PracticeShow preview for(19)	
Sustainable DevelopmentShow preview for(123)	Sustainable DevelopmentShow preview for(123)	OrganizationShow preview for(41)	Public Health ServiceShow preview for(27)	Coronavirus Disease 2019Show preview for(19)	
AdultShow preview for(121)	AdultShow preview for(121)	Global HealthShow preview for(40)	Carbon FootprintShow preview for(26)	Cost ControlShow preview for(19)	
Health CareShow preview for(118)	Health CareShow preview for(118)	International CooperationShow preview for(40)	CurriculumShow preview for(26)	Focus GroupsShow preview for(19)	
Health PromotionShow preview for(118)	Health PromotionShow preview for(118)	World Health OrganizationShow preview for(39)	ExerciseShow preview for(26)	Health WorkforceShow preview for(19)	
Delivery Of Health CareShow preview for(114)	Delivery Of Health CareShow preview for(114)	AdolescentShow preview for(36)	Follow UpShow preview for(26)	Information SystemsShow preview for(19)	
Health Care SystemShow preview for(109)	Health Care SystemShow preview for(109)	Health Care AccessShow preview for(36)	United KingdomShow preview for(26)	NonhumanShow preview for(19)	
HealthShow preview for(93)	HealthShow preview for(93)	Health StatusShow preview for(36)	Conservation Of Natural ResourcesShow preview for(25)	PrevalenceShow preview for(19)	
SustainabilityShow preview for(93)	SustainabilityShow preview for(93)	Program EvaluationShow preview for(36)	Cross-Sectional StudiesShow preview for(25)	Randomized Controlled TrialShow preview for(19)	
Priority JournalShow preview for(92)	Priority JournalShow preview for(92)	Qualitative ResearchShow preview for(36)	Health Care ReformShow preview for(25)	Social ResponsibilityShow preview for(19)	
Health PolicyShow preview for(87)	Health PolicyShow preview for(87)	Decision MakingShow preview for(35)	Risk AssessmentShow preview for(25)	Statistics And Numerical DataShow preview for(19)	
ReviewShow preview for(87)	ReviewShow preview for(87)	Health ProgramShow preview for(35)	Chronic DiseaseShow preview for(24)	Systematic ReviewShow preview for(19)	
Sustainable HealthsShow preview for(79)	Sustainable HealthsShow preview for(79)	Physical ActivityShow preview for(35)	EditorialShow preview for(24)	CooperationShow preview for(18)	
Health Care CostShow preview for(78)	Health Care CostShow preview for(78)	StandardsShow preview for(33)	Environmental SustainabilityShow preview for(24)	Health Care NeedShow preview for(18)	
Health Care PlanningShow preview for(77)	Health Care PlanningShow preview for(77)	Cross-sectional StudyShow preview for(32)	EuropeShow preview for(24)	Health Knowledge, Attitudes, PracticeShow preview for(18)	
EconomicsShow preview for(76)	EconomicsShow preview for(76)	Health Services AccessibilityShow preview for(32)	Health Services ResearchShow preview for(24)	PovertyShow preview for(18)	
Health ServiceShow preview for(76)	Health ServiceShow preview for(76)	PandemicShow preview for(32)	FundingShow preview for(23)	Quality Of Health CareShow preview for(18)	
Health Care PersonnelShow preview for(69)	Health Care PersonnelShow preview for(69)	Patient CareShow preview for(32)	Health Care UtilizationShow preview for(23)	Resource AllocationShow preview for(18)	
Health Care QualityShow preview for(69)	Health Care QualityShow preview for(69)	Community CareShow preview for(31)	HospitalsShow preview for(23)	StandardShow preview for(18)	
ProceduresShow preview for(67)	ProceduresShow preview for(67)	Cost Effectiveness AnalysisShow preview for(31)	Practice GuidelineShow preview for(23)	State MedicineShow preview for(18)	
United StatesShow preview for(66)	United StatesShow preview for(66)	Health SystemsShow preview for(31)	Health Care FinancingShow preview for(22)	DemographyShow preview for(17)	
Controlled StudyShow preview for(61)	Controlled StudyShow preview for(61)	InterviewShow preview for(30)	Medical InformaticsShow preview for(22)	Diabetes MellitusShow preview for(17)	
GovernmentShow preview for(59)	GovernmentShow preview for(59)	Medical EducationShow preview for(30)	ObesityShow preview for(22)	DietShow preview for(17)	
Climate ChangeShow preview for(55)	Climate ChangeShow preview for(55)	Environmental ProtectionShow preview for(29)	SocioeconomicsShow preview for(22)	ForecastingShow preview for(17)	
Middle AgedShow preview for(55)	Middle AgedShow preview for(55)	Health BehaviorShow preview for(29)	Total Quality ManagementShow preview for(22)	Health Personnel AttitudeShow preview for(17)	
EducationShow preview for(53)	EducationShow preview for(53)	Health Care FacilityShow preview for(29)	Young AdultShow preview for(22)	Health PlanningShow preview for(17)	
Primary Health CareShow preview for(51)	Primary Health CareShow preview for(51)	Information ProcessingShow preview for(29)	Capacity BuildingShow preview for(21)	Health SurveyShow preview for(17)	
AustraliaShow preview for(48)	AustraliaShow preview for(48)	Medical Researchr(29)	ConsumerShow preview for(21)	InnovationShow preview for(17)	
QuestionnaireShow preview for(48)	QuestionnaireShow preview for(48)	Outcome Assessment 29	EpidemiologyShow preview for(21)	LifestyleShow preview for(17)	
Major Clinical StudyShow preview for(45)	Major Clinical StudyShow preview for(45)	Human ExperimentShow preview for(44)	InvestmentShow preview for(21)	MotivationShow preview for(17)	
Developing CountryShow preview for(44)	Developing CountryShow preview for(44)	COVID-19Show preview for(43)	PandemicsShow preview for(21)	PhysicianShow preview for(17)	
Health Insurance	Health InsuranceShow preview for(44)		Social Determinants Of Health	Policy	

"digital sustainability"					journals
Digital SustainabilityShow preview for(28)	Business ModelsShow preview for(2)	SoftwareShow preview for(2)	Analog To Digital ConversionShow preview for(1)	Business EthicsShow preview for(1)	Sustainability SwitzerlandShow preview for(15)
SustainabilityShow preview for(28)	COVID-19Show preview for(2)	StakeholderShow preview for(2)	App StoresShow preview for(1)	Business Model CanvasShow preview for(1)	Digital Policy Regulation And GovernanceShow preview for(4)
Sustainable DevelopmentShow preview for(27)	CoronavirusShow preview for(2)	StudentShow preview for(2)	Application FunctionsShow preview for(1)	Business Model InnovationShow preview for(1)	International Journal Of Information ManagementShow preview for(3)
Artificial IntelligenceShow preview for(5)	Corporate StrategyShow preview for(2)	SurveysShow preview for(2)	Application InstallationsShow preview for(1)	COVID-19 PandemicShow preview for(1)	Lecture Notes In Computer Science Including Subseries Lecture Notes In Artificial Intelligence And Lecture Notes In BioinformaticsShow preview for(3)
Digital TransformationShow preview for(5)	Cultural HeritageShow preview for(2)	Sustainable DigitalizationShow preview for(2)	Application ProgramsShow preview for(1)	Case-studiesShow preview for(1)	Sustainability ScienceShow preview for(3)
DigitalizationShow preview for(5)	Digital AgeShow preview for(2)	TeachingShow preview for(2)	Arab WorldShow preview for(1)	Change ManagementShow preview for(1)	
Literature ReviewShow preview for(5)	Digital CommunicationShow preview for(2	TransformationShow preview for(2)	ArticleShow preview for(1)	Changfeng Culture Business CoreShow preview for(1)	
Big DataShow preview for(4)	Digital DevicesShow preview for(2)	United NationsShow preview for(2)	Artificial Neural NetworkShow preview for(1)	ChinaShow preview for(1)	
Circular EconomyShow preview for(4)	Digital EconomyShow preview for(2)	Virtual RealityShow preview for(2)	Assessment ToolShow preview for(1)	Citizen EngagementsShow preview for(1)	
Corporate Social ResponsibilityShow preview for(4)	Digital EducationShow preview for(2)	WebsitesShow preview for(2)	Audio AcousticsShow preview for(1)	Civil AviationShow preview for(1)	
Digital StorageShow preview for(4)	Digital MediaShow preview for(2)	World Wide WebShow preview for(2)	Audio And VideoShow preview for(1)	ClassificationShow preview for(1)	
Digital TechnologyShow preview for(4)	Digital ResourcesShow preview for(2)	2030 AgendaShow preview for(1)	Augmented RealityShow preview for(1)	Classification (of Information)Show preview for(1)	
Life CycleShow preview for(4)	Digital SocietyShow preview for(2)	4th Industrial RevolutionShow preview for(1)	Automotive Software ArchitectureShow preview for(1)	Climate ActionShow preview for(1)	
Climate ChangeShow preview for(3)	Digital TechnologiesShow preview for(2)	#ESDfor2030Show preview for(1)	Aviation 4.0Show preview for(1)	Climate ActionsShow preview for(1)	
Decision MakingShow preview for(3)	DigitizationShow preview for(2)	Academic Literature	AwarenessShow preview for(1)	Climate InformationShow preview for(1)	
Digital PreservationShow preview for(3)	E-governmentShow preview for(2)	Accident PreventionShow preview for(1)	Axiomatic DesignShow preview for(1)	Climate ResilienceShow preview for(1)	
E-learningShow preview for(3)	EcologyShow preview for(2)	AccommodationShow preview for(1)	Bar CodeShow preview for(1)	Climate-conscious CitizenShow preview for(1)	
EducationShow preview for(3)	Environmental ImpactShow preview for(2)	Accounting Information System (AIS)Show preview for(1)	Best PracticesShow preview for(1)	CloudShow preview for(1)	
GermanyShow preview for(3)	European UnionShow preview for(2)	Accounting ProfessionShow preview for(1)	Bibliometric AnalysisShow preview for(1)	Cluster AnalysisShow preview for(1)	
Higher EducationShow preview for(3)	Green ITShow preview for(2)	Accuracy AssessmentShow preview for(1)	Bio-basedShow preview for(1)	College StudentsShow preview for(1)	
Industry 4.0Show preview for(3)	HumanShow preview for(2)	Action PlanShow preview for(1)	BiobankShow preview for(1)	CommunicationShow preview for(1)	
Information TechnologyShow preview for(3)	Knowledge Based SystemsShow preview for(2)	Action ResearchShow preview for(1)	BiobasedShow preview for(1)	Communication InfrastructureShow preview for(1)	
Information UseShow preview for(3)	LearningShow preview for(2)	AddictionsShow preview for(1)	Bioeconomy	Communication NetworkShow preview for(1)	
InnovationShow preview for(3)	ManufactureShow preview for(2)	Adoption FactorsShow preview for(1)	BioethicsShow preview for(1)	Communication TechnologiesShow preview for(1)	
PandemicShow preview for(3)	Numerical ModelShow preview for(2)	Adoption Of TechnologyShow preview for(1)	BioplasticShow preview for(1)	Comparative AnalysisShow preview for(1)	
Smart CityShow preview for(3)	PlanningShow preview for(2)	AdultShow preview for(1)	BitcoinShow preview for(1)	CompetencesShow preview for(1)	
Social SustainabilityShow preview for(3)	Primary EducationShow preview for(2)	AfricaShow preview for(1)	BlendeShow preview for(1)	Comprehensive Decision On Collective Well-beingShow preview for(1)	
Sustainable Development GoalsShow preview for(3)	Qualitative ResearchShow preview for(2)	AgedShow preview for(1)	BlenderShow preview for(1)	Computational SustainabilityShow preview for(1)	
Technological DevelopmentShow preview for(3)	RecyclingShow preview for(2)	Agricultural MachineShow preview for(1)	BlendingShow preview for(1)	Computer Aided InstructionShow preview for(1)	
Assessment MethodShow preview for(2)	Smart CitiesShow preview for(2)	AgricultureShow preview for(1)	Brandenburg [Germany]Show preview for(1)	Computer ProgrammingShow preview for(1)	
AustraliaShow preview for(2)	Social MediaShow preview for(2)	Air TransportationShow preview for(1)	BrazilShow preview for(1)	Computer Software ReusabilityShow preview for(1)	
BiobankingShow preview for(2)	Social NetworksShow preview for(2)	Amazonas [Brazil]Show preview for(1)	British LibraryShow preview for(1)		

	TITLE-ABS-KEY (sustainab*) 874,162				popular journals	
Sustainable DevelopmentShow preview for(229,879)	Land UseShow preview for(14,506)	IndiaShow preview for(9,211)	AustraliaShow preview for(7,367)	Project ManagementShow preview for(6,259)	Sustainability Switzerland	28256
SustainabilityShow preview for(119,569)	BiodiversityShow preview for(14,439)	Solar EnergyShow preview for(9,028)	CompetitionShow preview for(7,330)	Compressive StrengthShow preview for(6,223)	Iop Conference Series Earth And Environmental Science	14715
ArticleShow preview for(65,229)	EcologyShow preview for(14,115)	Wastewater TreatmentShow preview for(8,964)	CropsShow preview for(7,191)	ResearchShow preview for(6,219)	Journal Of Cleaner Production	12498
HumanShow preview for(59,566)	AdultShow preview for(14,042)	Water ResourcesShow preview for(8,837)	Emission ControlShow preview for(7,082)	GeneticsShow preview for(6,204)	Advanced Materials Research	6089
HumansShow preview for(44,418)	Water SupplyShow preview for(13,258)	Fossil FuelsShow preview for(8,784)	Public PolicyShow preview for(6,937)	Comparative StudyShow preview for(6,174)	E3s Web Of Conferences	4692
Climate ChangeShow preview for(33,827)	InnovationShow preview for(13,025)	Energy PolicyShow preview for(8,723)	ProductivityShow preview for(6,930)	Food SupplyShow preview for(6,163)	Science Of The Total Environment	4048
PlanningShow preview for(31,043)	ProceduresShow preview for(12,960)	GroundwaterShow preview for(8,661)	Supply ChainsShow preview for(6,892)	TechnologyShow preview for(6,123)		
ChinaShow preview for(28,384)	ForestryShow preview for(12,645)	DesignShow preview for(8,612)	Strategic PlanningShow preview for(6,872)	Environmental MonitoringShow preview for(6,120)		
Decision MakingShow preview for(27,545)	Greenhouse GasesShow preview for(12,633)	ConservationShow preview for(8,541)	ManagementShow preview for(6,828)	GISShow preview for(6,041)		
Environmental ImpactShow preview for(26,704)	CostsShow preview for(12,303)	Economic AnalysisShow preview for(8,538)	Circular EconomyShow preview for(6,814)	Governance ApproachShow preview for(6,041)		
EconomicsShow preview for(25,813)	OptimizationShow preview for(12,103)	EfficiencyShow preview for(8,532)	Environmental PolicyShow preview for(6,745)	Urban GrowthShow preview for(5,978)		
Environmental ProtectionShow preview for(25,434)	EducationShow preview for(11,902)	EnergyShow preview for(8,480)	Life Cycle AssessmentShow preview for(6,737)	Population StatisticsShow preview for(5,901)		
NonhumanShow preview for(21,461)	EcosystemsShow preview for(11,891)	Information ManagementShow preview for(8,452)	Carbon FootprintShow preview for(6,662)	SoilShow preview for(5,892)		
Energy EfficiencyShow preview for(21,335)	Developing CountriesShow preview for(11,576)	Renewable EnergyShow preview for(8,448)	UrbanizationShow preview for(6,640)	AsiaShow preview for(5,867)		
Priority JournalShow preview for(21,070)	InvestmentsShow preview for(11,521)	WaterShow preview for(8,417)	Economic GrowthShow preview for(6,614)	GovernmentShow preview for(5,861)		
AgricultureShow preview for(20,740)	Risk AssessmentShow preview for(11,430)	Nitrogen	MethodologyShow preview for(6,589)	StudentsShow preview for(5,849)		
Controlled StudyShow preview for(20,281)	AnimalShow preview for(11,395)	Conservation Of Natural ResourcesShow preview for(8,162)	CatalysisShow preview for(6,585)	IrrigationShow preview for(5,809)		
FemaleShow preview for(19,287)	Energy ConservationShow preview for(11,103)	Gas EmissionsShow preview for(8,145)	Water PollutionShow preview for(6,571)	CelluloseShow preview for(5,795)		
Carbon DioxideShow preview for(18,977)	ChemistryShow preview for(11,093)	Renewable EnergiesShow preview for(8,129)	Food SecurityShow preview for(6,567)	Numerical ModelShow preview for(5,778)		
BiomassShow preview for(18,432)	Waste ManagementShow preview for(11,016)	Water ConservationShow preview for(8,117)	United KingdomShow preview for(6,549)	HealthShow preview for(5,757)		
Energy UtilizationShow preview for(17,490)	Economic DevelopmentShow preview for(10,470)	Environmental EconomicsShow preview for(8,102)	Regional PlanningShow preview for(6,539)	COVID-19Show preview for(5,730)		
Life CycleShow preview for(16,546)	Water QualityShow preview for(10,284)	ManufactureShow preview for(7,974)	PollutionShow preview for(6,535)	PerformanceShow preview for(5,717)		
MaleShow preview for(16,052)	Urban PlanningShow preview for(10,218)	Sustainable AgricultureShow preview for(7,932)	TemperatureShow preview for(6,502)	Agricultural RobotsShow preview for(5,706)		
Economic And Social EffectsShow preview for(15,968)	EnvironmentShow preview for(10,012)	Urban AreaShow preview for(7,874)	Cultivation	RiversShow preview for(5,690)		
Environmental ManagementShow preview for(15,814)	SoilsShow preview for(9,912)	Construction IndustryShow preview for(7,812)	Unclassified DrugShow preview for(6,456)	ChildShow preview for(5,688)		
Environmental SustainabilityShow preview for(15,711)	MetabolismShow preview for(9,902)	Public HealthShow preview for(7,799)	BiofuelsShow preview for(6,433)	PhysiologyShow preview for(5,666)		
Water ManagementShow preview for(15,343)	Renewable Energy ResourcesShow preview for(9,732)	EurasiaShow preview for(7,691)	Cost Benefit AnalysisShow preview for(6,410)	Urban DevelopmentShow preview for(5,644)		
ReviewShow preview for(15,161)	CommerceShow preview for(9,555)	StakeholderShow preview for(7,601)	Quality ControlShow preview for(6,390)	BiofuelShow preview for(5,641)		
RecyclingShow preview for(15,139)	Global WarmingShow preview for(9,546)	SurveysShow preview for(7,600)	Scanning Electron MicroscopyShow preview for(6,336)	Health Care DeliveryShow preview for(5,629)		
United StatesShow preview for(14,946)	Remote SensingShow preview for(9,498)	EcosystemShow preview for(7,486)	PerceptionShow preview for(6,309)	Spatiotemporal AnalysisShow preview for(5,537)		
CarbonShow preview for(14,582)	Cost EffectivenessShow preview for(9,414)	Environmental TechnologyShow preview for(7,452)	AfricaShow preview for(6,283)	HousingShow preview for(5,536)		
AnimalsShow preview for(14,554)	EuropeShow preview for(9,339)	Organization And ManagementShow preview for(7,388)	Forest ManagementShow preview for(6,261)	Ecosystem Service		
